# Morphological Variation in the Rice Root Nematode, *Hirschmanniella Oryzae* (Van Breda De Haan, 1902) Luc & Goodey, 1964 from Korea, with Inferences from its Ribosomal and Mitochondrial DNA

**DOI:** 10.2478/jofnem-2022-0055

**Published:** 2022-12-10

**Morphological Variation in the Rice Root Nematode, *Hirschmanniella Oryzae* (Van Breda De Haan, 1902) Luc & Goodey, 1964 from Korea, with Inferences from its Ribosomal and Mitochondrial DNA**

Mwamula^1^, T. H. Lim^3^, Y. Kim, H. Lee^2^, Y. H. Kim^1^ and D. W. Lee^1,2^

^1^Department of Entomology

^2^Department of Ecological Science, Kyungpook National University, Sangju, Republic of Korea

^3^Nousbo Co., Ltd., Suwon, Republic of Korea

## Abstract

A population of *Hirschmanniella oryzae* with extreme morphological and morphometric variations was reported from rhizospheric soils sampled from a paddy rice-garlic double cropped farm in Korea. Females and males are morphologically characterized and their linked DNA sequence data supplied. Morphological and morphometric data revealed extreme intraspecific variation in characters such as body length, stylet length, stylet knob shape, tail length, tail terminus shape and body ratios, among others. But, despite the significant morphological and morphometric divergence, inferences from the analyses of the 18S-rRNA, D2-D3 expansion segment of 28S-rRNA and ITS-rRNA partial sequences confirmed that the studied population belongs to *H. oryzae*. The current morphometric data extends total ranges recorded for *H. oryzae*, but also, overlaps all morphometric characters of *H. belli*. However, the available molecular DNA markers (18S- and 28S-rRNA) strongly support the validity of the two species. Therefore, *H. belli* is herein considered a cryptic species within the *H. oryzae* group.

**A Multi-State Effort to Contain and Manage *Meloidogyne Enterolobii* in Vegetable Crops**

Agudelo, Paula^1^, J. Corbin^1^, J. Desaeger^2^, A. Gorny^3^, Z. Grabau^4^, Z. Guan^2^, H. Scherm^5^, J. Mueller^1^, L. Quesada-Ocampo^3^, W. Rutter^6^, and P. Wadl^6^

^1^Clemson University, Dept. of Plant and Environmental Sciences, Clemson, SC 29634

^2^University of Florida Gulf Coast Research and Education Center, Dept. of Entomology and Nematology, Wimauma, FL 33598

^3^North Carolina State University, Dept. of Entomology and Plant Pathology, Raleigh, NC 27695

^4^University of Florida, Dept. of Entomology and Plant Pathology, Gainesville, FL 32611

^5^University of Georgia, Dept. of Plant Pathology, Athens, GA 30602

^6^USDA-ARS, Charleston, SC 24914

## Abstract

FIND*Me* (Focused Investigations on the Distribution and Management of *Meloidogyne enterolobii*) is a project sponsored by the USDA Specialty Crop Research Initiative. Research partners include Clemson University, North Carolina State University, the University of Georgia, USDA-ARS and the University of Florida. *Meloidogyne enterolobii* (*M.e*.), a highly virulent root-knot nematode (RKN) species, is potentially devastating to specialty crop production in the southeastern United States. This species can impact yield and quality, and its quarantined status jeopardizes interstate and international trade. *M.e*. can infect and damage crop genotypes that are resistant to the other major species of RKN, including sweetpotato, cucumber, watermelon, and tomato. Our goal is to reduce the vulnerability of growers to the emerging agricultural threat posed by *M.e*. by using a systems-based approach involving five research and Extension objectives: 1) Study the prevalence and distribution of *M.e*. in vegetable crops in the Southeast, and characterize the genetic variability present; 2) Evaluate and develop vegetable germplasm with resistance to *M.e*.; 3) Evaluate the efficacy of nematicides, cover crops, and rotations as management strategies; 4) Assess the costs and returns of management tactics such as rotations, cover crops, and nematicides for the mitigation of *M.e*. on sweetpotato, cucumber, watermelon and tomato crops; 5) Develop print and web-based educational materials on management and containment strategies for *M.e*. Education of commercial growers and home gardeners is critical in preventing the spread and managing *M.e*. Systematic surveying of symptomatic crops has led to identification of *M.e*. in multiple counties in Florida and North Carolina, two counties in South Carolina, and two counties in Georgia.

**Evaluation of Nematicides for Management of Plant-Parasitic Nematodes in Sugarcane**

Aguilar, Iris M. and T. Watson

Department of Plant Pathology and Crop Physiology, Louisiana State University Agricultural Center, Baton Rouge, LA 70803

## Abstract

In a soil survey conducted in 2020 which included 62 sugarcane fields in Louisiana, the plant-parasitic nematode genera that were commonly detected included *Tylenchorhynchus* (Stunt), *Criconemella* (Ring), *Helicotylenchus* (Spiral), and *Pratylenchus* (Lesion). These nematodes represent an underestimated threat to this economically important crop. In this project, field soil was collected from 7 commercial sugarcane fields in Louisiana. A subsample of soil from each site was used to quantify the abundance of plant-parasitic nematodes before the application of any treatment. The remaining soil was used in a potted greenhouse experiment using the following four soil treatments: (1) untreated soil (control), (2) sterilized soil, (3) Mocap 15G at 26 lb/A (active ingredient: ethoprop), and (4) Nimitz at 7 pints/A (active ingredient: fluensulfone). Liquid nematicide treatments were applied to soil as a 25-mL drench five days prior to planting with a single eyepiece of HoCP 96-540. The granular nematicide treatment (Mocap 15G) was applied to the soil surface and tilled to a depth of 10 cm. Plants were grown in a greenhouse for three months prior to analysis. In three out of seven of the soils, plant biomass and/ or tillering was greater in pots that had been sterilized relative to that of untreated soil. Nematicide application did not significantly impact plant growth in any of the seven soils. Lesion nematode was the most abundant nematode detected in sugarcane roots. For all seven soils, root parasitism by lesion and stunt nematode was lower in soil that had been sterilized relative to that of untreated soil. In one field soil, application of Mocap 15G decreased root parasitism by lesion nematode. In another field soil, application of Nimitz decreased the abundance of stunt nematode in soil. Overall, our data suggest that soil application of nematicides shows the potential to be a component of an integrated nematode management strategy for sugarcane in Louisiana.

**Performance of Southern Root-Knot and Reniform Nematode Resistant Cotton Varieties in Fields Infested with Columbia Lance and Lesion Nematodes**

Ahmed, Saleh, M. Plumblee and J. Mueller

Clemson University, Dept. Plant and Environmental Sciences, Edisto REC, Blackville, SC 29817

## Abstract

This study was conducted in 2021 at Clemson University’s Edisto Research and Education Center. The objective was to examine the performance of recently released cotton varieties resistant to *Meloidogyne incognita* (Southern root- knot nematode) and *Rotylenchulus reniformis* (reniform nematodes) in fields infested with *Hoplolaimus columbus* (Columbia lance nematode: CLN) and *Pratylenchus spp*. (Lesion nematodes: LN). Columbia lance and lesion nematodes occur very commonly in fields in the Coastal Plains of South Carolina. Columbia lance nematode occurs more commonly than reniform nematode in cotton and soybean fields in South Carolina. Six cotton varieties were planted in each of two fields with different infestation levels of CLN, LN, and RKN. Fields were designated “High Pressure” and “Low Pressure” based on at-plant levels of CLN. Split-plots were created with each plot consisting of eight 50-ft long rows on 38-inch centers. Each eight row main plot consisted of one of six varieties. Plots were split with 4 rows treated with 3.0 gal./acre of Telone II and 4 rows left nontreated. Root-knot/reniform resistant varieties included: DP 2141 B2XF, PHY 443 W3FE, and PHY 500 W3FE and susceptible varieties included: DP 1646 B2XF, NG 5711 B2XF, NG 5007 B2XF. Application of Telone II significantly (P=0.05) reduce mean LN populations across varieties 88% and 97% compared to the nontreated plots in the high- and low-pressure fields, respectively. Application of Telone II reduced mean CLN populations across varieties 92% and 69% in the high- and low-pressure fields, respectively compared to the nontreated plots. Telone II was also highly effective in increasing the yield of all varieties in the two fields, with mean yield increases of 140 and 50 lbs./acre more than the non-treated plots in the high- and low-pressure fields respectively. The resistant varieties appeared to have a lower yield potential than the susceptible varieties in both the Telone II treated and nontreated plots. Across both fields, mean yield of susceptible varieties treated and nontreated with Telone II yielded 119.7 lbs. lint/acre more than the mean yield of the resistant varieties resistant varieties treated and nontreated with Telone II. Mean yield across both fields of the susceptible varieties was 955.3 lbs. lint/acre treated with Telone II and 838.1 lbs. of lint per acre when nontreated. Resistance to Southern root-knot and reniform nematodes was not expected to affect infection and damage from species such as CLN and LN and that is what we observed for these varieties in these fields. If use of the resistant varieties can minimize infection and yield losses from root-knot and reniform nematodes then the plants may be more open to infection and yield losses by other species including migratory endoparasites such as CLN and LN. Future management schemes will need to take into account this new scenario.

**Screening of Sweetpotato Plant Introductions for Resistance to Reniform Nematode**

Alam, Md Shah^1^, C. Khanal^1^, W. Rutter^2^ and P. Wadl^2^

^1^Clemson University, Department of Plant and Environmental Sciences, 105 Collings St., Clemson, SC 29634

^2^USDA-ARS, U.S. Vegetable Laboratory, 2700 Savannah Hwy., Charleston, SC 29449

## Abstract

The reniform nematode is highly damaging to many economically important crops including sweetpotato. Current management method for reniform nematode in sweetpotato relies on the use of fumigants, although season-long protection is difficult to achieve with the use of fumigants. Because reniform nematode resistance is lacking in currently available sweetpotato cultivars, current project screened 24 sweet potato Plant Introductions (PI) against reniform nematode in a greenhouse environment. PI 153907, PI 595869, and PI 599386 significantly suppressed reniform nematode eggs per root system, the suppression respectively being 91%, 88%, and 89% compared to the Beauregard (control). The PI 153907 also suppressed reniform nematode vermiform life stages by 69% compared to the control. Additionally, reniform nematode eggs per gram of dry root was significantly suppressed by PI 153907 and PI 595869, the suppression being 92% and 93%, respectively. Results form current study suggest that PI 153907, PI 595869, and PI 599386 are promising lines that can be used for resistance breeding program.

**Effects of Thermotherapy on Reproduction Biology of *Meloidogyne Enterolobii***

Alarcon-Mendoza, Ivan and C. Khanal

Clemson University, Dept. of Plant and Environment Science, Clemson, SC 29634

## Abstract

Experiment was conducted to determine the effects of thermotherapy on the reproduction biology of the guava root-knot nematode (*Meloidogyne enterolobii*), the most damaging species in the genus *Meloidogyne*. Suspensions of freshly hatched second-stage juveniles (J2) of *M. enterolobii* were placed in 1.5 mL Eppendorf tubes and exposed to hot water at 42 °C and 44 °C for 60 minutes in a ThermoMixer^®^ C. Hot water treated 500 J2 were inoculated into three-week-old tomato seedlings (cv. Rutgers) grown in 6-inch plastic pots. Nematodes without hot water treatment served as untreated control. Each of the three treatments (heat-treated at 42 °C and 44 °C, and untreated control) was replicated five times in a randomized complete block design. Nematode egg production per root system was significantly reduced for the ones that were heat-treated at 44 °C, the reduction being 70% compared to the control. Egg production of nematodes treated at 42 °C was statistically similar to that of control. Nematodes heat-treated at 44 °C had 77% less J2 per 100 cm^3^ of soil compared to the control. Similarly, heat treatment of 42 °C resulted in 41% lower J2 per 100 cm^3^ of soil, although the reduction was not significantly different from the control. Any significant differences in plant biomass were not observed among the plants inoculated with heat treated and untreated nematodes. Further examination of nematode eggs suggested that the nematodes treated at 42 °C and 44 °C produced eggs that had a significantly lower hatch rate in relation to the control. The reduction in egg hatch percentage was 32% and 34% for the nematodes treated at 42 °C and 44 °C, respectively. Results from this experiment suggest that heat treatment does not only affects the reproduction of *M. enterolobii*, but also the effects of heat treatment are translated to the next generation.

**The Role of Semiochemicals in Entomopathogenic Nematode Behavior, Cracking the Code**

Alborn, Hans T.^1^, A. M. Gaffke^1^, D. Shapiro-Ilan^2^, F. Kaplan^3^ and E. Lewis^4^

^1^USDA-ARS Center for Medical, Agricultural, and Veterinary Entomology, USDA-ARS Gainesville, FL

^2^USDA-ARS, Southeastern Fruit and Tree Nut Research Laboratory, Byron, GA

^3^Pheronym, Inc., Davis, CA

^4^University of Idaho, Department of Entomology, Plant Pathology and Nematology, Moscow, ID

## Abstract

Entomopathogenic nematodes (EPNs) can be utilized for below ground control of insect pests on agricultural crops and to some extent also for above ground control. EPNS utilize pheromones for behaviors such as mating, dispersal, and aggregation. When searching for a host insect, infective juvenile EPNs (IJ) also respond to kairomones, which are released by the host or by the plant the hosts are feeding on. We have previously discovered that IJs can be trained to respond to a variety of volatile semiochemicals, commonly released by insect infested plants (e.g., terpenoids). However, we don’t fully understand the relative importance, or hierarchical order, of host versus host related semiochemicals. Furthermore, for successful mass infection of a potential host, EPNs also display characteristic behaviors, such as follow the leader, but the nature of the species specific semiochemicals involved in these behaviors are still not fully understood. This presentation will focus on our present understanding of how semiochemicals regulate EPN behavior and how we can use chemical ecology to further improve the use of EPNs for control of insect pests on agricultural crops. We will present our progress on sampling, and chemical identifications and will discuss how these semiochemicals might be used to improve the efficacy and host specificity of artificially reared as well as native EPNs.

**Evaluation of Nematocidal Potential of *Azadirachta Indica and Cannabis Sativa* in the Management of *Meloidogyne Incognita* in Tomato**

Azeem, Wajahat^1,2^, T. Mukhtar^2^, M. Inam-ul-Haq^2^, M. A. Khan^2^, T. Hamid^3^, H. Regmi^1^ and L. W. Duncan^1^

^1^Entomology and Nematology Department, University of Florida, Citrus Research and Education Center (CREC), FL 33850

^2^Department of Plant Pathology, PMAS-Arid Agriculture University Rawalpindi, Pakistan, 46300

^3^Department of Zoology, University of Gujrat, Pakistan, 50700

## Abstract

Root-knot nematodes (RKN; *Meloidogyne* spp.) seriously reduce production in many crop species worldwide. Among the known root-knot species, *Meloidogyne incognita* is the most widely distributed. Restrictions on nematicide use due environmental and human health concerns has increased interest in alternative means of managing nematodes. We investigated the effectiveness of aqueous extracts of two plants native to Pakistan, *Azadirachta indica* and *Cannabis sativa*, at reducing *Meloidogyne incognita* hatch, survival, and plant infection at concentrations between 0-100%. Both plants inhibited egg hatch, increased juvenile mortality and inhibited infection of tomato cv. Money Maker in a dose dependent manner, with *A. indica* significantly outperforming *C. sativa*. Both extracts also significantly reduced number of galls, egg masses, eggs per mass, and reproduction factor of the nematode, again in a dose-dependent manner with *A. indica* being most effective. Thus, both plants have potential as alternatives for control of root-knot nematodes. A similar study of combinations of *Moringa oleifera* leaf extract and plant growth promoting rhizobacteria native to Florida for control of *M. incognita* in tomato cv HM1824 is ongoing.

**Nemataxa: A New Taxonomic Database for Analysis of Nematode Community Data**

Baker, Hannah^1^, J. Caballero^2^, C. Gleason^1^, C. Jahn^2^, C. Hesse^3^, J. Stewart^2^ and I. Zasada^3^

^1^Washington State University, Dept. of Plant Pathology, Pullman, WA 99163

^2^Colorado State University, Dept. of Agricultural Biology, Ft. Collins, CO 80523

^3^USDA-ARS, Corvallis, OR 97330

## Abstract

High throughput amplicon sequencing of nematode communities has the potential to greatly increase our understanding of nematode community ecology. A current constraint to the widespread implementation of amplicon sequencing is the lack of sequence databases with consistent taxonomic naming schemes. Focusing on 18S sequence data, we developed NemaTaxa, a manually curated database that can be used with QIIME and mothur analysis platforms. Nematode 18S sequence data was downloaded from NCBI from which both Nematoda universal primers NF1 and 18Sr2b aligned. Taxonomic strings were trimmed to include only classical Linnaean lineages to genera within Nematoda; missing taxonomic data were completed manually. NemaTaxa was compared with other available databases, specifically PR2 and Silva v132, available for mothur using data collected from Oregon, Idaho, and Washington potato cropping systems. In general, NemaTaxa performed similar to PR2 in the number of contigs assigned to Nematoda and estimates of diversity. NemaTaxa resolves classification at the genus, family and order levels while PR2 always has a portion of sequences assigned at the class level due to incomplete taxonomic strings. The Silva v132 database available in mothur is of limited use unusable because of the greatly reduced number of nematode sequences available in the database, making classification only possible to the level of order. NemaTaxa offers an “off the shelf” database that can be used by nonexperts in nematology wanting to explore nematode community ecology, and therefore, will allow for inclusion of nematodes in soil ecology studies that employ amplicon sequencing for other organisms such as fungi and bacteria.

**Role of Callose Deposition in Plant-Nematode Interactions**

Bali, Sapinder, H. Peng and C. Gleason

Washington State University, Department of Plant Pathology, Pullman, WA 99164

## Abstract

Root-knot nematodes (*Meloidogyne* spp.) secrete hundreds of molecules called effectors into plants to facilitate parasitism during compatible interaction. We previously characterized an effector from *Meloidogyne hapla* called Mh265. This pioneer effector was up regulated in expression during pre- and early parasitic life stages. In addition, the Mh265 cDNA localized to the esophageal glands of the nematode, indicating that it is a secreted protein. Transgenic Arabidopsis plants that expressed Mh265 were more susceptible to nematode infections. Typically, when plants encounter a pathogen (such as a nematode) or a defense elicitor, their basal defenses are triggered, and callose deposition is a component of the basal defense response. The current hypothesis is that nematode infections can trigger basal plant defense responses, including callose deposition, but the nematodes secrete effectors that can suppress these defense responses and, ultimately, facilitate nematode infections. We have previously shown that Mh265 can suppress an elicitor-induced callose response. In an effort to understand how Mh265 can suppress callose deposition, we further studied Mh265 and callose deposition in plants. We found that although Mh265 suppresses the callose deposition in Arabidopsis, it does not directly interact with the plant callose synthases. To determine if callose deposition plays any significant role in nematode resistance, we treated wild type Arabidopsis plants with 2-deoxy-D-glucose (DDG), an inhibitor of callose synthesis, and interestingly the plants were more susceptible to *M. hapla* infection as compared to the untreated controls. We next wanted to determine if callose deposition was an important aspect of the plant-nematode interaction in more agronomically important plants. Because *M. hapla* is a problem on potato in the Pacific Northwest, we looked at the role of callose in the potato-nematode interaction. We generated stable potato transgenic lines that were knocked down in *StGSL5* expression, potato homolog of the callose synthase gene, which encodes the major contributor to defense-related callose deposition. These plants have significant reduction in callose synthase gene expression, and they are more susceptible to *M. hapla* infections as compared to the wild type. Although it is still not clear how Mh265 suppresses callose deposition, we have been able to better define the relationship between callose deposition and plant susceptibility to root-knot nematodes. The long-term goal will be to use this information to manipulate the deposition of callose or other natural defenses to prevent nematode infection specifically in the roots.

**Insight into Genomes of Steinernema Nematodes**

Baniya, Anil^1^, E. M. Schwarz^2^ and A. R. Dillman^1^

^1^Department of Nematology, University of California-Riverside, 900 University Ave., Riverside, CA 92521

^2^Department of Molecular Biology and Genetics, Cornell University, Biotechnology 407, Ithaca, NY 14853

## Abstract

Entomopathogenic nematodes (EPNs) are a group of small soil-dwelling roundworms that can kill insects and are used as biological control agents in agriculture. EPNs partner with symbiotic bacteria to kill and reproduce within an insect host. EPNs are used to study different aspects of biology such as host-parasite interactions, neurobiology, evolution, and symbiosis. Their usefulness as a model system has been hampered by the lack of genetic tools. The rediscovery of the species *Steinernema hermaphroditum*, which consistently reproduces as self-fertilizing hermaphrodites, provides a new opportunity for the development of genetic tools in this species, which could possibly be expanded to other *Steinernema* nematodes. Toward this end, we used MinION sequencing technology to sequence and assemble the genome of *S. hermaphroditum*. The final genome of *S. hermaphroditum* consists of 5 scaffolds, matching the observed chromosome number, with a scaffold N50 of 17.7 Mb and GC content of 47.1%, resulting in a genome of approximately 87.9 Mb. We also have completed preliminary assemblies for two other EPN species: *S. glaseri* and *S. scapterisci*. We will discuss the current state of steinernematid genomes and what we have learned about their biology through comparative genome analyses. The genomes provide a foundation for functional genetic research that will increase the value of EPNs as model systems.

**Predicting Specific Cyst Nematode Suppression in California Sugar Beet Soils**

Becker, Jennifer Smith^1^, J. Borneman^2^, P. Ruegger^2^ and J. O. Becker^1^

^1^Dept. of Nematology, University of California, Riverside, CA 92521

^2^Dept. of Microbiology and Plant Pathology, Riverside, CA 92521

## Abstract

Previously our group identified several *Heterodera schachtii*-suppressive soils in sugar beet and Brassica fields in southern and coastal California, respectively. The primary causal fungi were likely members of the *Dactylella oviparasitica* (syn. *Brachyphoris oviparasitica*) clade, belonging to the anamorphic family Orbiliaceae, recently included in the teleomorphic genus *Hyalorbilia*. Infestation of various agricultural soil samples with strain *Hyalorbilia* aff. multiguttulata DoUCR50, followed by inoculation with J2 of *H. schachtii* and planting with cabbage seedlings, established cyst nematode-suppressive conditions. DoUCR50 parasitizes several life stages of *H. schachtii* and is likely responsible for this population suppression. Baiting with *H. schachtii* females and Illumina sequencing revealed the presence of nematophagous *Hyalorbilia* spp. in 21 of 25 randomly selected sugar beet field soils in California's Imperial Valley. Similar results from coastal broccoli fields indicated a widespread occurrence of these fungi in California. We posited that detection of high levels of indigenous *Hyalorbilia* spp. in continuous or narrow host crop rotations would be correlated to nematode population suppression. In another survey during the sugar beet harvest in 2019, tare soil samples from 44 different sugar beet fields were collected at the sugar beet processing plant in Brawley, CA. Members of the *H. oviparasitica* clade were consistently detected in 13 but not in 5 soils when 6 measurements were taken for each soil using a sequence-selective nested TaqMan qPCR assay. The other soils produced variable results across the 6 measurements. We randomly selected 7 of the 13 soils that consistently tested positive for the presence of *Hyalorbilia* spp clade members and two soils which consistently tested negative. Each soil sample was divided into two parts, one non-treated and one autoclaved. Conical tubes were each filled with 200 cm^3^ portions of either soil, seeded with cabbage, and three weeks later infested with 500 J2 of *H. schachtii*. The eighteen treatments were arranged in a randomized complete block design with four replications. The experiment was conducted twice for 12 weeks (approximately 1,430 degree days, base temperature 8˚C). In the combined ANOVA analysis, numbers of white to light brown cyst females in soils with detectable *H. oviparasitica* clade signal were reduced to 1/3 to 1/6 compared to autoclaved, *H. schachtii* re-infested controls. Only *Hyalorbilia* spp. were cultured from the female nematodes, providing evidence of parasitism in the suppressive soils. The two soils with no *H. oviparasitica* qPCR signal showed no suppressive effect compared to the controls. In conclusion, qPCR detection of the *H. oviparasitica* clade in sugar beet tare soil was predictive of *H. schachtii* population-suppressive activity. The hyperparasitic fungi are widespread in *H. schachtii*-infested soils and are likely to suppress population build-up of the cyst nematodes in the presence of their host plants.

**Leaf Lesions on American Ginseng Associated with a Foliar Nematode, *Aphelenchoides* sp., In Tennessee**

Bernard, E.C.^1^, E. Lopez^1^, Y. Gao^2^ and E. Swiggart^2^

^1^University of Tennessee, Entomology and Plant Pathology, Knoxville, TN, USA

^2^Middle Tennessee State University, International Ginseng Institute, Murfreesboro, TN, USA

## Abstract

The ginseng genus *Panax* (Araliaceae) contains 18 species, of which the Asian species *P. ginseng* and the North American species *P. quinquefolius* are best known. The roots of both taxa are said to have beneficial and medicinal effects, and both are widely cultivated, especially in Asia. However, wild-harvested ginseng is thought to be more potent and commands a much higher price than cultivated plants; therefore, both species have become uncommon or rare in their native ranges due to overharvesting. In the U.S. significant efforts are made to protect wild *P. quinquefolius* populations from poachers, but occurrence of disease in small, scattered populations could pose a further threat to the viability of the species. Ginseng is a suitable host for many soil-inhabiting plant-parasitic nematodes, and foliar nematode (*Aphelenchoides* spp.) infestations have been reported from both ginseng species: one species on *P. ginseng* (Russia) and four on *P. quinquefolius* (China). In 2021 a Tennessee commercial grower reported leaf spots on several *P. quinquefolius* plants. These leaves were harvested and examined. Leaf tissue maceration in water yielded multiple female, male and juvenile individuals of an *Aphelenchoides* sp. Specimens were processed to glycerin and permanently mounted on slides in glycerin. Morphological observations and measurements were made of 7 females and 2 males and compared to those of the many members of this genus, with special attention to the species already known from ginseng: *A. dactylocercus*, *A. helicus*, *A. panaxofolia*, *A. parabicaudatus* (China) on *P. quinquefolius* and *A. panaxi* (Russia) on *P. ginseng*. The Tennessee specimens appear to be distinct from these five as well as other *Aphelenchoides* spp. in having three lateral incisures, stylet length of 12–13 μm and digitate or irregular tail with or without a pointed apex. During the 2022 growing season, we will survey the original site as well as cultivated and wild ginseng stands. If foliar nematodes are collected they will be cultured on fungi to obtain sufficient material for experimental studies, LM and SEM examination, and comparative molecular analysis to clarify the status of this nematode.

**Relationships among *Pratylenchus Penetrans*, Soil Health Indicators and Soil Fumigation in Michigan Potato Production**

Bird, G. W.

Michigan State University

## Abstract

Twelve soil health indicators and *Pratylenchus penetrans* were determined for geo-positioned soil samples taken before potato planting from three fields in each of six Michigan commercial potato farms in 2012. Most of the six farms used a two-year rotation with potato and corn or carrots. Nine of the eighteen fields were fumigated the previous fall. On a scale of 0-100, the mean soil health indicator score was 56.3. The farm with the highest soil health indicator scores used a five-year crop rotation that included two cover crops and five cash crops. Mean *P. penetrans* population densities were 53.3 and 4.7 per 100 cm^3^ soil for the non-fumigated and fumigated sites respectively. Soil health scores (0-100), water stable aggregates (%), active carbon (ppm) and nitrogen mineralization potential (μgN/ gdwsoil/week) were significantly lower in fumigated, compared to non-fumigated fields. In fumigated fields, there was a significant negative relationship between *P. penetrans* and active carbon, the highest *P. penetrans* population densities were associated with relatively high levels of water stable aggregate stability and there was no apparent relationship between *P. penetrans* and nitrogen mineralization potential. The survey was repeated in 2022, using the 2012 geo-referenced soil sampling points to investigate changes in soil health associated with Michigan’s potato industry over the past decade. In general, *P. penetrans* population densities were lower in 2022, compared to 2012. In addition, there were changes in nematode management including types, rates and placement of nematicides, and modifications in crop rotations and use of cover crops.

**Assessment of Anguinidae Nematodes in Oregon Grasses for Seed & Golf Industries**

Braithwaite, Emily T.^1^, A. Kowalewski^1^, I. Zasada^2^ and H. Rivedal^3^

^1^Oregon State University, Dept. of Horticulture, Corvallis, OR 97331

^2^USDA-ARS HCRU, Corvallis, OR 97331

^3^USDA-ARS FSCRU, Corvallis, OR 97331

## Abstract

Oregon is the grass seed capital of the world, supplying more than 70% of all grass seed produced across the globe. Oregon is also home to many golf courses, with these industries providing a combined total economic impact for the state of several billion dollars annually. In both grass seed production and the golf course industry, plant-parasitic nematodes, specifically *Anguina agrostis*, *Anguina funesta*, and *Anguina pacificae*, and other *Anguina* spp., have resulted in significant revenue losses and associated management costs. In seed production, positive tests of seed containing *Anguina* spp. has led to an increase in international export rejections at substantial costs to seed companies. In the golf industry, *A. pacificae* has been identified on coastal Oregon golf courses, and is contributing to considerable costs for both applications of nematicides, and labor and management for recovery from damage. There is a lack of foundational research in the literature and reliable threshold levels for the many *Anguina* spp. identified in the Pacific Northwest. The purpose of this research is to optimize the diagnostic sample timing, collection techniques, and extraction methodology for *Anguina* spp. in Oregon. Sample collections from both seed production fields and golf courses have been initiated to evaluate nematode recovery methods by comparing different collection materials (soil, tillers, seeds) and extraction methods (mist chambers, Baermann funnels, sieving or grinding plus sugar flotation, and tissue soaking) of Anguinidae as well as other plant-parasitic nematodes. In addition, molecular diagnostic tools will be evaluated for their utility in both the grass seed and golf turf systems. Method evaluations will occur year-round to identify the best timing for Anguinidae detection and risk assessment to determine whether management strategies should be deployed. In addition, broader surveys of grass seed fields (20-25 per year) and golf courses (10 per year) will identify the overall diversity and population density of plant parasitic nematodes that are present. From this research, detection tools and identification tutorials for diagnosticians will be developed, as well as training for stakeholders on the optimum nematode sample timing and collection technique. This work will help both industries understand a highly yield-limiting nematode issue to tailor sustainable, integrated management strategies to each grower’s or golf course’s needs.

**Papaya Ground Seed as a Biofumigant against Soil-Borne Pathogens in Hawai’i**

Braley, Lauren E. and K.-H. Wang

University of Hawaii at Manoa, Honolulu, HI 96822

## Abstract

Papaya (*Carica papaya*) seeds are usually considered an agricultural waste in Hawaii but contain benzyl isothiocyanate (BITC), a biofumigant potentially suppressive to many soil-borne pathogens. An *in vitro* experiment using potato dextrose agar (PDA) plates each with a 2-cm diameter well filled with 1) 1 g ground papaya seed (PGS)+1 ml dH2O, 2) 0.5 g PGS + 0.5 ml dH2O, and 3) nothing (control) was conducted to examine biofumigant effects of PGS against *Fusarium oxysporum*, *Fusarium solani*, or *Setophoma sp*. using cell plugs incubated for 48 hours. PGS biofumigant reduced the growth of *F. oxysporum* effectively, but only reduced that of *F. solani* slightly, with no effect on the growth of *Setophoma sp*. Three greenhouse pot trials were then conducted to examine suppressive effects of (PGS) against root-knot nematodes (*Meloidogyne incognita*) and *Fusarium oxysporum*. *Fusarium*-infested soil collected from a commercial field was amended with 1) 0.5% PGS, 2) 1% PGS, 3) 0.5% PGS+0.5% PGS crude extract [PGS+CE], 4) 1% ‘Calientee 199’ brown mustard (*Brassica juncea*) (BM), 5) not amended (NA), and compared to 6) an autoclaved control (Auto). Each treatment was replicated 4 times. ‘Manoa’ lettuce seedlings were transplanted in Trial I and Trial III whereas ‘Hirayama’ kai choi were transplanted in Trial II as bioassay crops. In all treatments, except for the autoclaved control, 100 *M. incognita* infective juveniles were inoculated per pot at planting and the experiments were terminated 1 month after nematode inoculation. All PGS and BM treatments suppressed root galls on lettuce (Trial I and III). In Trial II, 1% PGS and PGS+CE also reduced root penetration of root-knot nematodes in kai choi based on acid fuchsin staining. *Fusarium* recovery from roots plated on Komada selective medium was also reduced by all PGS and BM treatments compared to NA. A field trial was performed at a *Fusarium*-infested kai choi farm. Six preplant soil treatments installed were: 1) Vapam (methyl isothiocyanate) fumigation, 2) sorghum cover crop (Sg), 3) brown mustard (BM) amendment, 4) 0.5% PGS, 5) 1% PGS, and 6) untreated control. Amendment rate was calculated based on 7.6-cm trench/30-cm row spacing. Each treatment was replicated in 4 plots (1.2 × 3 m2) arranged in randomized complete block design. Kai choi seedlings were transplanted 1 week after soil amendment and grown for 6 weeks. PGS along with Sg and BM failed to suppress Fusarium wilt on kai choi with similar disease incidence as the untreated control. Though Vapam suppressed Fusarium wilt effectively, it compromised soil health as it significantly reduced nematode richness, free-living nematode abundance, in particular the bacteria-feeding nematodes compared to the untreated control. Current research confirmed the biofumigation potential of PGS against root-knot nematodes and *F. oxysporum* in vitro or in pots. More research is needed to deliver PGS biofumigants in the field while improving its suppressive effects against *F. oxysporum* and plant-parasitic nematodes alongside maintaining or improving soil health conditions.

**Susceptibility and Host Potential of Six Cucurbit Crops to *Meloidogyne Enterolobii*, *M. Floridensis*, *M. Hapla*, *M. Incognita* and *M. Javanica***

Bui, Hung and J. Desaeger

University of Florida, Department of Entomology and Nematology, Gulf Coast Research and Education Center, Wimauma, FL 33598

## Abstract

Cucurbits are economically important crops in Florida. They are also very susceptible to root-knot nematodes (*Meloidogyne* spp., RKN), which are very common in the tropical and subtropical climate and sandy soils of Florida, and many different species are found in the state. A series of greenhouse and field trials were conducted to evaluate the susceptibility and host potential of different cucurbit crops to different RKN. In the greenhouse, we tested the sensitivity of six cucurbit crops (squash, cucumber, cantaloupe, watermelon, smooth and angled luffa) that are commonly grown in Florida to some of the major RKN species, *Meloidogyne enterolobii*, *M. floridensis*, *M. hapla*, *M. incognita* and *M. javanica* under greenhouse conditions. The first experiment showed that *M. incognita* was more damaging to the six tested cucurbit crops than *M. javanica* and *M. floridensis* in terms of gall index, eggs per root system, eggs per gram root and reproduction factor. In experiment 2, cucurbit crops suffered greater damage and allowed higher reproduction of *M. enterolobii* as compared to *M. javanica*. The more cryophilic species *Meloidogyne hapla* caused relatively little damage to all six cucurbit crops and had low reproduction rates. Highest root gall ratings and reproduction was noted for cucumber and cantaloupe, and lowest for watermelon and squash. In general, the thermophilic RKN species caused higher root gall ratings on cucumber and watermelon than on squash, cantaloupe and luffa. This was also noted in the field trials, where cucumber had consistently higher gall indices than squash and zucchini. Root-knot reproduction rates were the highest on squash and cucumber, especially with *Meloidogyne incognita* and *M. enterolobii*. While all cucurbits were good hosts for especially the thermophilic RKN species, significant differences were noted among cucurbit crops and RKN species. This information could help cucurbit growers more effectively manage RKN in their fields.

**Evaluation of Allyl Isothiocyanate (Dominus™) and Crustatacean Meal (Crablife Flake™) on Soil Microbiome and Plant-Parasitic Nematodes on Pineapple**

Chellemi, D.^1^, K. Barber^2^ and B. Sipes^3^

^1^Agricultural Solutions LLC, Lake Placid, FL 33852

^2^Dole Food Company Hawaii, Honolulu, HI 96819

^3^University of Hawaii, Plant and Environmental Protection Sciences, Honolulu, HI 96822

## Abstract

Plant-parasitic nematodes are problems in pineapple production in Hawaii. Management typically involves fallow followed by fumigation with 1,3-dichloropropene (1,3-D) with unintended effects on soil health. The objective was to determine effects of allyl isothiocyanate and crustacean meal to stimulate soil biological activity. A trial was established in a commercial field in collaboration with Dole Fruit Hawaii. CrabLife Flake (25% chitin) was applied at 0 or 785 kg/ha and incorporated. 1,3-D (300 l/ha) and allyl isothiocyanate (290 l/ ha) were applied in the bed. An unfumigated, no CrabLife Flake area served as an untreated control. Soil samples were collected at pineapple planting, 3 months, and 6 months after planting. The ratio of fungi to bacteria in the at-planting samples was greatest in the untreated control and the fumigated plots receiving the CrabLife Flake. Plant-parasitic nematode populations were near 0/250 cm^3^ soil at planting and 3 months after planting. At 6 months after planting, the population of reniform nematodes remained low in the 1,3-D treated plots (133/250 cm^3^ soil) and increased in the untreated and allyl isothiocyanate treated plots (650 and 733/250 cm^3^ soil, respectively). Fungal feeding nematodes predominated over other free-living nematodes. Plant growth was not different among the treatments. The chitin amendment had positive effects on the fungi in the soil. Allyl isothiocyanate was effective in reducing plant-parasitic nematode populations for the first 3 months and may offer an alternative to improve soil health in pineapple production.

**Exploration of Wild Potato Species for Novel Resistance against Potato Cyst Nematodes**

Chen, Shiyan^1^, H. Yang^2^ and X. Wang^1,2^

^1^School of Integrative Plant Science, Cornell University, Ithaca, NY 14853

^2^Robert W. Holley Center for Agriculture and Health, USDA-ARS, Ithaca, NY 14853

## Abstract

Potato cyst nematodes (PCN) (*Globodera rostochiensis* and *G. pallida*) are internationally recognized quarantine pests and pose a serious threat to potato production worldwide. Yield losses exceeding 80% may occur when PCN is left uncontrolled. The U.S. potato industry has been adversely affected by the emergence of *G. pallida* in Idaho and a virulent pathotype of *G. rostochiensis*, Ro2, in New York because of the lack of control options. Coupled with quarantine regulations, crop rotation utilizing resistant potato cultivars is the most effective and sustainable means for controlling PCN. However, there are currently no U.S. potato cultivars with resistance to *G. pallida*, and cultivars with resistance against Ro2 are very limited. Wild potato species provide an extremely rich source of natural resistance against plant pathogens, including PCN. We used bioassays to screen approximately 250 accessions of 5 wild potato species obtained from the U.S. Potato Genebank against PCN. Subsequently, we identified a group of wild potato clones that showed strong resistance to Ro2 or broad-spectrum resistance to both PCN species. Through *in-vitro* infection assays, we further assessed the response of the identified resistant clones to PCN infection using microscopy. The results indicated that the resistance exhibited by certain clones may be mediated by resistance (*R*) genes. For example, one resistant clone of *Solanum brevicaule* (named Y1-5) showed a hypersensitive response (HR) when a conserved effector from PCN was transiently expressed in the leaves. To clone the potential *R* gene(s) present in Y1-5, we conducted genetic crosses using Y1-5 and a susceptible *S. brevicaule* clone as parents and subsequently obtained a mapping population. The progeny are being evaluated to determine segregation of resistant and susceptible phenotypes via *in-vitro* root infection assay and the leaf HR assay using the conserved PCN effector gene. Once phenotypic data are collected, we will utilize a RenSeq (Resistance gene Enrichment Sequencing) approach coupled with long-read PacBio SMRT sequencing to map and clone the corresponding *R* gene(s) in Y1-5. Our study has demonstrated that the collection of wild potato species at the U.S. Potato Genebank is an excellent source to search for novel resistance against PCN and that nematode effectors may be used as a molecular tool for rapid discovery of *R* gene(s) in wild potato germplasm. The cloning of the novel *R* genes and the introduction of the identified resistant potato germplasm into U.S. potato breeding programs may accelerate breeding potatoes for durable PCN resistance.

**Foliar Nematode Control on Ornamental Fern with Reduced Risk Nematicides and Cross Check on Phytotoxicity Risk of Several New Nematicides**

Cheng, Zhiqiang and K. Mitsuda

Department of Plant and Environmental Protection Sciences, CTAHR, University of Hawaii at Manoa, Honolulu, HI 96822

## Abstract

*Aphelenchoides fragariae* is a species of foliar nematode that is an increasingly widespread pathogen of ornamental plants with a wide host range, attacking more than 250 plants species in 47 plant families. The most recognizable symptom of foliar nematode infection is the interveinal lesions on leaves. Previously, chemical treatments using active ingredients (a.i.) such as oxamyl and parathion were effective against foliar nematodes. However, due to environmental concerns and their potentially high toxicity, these chemicals are no longer available for foliar nematode control. The overall goal of this project was to determine the effectiveness of several new, reduced-risk nematicides against foliar nematodes on several popular ornamental plants in Hawaii. Specific objectives were to determine: 1) the efficacy of several new nematicides for managing foliar nematodes on palapalai fern, *Microlepia strigosa*; and 2) if these new nematicides have phytotoxicity on commonly grown ornamental plants in Hawaii: *Microlepia strigosa*, *Frangipani*, *Rhaphiolepis indica*, *Hibiscus*, *Phalaenopsis*, and *Anthurium andraeanum*. Foliar nematodes were extracted from infected fern tissues using the Baermann funnel method. These nematodes were cultured in the lab using carrot discs and the cultures were refreshed every 5-7 weeks prior to inoculation onto *M. strigosa*. New nematicides ESP 715 (a.i. fluopyram), MBI 304 (a.i. *Chromobacterium* spp. strain extract), and Majestene (a.i. *Burkholderia* spp. strain extract) were tested against *Aphelenchoides fragariae* on *M. strigosa*. Foliar nematode abundance was measured prior to treatments and at the end of the experiment. Foliar nematode damage severity (0-5 scale) was measured weekly post treatment for 6 weeks. In addition, ESP 715, MBI 304, and Majestene were examined for phytotoxicity on *M. strigosa*, *Frangipani*, *R. indica*, *Hibiscus*, *Phalaenopsis*, and *A. andraeanum* at various rates. All experiments included a no chemical control. Tested plants received three applications of the designated nematicides at 14-day intervals in the phytotoxicity experiment. Our results indicated that fluopyram did not significantly suppress foliar nematode abundance, but did reduce foliar nematode damage severity. *Burkholderia* and *Chromobacterium* did not statistically suppress foliar nematode abundance but did reduce the numbers by 65.7% and 75.8%, respectively. *Burkholderia* and *Chromobacterium* also reduced foliar nematode damage severity. No visual foliar phytotoxicity symptoms were observed under any treatments throughout the study, except for fluopyram on *M. strigosa* (visible phytotoxicity, but low at 1 on a 1-5 phytotoxicity scale). These results suggested that *Burkholderia* and *Chromobacterium* could potentially be used to manage *A. fragariae* on *M. strigosa*.

**Nematicidal Effect of *Muscodor Crispans* Compounds on Root-Knot and Root-Lesion Nematodes**

Consoli, Erika, A.T. Dyer and G.A. Strobel

Montana State University, Dept. of Plant Sciences and Plant Pathology, Bozeman, MT 59717

## Abstract

Plant-parasitic nematodes are a substantial problem for agricultural production worldwide. These nematodes are managed by applying chemical or biological nematicides, planting nematode resistant cultivars, and adopting cultural practices such as periodic fallow on the nematode-affected areas. Unfortunately, these controls are often insufficient due to the wide host ranges and the prolonged survival of some nematode species, the limited availability of nematode resistant cultivars, and the withdrawal of nematicides from the market due to toxicological considerations. Thus, a need exists for the discovery of new, novel, safe, and effective nematicides. To this end, we have discovered a product termed formula B to be a safe and effective nematicide. Formula B is a biorational product whose composition is based on volatile compounds produced by the fungus *Muscodor crispans*. The effect of formula B on the motility and vitality of two major plant-parasitic nematodes, *Meloidogyne* sp. and *Pratylenchus* sp., was tested in *in vitro* and *in vivo*. In *in vitro* studies, nematodes were exposed to a concentration series of 0, 0.125, 0.25, 0.5 and 1% of formula B, and were evaluated for motility and viability after 30min, 1, 4, 6, 24 and 30 hours after exposure. Death was confirmed by exposing nematodes to 1M NaOH and observing nematodes for movements. For both nematodes, normal movement was no longer recorded after 4h of exposure to formula B regardless of the concentration, and after 30 h, each of the 0.125, 0.25, 0.5 0.5 and 1% resulted in 100% mortality rate. *Meloidogyne* intestinal content was degraded at 24h after exposure to formula B. In *in planta* studies, approximately 750 *Meloidogyne* specimens were inoculated in 250-cc pots containing a mixture of a sandy loam soil. A treatment of 10-ml of formula B treatments (0, 0.125, 0.25, 0.5 and 1% concentration) was added into the respective pots each day for two days after nematode inoculation (dai). A nematode free treatment was included to examine formula B’s phytotoxicity. On the fifth dai, a two-week old cucumber seedling was transplanted into each pot. Root gall index and total root length was recorded at 30 days after transplanting. For root lesion nematodes, approximately 260 mixed stages of *Pratylenchus* sp. were inoculated into 66-cc conestainers. At one dai, cones received 3-ml of formula B treatments (i.e; 0, 0.125, 0.25, 0.5 and 1% concentration). At two dai, a four-day old wheat seedling was transplanted into each cone. *P. neglectus* penetration was evaluated at 10 dai by staining wheat roots with acid fuchsin and counting nematodes per root system. *Pratylenchus* invasion in wheat seedlings was statistically different among treatments (*p*-value < .001). The 0.5 and 1% concentrations of formula B reduced nematode penetration by 70% compared to the untreated control. Formula B also reduced gall formation in cucumber plants. These results demonstrate the potential of Formula B as a potent nematicide.

**Advances on Winter Wheat Breeding for the Management of the Root Lesion Nematode, *Pratylenchus Neglectus***

Consoli, Erika, J. Johnston and A.T. Dyer

Montana State University, Dept. of Plant Sciences and Plant Pathology, Bozeman, MT 59717

## Abstract

A statewide nematode survey conducted in 2006 - 2007 revealed high *P. neglectus* densities in Montana. At the time, nematode densities varied widely across the state with highest densities being primarily associated with winter wheat production. On average, densities were 3390 and 4045 *P. neglectus*/kg soil in 2006 and 2007, respectively. Through comparisons with yield response curves, crop losses due to this nematode in Montana’s winter wheat were estimated at 15% of total yield, representing an estimated US$60 – 70 million in economic losses annually. To address these losses, resistant rotational crops (i.e., pea, lentils, and barley) were identified in field trials. In the following years, these rotational crops became widely adopted in winter wheat producing regions of Montana. Nearly ten years after the initial survey and the implementation of resistant crop rotations, a follow up survey was conducted in 2015-2016. The goal was to reassess the status of root lesion nematode in Montana fields, and to evaluate the impact of crop rotations on *P. neglectus* densities. This survey revealed that *P. neglectus* was present in 75% of the sampled fields, but densities were at one tenth of those previously found (i.e., mean of 268 and 377 *P. neglectus*/kg soil in 2015 and 2016, respectively). Despite the overall trend, moderately high population densities were observed at several locations where *P. neglectus* pathotypes capable of multiplying in resistant rotational crops were detected. In a follow up survey conducted in 2021, *P. neglectus* densities averaged 5715 ±924 *P. neglectus*/kg soil. These high densities had not been observed in Montana since the initial surveys, heightening Montana’s need for locally adapted nematode resistant cultivars - a long-term priority of our program. For this, successive crosses using Persia-20 - a reported nematode resistant breeding line to both *P. neglectus* and *P. thornei* - and Montana adapted winter wheat varieties were performed. Recurrent selections for resistance and agronomic performance resulted in 23 resistant/ tolerant double haploid lines with acceptable agronomics traits (i.e.: yield, protein, and plant height). Of these, four have superior yield relative to widely grown Montana cultivars. This fall, the performance of top resistant and susceptible lines will be evaluated in *P. neglectus* infested fields across the state. Given the synergies between root lesion nematodes and other root pathogens, we expect plant resistance to *P. neglectus* will also improve overall root health. To evaluate this impact, we will assess the differences in the rhizosphere microbiome and the incidence of other root pathogens associated with resistant and susceptible winter wheat lines in three *P. neglectus* infested fields and later in inoculated greenhouse trials.

**The use of Seed-Applied Nematicides and Insecticides to Manage Early Season Cotton Pests**

Cooke, Claire, S.H. Graham and K.S. Lawrence

Auburn University, Dept. of Entomology and Plant Pathology, Auburn, AL 36849

## Abstract

Management of early season pests, such as seedling diseases, thrips, and plant parasitic nematodes, is critical for optimizing cotton management. Nematodes alone can cause annual yield losses of over 4% in the USA. In Alabama, the root knot nematode (*Meloidogyne incognita*) and the reniform nematode (*Rotylenchulus reniformis*) are important in various locations across the state. Due to the ease of application and relative efficacy, nematodes are often managed using seed-applied products. The objective of this study was to evaluate the effects of early season cotton health in nematode infested fields with various insecticide/nematicide seed-applied treatments. Field trials arranged as a RCBD with four or five replications were established in central (root-knot) and north (reniform) Alabama. At both locations, a susceptible variety, DP 1646 B2XF was planted at a seeding rate of 3 seeds per foot. Treatments included 1) Fungicide Only check, 2) Gaucho (imidacloprid) insecticide seed treatment (IST), 3) Aeris (imidacloprid + thiodicarb) IST, 4) Gaucho IST + Poncho/Votivo (clothianidin + *Bacillus firmus* I-1582), and 5) Gaucho IST + COPeO Prime (fluopyrgam). Regardless of location, cotton treated with an IST showed less thrips injury than the fungicide only control. Similarly, cotton treated with an IST tended to have better vigor and greater above and below ground biomass than the fungicide only control. In central Alabama, where the root knot nematode is the primary species, there were no differences in nematode population levels at 26 days after planting (DAP). In north Alabama, where the reniform nematode is the primary species, no differences in nematode populations were observed at 29 DAP.

**Investigating the Virulence Types of Soybean Cyst Nematode Populations in Indiana using a Modified HG Type Test**

Critchfield, Ricky and L. Zhang

Department of Botany and Plant Pathology, Purdue University, West Lafayette, IN 47907

## Abstract

Soybean cyst nematode (SCN, *Heterodera glycines*) is one of the most important pathogens of soybean in the US, causing annual yield losses estimated at more than $1.2 billion. Soybean varieties with the source of resistance from PI 88788 have been frequently used for combating SCN. Unfortunately, virulent SCN populations have been reported, which can overcome the resistance and reduce the effectiveness of soybean varieties with PI 88788-derived resistance to control SCN. An SCN virulence type (HG type) test using 7 soybean plant introduction (PI) lines with different sources of resistance has been utilized to identify SCN virulent populations and determine which lines of soybean resistance could effectively control the virulent SCN population in soybean fields. Virulence profiling of SCN populations has not been done in Indiana for over the past 10 years; therefore, another analysis of SCN virulence in the state can provide updated information to the soybean growers. We received 124 soil samples from soybean growers in Indiana between September and December 2020. SCN eggs were found in all the soil samples (100% positive), a significant increase compared to a previous report in 2010, which estimated that 45% of soybean fields in Indiana might be infested with SCN. Representative SCN populations based on county locations were selected for the virulence type test. The 7 standard indicator lines and a new indicator line, PI 567516C, were used to test the virulence of SCN populations with the Williams 82 as the susceptible check. We have tested 23 SCN populations using the modified HG test. Our results showed that all the 23 SCN populations tested can overcome the PI 88788-type resistance, and 3 of SCN populations can also break the Peking-type resistance. The previous SCN survey conducted between 2006 and 2008 in Indiana revealed that only 56% of SCN populations tested were virulent on PI 88788, and no population was found to be virulent to Peking. Furthermore, 2 SCN populations are virulent to the new indicator line PI 567516C. The SCN survey clearly indicated that virulent SCN populations in Indiana are widely distributed in soybean fields, which is consistent with reports of SCN virulence types from several other soybean-producing states in the US. Frequent soil sampling for SCN egg counts and virulence types can provide field-specific data to help soybean growers to manage this devastating pest of soybean in an evolving landscape.

**Efficacy of Zelto™ and Crescendo™ against *Meloidogyne Graminis* on Golf Course Bermudagrass**

Crow, William T.^1^ and Bret Corbett^2^

^1^Entomology and Nematology Department, University of Florida, Gainesville, FL.,

^2^Albaugh LLC, Ankeny, IA

## Abstract

The active ingredient of Zelto is a killed microbial fermentation product of *Burkholderia* spp. strain A396. The active ingredient of Crescendo is a killed fermentation product of *Chromobacterium subtsugae* strain PRAA4-1. Both of these products are manufactured by Marrone Bio Innovations and distributed on the turf market by Prime Source, a division of Albaugh LLC. In years past, both products were evaluated separately for nematode control on golf course turf at the University of Florida with inconsistent results. However, in 2021 and 2022 field trials evaluated both products together, in combination and rotation, on ‘TifEagle’ bermudagrass with a heavy infestation of the grass root-knot nematode (*Meloidogyne graminis*). In 2021 the products were evaluated individually, and together in rotation or combination. Zelto, Crescendo, and their combinations were compared to each other and untreated controls. In 2022 the combination and rotation treatments of Zelto and Crescendo were compared to each other, untreated controls, and the industry standard nematicides fluopyram and abamectin. In both years, the combination of these two products greatly enhanced turf health and reduced the population density of *M. graminis*. These results indicate that the combination of Zelto and Crescendo provides excellent control of *M. graminis* on golf course bermudagrass. This provides a biologically-derived alternative to synthetic nematicides for management of *M. graminis* that is both safe and effective.

**TYMIRIUM™ Technology for Management of *Meloidogyne Graminis* and *Hoplolaimus Galeatus* on Golf Course Bermudagrass**

Crow, William T.^1^ and L. Dant^2^

^1^Entomology and Nematology Department, University of Florida, Gainesville, FL

^2^Syngenta Crop Protection, Greensboro, NC

## Abstract

TYMIRIUM technology (cyclobutrifluram) is a new SDHI nematicide discovered by Syngenta Crop Protection. It is currently being evaluated as a seed treatment and as a soil treatment on many crops worldwide. Studies evaluating efficacy of TYMIRIUM against nematodes infecting bermudagrass maintained as golf course putting greens have been conducted at the University of Florida for several years. The target nematodes in these trials were the grass root-knot nematode (*Meloidogyne graminis*) and lance nematode (*Hoplolaimus galeatus*). TYMIRIUM was tested on these nematodes in multiple field trials targeting each of the nematodes separately, and in some cases together. In these trials TYMIRIUM was compared to untreated controls and an industry standard (fluopyram). A reduction in the population density of *Meloidogyne graminis* was usually, but not always observed. A reduction in the population density of lance nematode was usually not observed. In every trial, regardless of the target nematode, TYMIRIUM consistently improved turf and root health compared to the untreated control. Additionally, TYMIRIUM generally increased turf and root health more than fluopyram. These results indicate that in the future TYMIRIUM may become a powerful tool for promoting turf health in nematode-infested golf course turf.

**Getting to the “Root” of Nematode Thresholds on Turfgrasses**

Crow, William T.^1^, A. Habteweld^1^ and S. J. Kammerer^2^

^1^Entomology and Nematology Dept., University of Florida

^2^United States Golf Association Green Section, Sharpsburg, GA

## Abstract

Nematode action thresholds and economic thresholds are very useful for making pre-planting management decisions on annual crops. However, for established perennial crops like turfgrasses they are much less accurate and are commonly misused. Without taking into account turf and root health the nematode counts lack context, and by themselves provide minimal information. Case studies from turfgrass field trials will illustrate the relationships between diagnostic counts of *Belonolaimus longicaudatus* and *Hoplolaimus galeatus*, and turf and root health. A new method for quantifying root efficiency that incorporates detailed observation of turfgrass roots along with nematode counts will be presented. Finally, proposed methods to rapidly combine root health measurements with nematode count data in a diagnostic setting will be discussed.

**Investigating the Impacts of Plant-Parasitic Nematode Communities on Michigan Carrot Production**

Darling, Elisabeth, S. Thapa, H. Chung, and M. Quintanilla-Tornel

Department of Entomology, Michigan State University, East Lansing, MI 48823

## Abstract

Michigan is the second most diverse agricultural state in the United States, producing over 300 commodities worth over $104.7 billion annually. Of this, $453 million annually can be attributed to vegetable crops, ranking within the top fourth nationally for asparagus, turnips, cucumbers, celery, green beans, pumpkins, radishes, summer and winter squash, sweet corn, potatoes, tomatoes, and carrots. Carrots (fresh-market and processing) are susceptible to damage and yield loss by many pests and pathogens, including plant-parasitic nematodes. Plant-parasitic nematode infestations can result in stubby, forked, or hairy carrots which renders the crop unmarketable. To determine the current impact of plant-parasitic and beneficial nematode communities in Michigan carrot farms, we conducted a state-wide survey encompassing the largest producers of fresh and processing carrot fields in the state (N=25). We identified *Pratylenchus* spp. as the top plant parasitic nematodes uncovered in Michigan processing soils, while the top plant-parasitic nematodes uncovered in Michigan fresh market carrots were *Heterodera carotae* and *Meloidogyne* spp. Additionally, we recorded harvest growth parameters, variety, field history, and soil type from each field to monitor any potential trends between fields. Following the survey, ten individual nematodes from each field were identified to species level by amplifying the D2A/D3B expansion segments of the 28S region of the rDNA gene for sequencing and species identification. Morphological and molecular data for each nematode sample was collected for all individuals to further understand the density and distribution of certain species and populations within Michigan carrot fields. Uncovering details of how various genera, species, and populations impact, penetrate, and thrive within carrot crops can provide nematologists with a better understanding of how to develop effective management strategies based on their risk to the industry in Michigan and beyond.

**A Bacillus Thuringiensis Cry Protein Controls Soybean Cyst Nematode in Transgenic Soybean Plants**

Daum, Julia, T. W. Kahn and M. T. McCarville

BASF Morrisville, NC 27709

## Abstract

Soybean cyst nematode (SCN) is a leading cause of soybean damage and yield loss in North America, resulting in an estimated $1.5 billion in economic loss to growers each year. The need for new solutions for controlling SCN is becoming increasingly urgent due to the slow decline in effectiveness of the widely used native soybean resistance source PI88788. Here we report a Bacillus thuringiensis delta-endotoxin, Cry14Ab, that controls SCN in transgenic soybean. This 130 kDa Cry toxin was discovered using an *in vitro C. elegans* 96-well bioassay and has an approximate EC50 of 7 ug/ml, which is similar to that of other *C. elegans*-active Cry toxins. The toxin damages the intestine, and fluorescently labeled Cry14Ab binds to the intestinal lining, suggesting the mechanism of action is similar to that of insect-active Cry proteins. Soybean plants expressing Cry14Ab and challenged with SCN show a significant reduction in the number of SCN cysts per plant compared to control plants in 30-day greenhouse assays. Field trials in SCN-infested soil also show a reduction in the SCN egg population density and an increase in soybean yield compared with control plants. These results demonstrate that the Cry14Ab protein has excellent potential to control SCN in commercial soybean. This new nematode resistance trait technology will provide a new tool to help growers protect their soybean crop and manage SCN populations.

**Host Suitability and Feeding Habit of *Aphelenchoides Pseudobesseyi* on Strawberry (*Fragaria Ananasa*)**

Demesyeux, Lynhe and W. T. Crow

University of Florida, Dept. of Entomology and Nematology, Gainesville, FL 32608

## Abstract

*Aphelenchoides pseudobesseyi* is a foliar nematode that was recently separated from the species complex of *A. besseyi*, a known parasite of strawberry. Apart from a thorough description of its morphological and molecular characteristics, little is known on the behavior and the ability of this new species to parasitize high value crops such a strawberry in Florida, USA. The objectives of this research were to investigate the host status of strawberry to *A. pseudobesseyi*, and to determine the feeding habit of this nematode on this crop under greenhouse conditions. Strawberry transplants were grown in 15 cm-diam. clay pots and were distributed in a Randomized Complete Block Design with 5 blocks in total. Each block included 5 inoculated and 5 non-inoculated plants. All leaves, stems and lateral runners were removed from the plants to promote the growth of new shoots. Once the new shoots reached 7 to 10 cm tall, the inoculated plants were inoculated with ~300 *A. pseudobesseyi* each and then allowed to grow for 90 days post inoculation. The plants were monitored for typical foliar nematode symptoms on the leaves, stems, flowers, and fruits. Additionally, flower number and fruits numbers were recorded for all the plants. At the end of the experiment, the number of *A. pseudobesseyi* extracted from the plant surface by surface washing, and from internally via reverse-Baermann extraction were recorded. Data were analyzed using R (version 4.1.3). Only 2 inoculated plants showed a few symptoms of foliar nematode infection, such as darker green leaf color and crinkled leaves. The final nematode count was variable, ranging from 1 to 557 per plant. The number recovered from internal extraction (mean ± SE = 105 ± 3) was more than one hundred time higher than from the external extraction (mean ± SE = 1 ± 2). Non-inoculated plants produced 3.5 times more flowers than inoculated plants, mean ± SE = 4.64 ± 0.45 and 1.32 ± 0.23, respectively. The same case scenario was registered for fruit production, where non-inoculated plants produced almost 4 times the number of fruits produced by inoculated ones, mean ± SE = 3.68 ± 0.63 and 0.37 ± 0.12, respectively. These results indicate that *A. pseudobesseyi* is primarily an endoparasite of strawberry, different from *A. besseyi* that is reported as an ectoparasite. Further, infection by *A. pseudobesseyi* caused yield loss under greenhouse conditions.

**Plant-Parasitic Nematodes Associated with *Cannabis Sativa* In Florida**

Desaeger, Johan^1^, J. Coburn^1^, J. Freeman^2^ and Z. Brym^3^

^1^Department of Entomology and Nematology, Gulf Coast Research and Education Center, University of Florida, Wimauma, FL 33598

^2^Horticultural Sciences Department, North Florida Research and Education Center, University of Florida, Quincy, FL 32351

^3^Department of Agronomy, Tropical Research and Education Center, University of Florida, Homestead, FL 33031

## Abstract

The subtropical climate of Florida allows for a wide range of crops to be grown. With the classification of hemp (*Cannabis sativa* L.) as an agricultural commodity, hemp has become a potential alternative crop in Florida. To support the future viability and sustainability of hemp and considering the importance of plant-parasitic nematodes (PPN) in Florida, it is critical to collect information on pest pressure of PPN on hemp in Florida. Hemp cultivars of different geographies (Europe, China and North America), and uses (fiber, oil and flower), were evaluated in seven greenhouse and three field experiments. Greenhouse experiments were conducted in sandy soil with 11 cultivars to explore host status and susceptibility to root-knot nematodes (*Meloidogyne* spp., RKN, five trials) and sting nematodes (*Belonolaimus longicaudatus*, SN, two trials). The field experiments evaluated a total of 26 cultivars and were done for two consecutive seasons at three different locations (soil types) in north (sandy loam), central (fine sand) and south Florida (gravelly loam). Nematode soil populations were measured at the end of each season. All 11 hemp cultivars were good hosts to root-knot nematodes (*M. javanica* and *M. incognita*, *M. enterolobii* and *M. hapla*), having reproduction rates like cucumber, with numerous root galls and sometimes reduced root size. No negative impact on hemp growth was seen, except in one trial where highly root-knot infested natural soil was used, and hemp biomass of a cannabigerol-rich (CBG) flower cultivar was significantly reduced. Hemp cultivars showed more differences regarding sting nematodes, with three fiber and one cannabidiol-rich (CBD) flower cultivars showing lower nematode reproduction as compared to strawberry. A diverse population of plant-parasitic nematodes was found in all three field sites, with reniform nematodes (RN, *Rotylenchulus reniformis*) the dominant species in north and south Florida, and RKN (*Meloidogne javanica*) the main species in central Florida. Other nematodes that were commonly found in South Florida (and to a lesser extent north Florida) were spiral (Helicotylenchus spp.), stunt (*Tylenchorhynchus* spp.) and ring nematodes (Criconemoids), while in Central Florida, stubby root (*Nanidorus minor*) and sting nematodes (*Belonolaimus longicaduatus*) were found. Reniform nematode populations were highest in south Florida (up to 27.5 nematodes/cc soil) and root-knot populations in central Florida (up to 4.7 nematodes/cc soil). No significant difference among cultivar host status or health was noted at any of the locations. This is the first report on plant-parasitic nematodes associated with hemp in Florida. RKN reproduced well on all cultivars, while SN showed more differences. Natural nematode populations varied greatly depending on where in Florida hemp was grown. RKN were found in all three regions and soils, while RN were only found in North and South Florida. Both RKN and RN populations were able to build up to high populations on most hemp cultivars. Growers that wish to include hemp in their crop rotation schemes need to be aware of potential pest pressure from nematodes. More research is needed to determine to what extent nematodes, especially RKN and RN can reduce hemp growth and yield.

**Lumialza™: A Novel, Biological Seed Treatment Nematicide Offering from Corteva Agriscience™**

Dickenson, Becky^1^, O. Garcia^2^, D. Rule^1^, J. Bugg^1^ and T. Thoden^3^

^1^Corteva Agriscience, Crop Protection Discovery and Development, IN

^2^Corteva Agriscience, Crop Protection Discovery and Development, São Paulo, Brazil

^3^Corteva Agriscience, Crop Protection Discovery and Development, Munich, Germany

## Abstract

Lumialza™ is a biological seed treatment nematicide based on a naturally occurring soil bacterium, *Bacillus amyloliquefaciens*, strain PTA-4838. This nematicide provides farmers with early season protection from plant-parasitic nematodes (PPN) by forming a living bio-barrier on roots. Lumialza has broad-spectrum activity against PPNs and has an extremely favorable safety profile. This low use rate seed treatment option is currently registered in the United States and Brazil, with plans to register across other geographies in the future. Lumialza attributes from controlled condition testing as well as field trial results from corn and soybean will be summarized in this poster.

**Leveraging Nematode Biology towards Improved Crops**

DiGennaro, Peter, M. Bogale, K. Hughes and S. Mishra

University of Florida, Dept. of Entomology and Nematology, Gainesville, FL 32611

## Abstract

Some of the most agriculturally devastating plant parasitic nematodes represent a unique and powerful tool to understand basal plant biology. Root-knot nematode (*Meloidogyne* spp.) initiates and maintains unique and intricate symbioses that is identical across its wide and diverse host range, implicating conserved pathways and mechanisms. Further, the availability of genomic and genetic resources allows makes this parasite an ideal biotic interrogator or plant systems. Several datasets that demonstrate the utility of this cosmopolitan pest with a focus on understanding the intersection of plant responses to biotic (nematode) and abiotic (increasing temperature) stresses will be discussed. Specifically, DNA variants between parental accessions of a tomato mapping population contribute to differences in important nematode infectivity traits and gene expression responses, and that the magnitude of these responses depends on the presence of the abiotic stressor (warmer nighttime temperatures). Further, gene expression levels of genetically homogeneous nematodes depend both on which host accession they infect and which temperature regimen the infected host experiences. The impacts of this work include the ability to identify plant lines that are more resilient, but importantly, also to elucidate the molecular biology behind the parasite response to those plants under abiotic stress. This work aims to identify relevant host genes and informs the mechanisms by which those genes alter the nematode biology. Understanding the nematode in addition to the plant paves the way towards targeting the parasite directly and responding to global changes in abiotic stressors.

**Evaluation of At-Plant Insecticides and Nematicides to Preserve Cotton Seedling Plant Health and Yield**

Douglas, Thomas J., S.H. Graham and K.S. Lawrence

Auburn University, Dept. of Entomology and Plant Pathology, Auburn, AL 36849

## Abstract

For optimal management, it is of paramount importance that early season cotton is protected from some key pests. In Alabama, two key plant parasitic nematodes, *Meloidogyne incognita* (root-knot nematode) and *Rotylenchulus reniformis* (reniform nematode), are important in various locations across the state. The tobacco thrips, *Franklinella fusca*, is the most important insect pest of seedling cotton throughout the state. Due to ease of application and relative efficacy, this early season pest spectrum is often managed using in-furrow applications or preventative seed treatments. The objective of this study was to evaluate the effects of early season treatment methods, such as in-furrow applications and seed treatments, on the health of seedling cotton. Field trials arranged as a RCBD with four or five replications were established in central (root-knot) and north (reniform) Alabama. At both locations, a nematode susceptible variety, DP 1646 B2XF was planted at a seeding rate of four seeds per foot. Treatments included: 1) Fungicide Only check 2) AgLogic (aldicarb) 5lb 3) Guacho IST + Velum (clothianidin + fluopyram) 4) Aeris IST (thiodicarb + imidacloprid). Regardless of location, cotton treated with an IST tended to have less thrips injury than the fungicide only control. Similarly, cotton treated with an IST tended to have better vigor and greater above and below ground biomass than the fungicide only control. There were no differences in nematode populations regardless of treatment at either location.

**Potential of Nematode Resistance and Enhanced Microbial Biodegradation of The Novel Nematicide Salibro™ (Reklemel™ Active)**

Dyer, David, A. Agi, A. Shirley, J. Temple, S. Tewari, T. Thoden and A. Verma

Corteva Agriscience, 9330 Zionsville Rd., Indianapolis, IN 46268

## Abstract

Reduced performance of nematicides can be a result of two primary pathways including increased nematode tolerance (resistance buildup) as well as enhanced microbial biodegradation. Both of which might occur after repeated applications. While nematode resistance has not been reported for current as well as traditional soil applied nematicides far more reports exist on the development of enhanced microbial degradation. Salibro™ (Reklemel™ active) is a novel chemical nematicide that is currently being developed by Corteva Agriscience for the control of many key plant-parasitic nematodes around the world. During the development of this novel nematicide, preliminary studies showed low risk for both, nematode resistance and enhanced microbial biodegradation. An overview of the current understanding of these attributes in regards to Reklemel™ will be presented.

**Soils Conducive to *Meloidogyne Hapla* in Washington State Vineyards**

East, Katherine^1^, D. Rippner^1^ and I. Zasada^2^

^1^USDA-ARS, Prosser, WA 99350

^2^USDA-ARS, Corvallis, OR 97331

## Abstract

The northern root-knot nematode *Meloidogyne hapla* is the major nematode pest of eastern Washington state wine grape (*Vitis vinifera*) vineyards. Soil population densities of *M. hapla* among vineyards in this region are highly variable and difficult to explain by grape variety or vineyard age alone. There is evidence that soil characteristics, including soil texture, impact the ability of second-stage juvenile (J2) *M. hapla* to find and infect vine roots. We conducted a survey of vineyard soils to investigate the influence of a range of soil characteristics on *M. hapla* parasitism. Forty-two vineyard and vineyard-adjacent soils from 12 American Viticultural Areas across Washington state and Oregon were collected. A subset of each soil was characterized across 31 components (including percent sand, silt, clay, pH, etc.) by a commercial lab. Soils were steam pasteurized, and one tomato (*Solanum lycopersicum* ‘Rutgers’) was planted into each soil in 10 replicate 0.5 L pots with moisture adjusted to 60% field capacity. *Meloidogyne hapla* J2, 500, were pipetted into 3 corners of each pot after planting and tomatoes were grown in the greenhouse for 7 weeks. After 7 weeks, plants were removed and *M. hapla* eggs were bleach extracted from tomato roots. Tomato roots were dried and weighed, and *M. hapla* egg densities were expressed per g root. Eggs were (log + 1) transformed to meet assumptions of variance. A principal component analysis was conducted to examine relationships between *M. hapla* egg densities and soil characteristics. Initial findings show that soil texture (% sand) and soil pH are most closely associated with greater *M. hapla* reproductive success. Further investigation with other analytic components will allow for better understanding of the relationships between soil characters and *M. hapla* parasitism of wine grapes.

**Workshop on the Creation of Captivating and Effective Presentations, Including Posters**

Eisenback, Jonathan D

School of Plant and Environmental Sciences, Virginia Tech, Blacksburg, VA

## Abstract

This workshop will demonstrate ways to make your presentations more captivating and effective. Starting at the beginning, we will show how to hook your audience and make them hear, understand, and remember the key points of your talk. We will take any presentation that you have recently given and provide you with valuable critique on how to make it better. From talks given at professional meetings that may influence future job offers to exciting lectures in business, academia, or extension that will help earn promotion or tenure, the value of a powerful presentation cannot be overestimated.

**Field Performance of Forty Maturity Group Iv and V Soybean Cultivars in a Southern Root-Knot Nematode Infested Field**

Emerson, Michael, J. Kelly and T.R. Faske

University of Arkansas, Dept. of Entomology and Plant Pathology, Lonoke, AR 72086

## Abstract

Forty soybean cultivars that were marketed as resistant to the southern root-knot nematode (*Meloidogyne incognita*) were evaluated in the field. In all trials, the damage threshold was severe with an average population density of 230 second-stage juveniles/100 cm^3^ of soil at harvest. Host susceptibility was based on the percent of root system galled at the R5 growth stage. Cultivars were considered very resistant if the percentage of root system galled was between 0.0 to 1.0%, resistant 1.1 to 4.0%, and moderately resistant 4.1 to 9.0%. Of the maturity group 4 cultivars, Pioneer 45A29L-SA2P was resistant, while Delta Grow DG4940, Progeny P4431E3, Armor EN21E42, Pioneer 46A35, Delta Grow DG46E10, Pioneer P43A42X, Armor EN21E49, Petrus Seed 49G16GT were moderately resistant. In the maturity group 5 trials, Pioneer P52A43L-SA2P was very resistant; Pioneer P52A05X and NK S55-Q3 were resistant; Pioneer P53A74BX, Pioneer P54A54X, Pioneer P55A49X, Progeny P5424XF, Syngenta NKS61-M2X, and Progeny P5554RX were moderately resistant. There was a significant negative correlation (*r* = -0.82, *P* = 0.0001) between galling and yield when averaged across maturity groups. Yield losses were significant and ranged between 52 to 65% for susceptible cultivars when compared to those with resistantance. These data indicate that some but not all soybean cultivars marketed as resistant are actually resistant to *M. incognita*, and useful cultivars options are available in both maturity groups. These resistant cultivars provide significant yield protection in fields with a high population density of *M. incognita* in silt loam soils in Arkansas.

**Multi-Year Assessment of Seed-Applied Nematicides Against *Meloidogyne Incognita* on Soybean in Arkansas**

Faske, T.R., M. Emerson and J. Kelly

University of Arkansas System, Division of Agriculture, Lonoke Extension Center, Lonoke, AR

## Abstract

A multi-year (2018 to 2021) field study was conducted to evaluate the field efficacy of eight seed-applied nematicides against *Meloidogyne incognita* on susceptible or partially resistant soybean cultivars. The final population density of the *M. incognita* average across trials ranged from 300 to 500 nematodes/100 cm^3^ soil, which can cause significant yield loss on a susceptible soybean cultivar. There was no year by nematicide interaction on susceptible or resistant cultivars for suppression of root-knot nematode galling or grain yield. Root galling was similar across all nematicide treatments on susceptible and partially resistant cultivars. No seed-applied nematicide significantly protected grain yield on either cultivar type. However, partially resistant cultivars had a greater grain yield average than susceptible cultivars. Of the nematicides, only fluopyram and *B. amyloliquefaciens* + cis-jasmone had a positive grain yield trend, an average of three bushel per acre on the susceptible soybean cultivar. All nematicides averaged less than two bushel per acre on the resistant cultivars. Fluopyram was profitable on the susceptible soybean cultivar, while *B. amyloliquefaciens* + cis-jasmone was profitable on the susceptible and partially resistant cultivars. These seed-applied nematicides provide little grain yield protection on susceptible and less on partially resistant cultivars in Arkansas.

**Synopsis on Nematode Infections and Disease in Alaska Fishes**

Ferguson, Jayde A

Alaska Department of Fish and Game, Division of Commercial Fisheries, Fish Pathology Section, Anchorage, AK

## Abstract

Nematodes comprise a significant group of helminths parasitizing fishes in Alaska. Review of case files from the Alaska Department of Fish & Game Fish Pathology Laboratory and the literature showed that some 16 or so species have been reported to parasitize freshwater or marine fishes in Alaskan waters. The most common and widespread infestations involved larval stages of the anisakids (*Anisakis*, *Contracaecum*, *Pseudoterranova*) infecting most families of marine and anadromous fishes worldwide. These were frequently encountered in Pacific salmon (*Oncorhynchus* species), Pacific cod (*Gadus macrocephalus*), walleye pollock (*Gadus chalcogrammus*), Pacific halibut (*Hippoglossus stenolepis*) and Pacific herring (*Clupea pallasi*i) in Alaska. The larval infestations are generally well tolerated by fish with minimal associated pathology. However, they are zoonotic where humans are dead-end hosts resulting in death of the worms and ensuing intense inflammation that can be life-threatening if the larval migrations and encystments involve vital organs, such as heart and brain. There were records in the Alaska database of adult *Raphidascaris* parasitizing sockeye salmon (*Oncorhynchus nerka*) and least cisco (*Coregonus sardinella*) and the literature had additional reports in other salmonid host species as well as northern pike (*Esox Lucius*). One unique unpublished report existed in the database of an undescribed adult *Hysterothylacium* species parasitizing three-spined stickleback (*Gasterosteus aculeatus*). The next most common nematode infestations in Alaska involved the philometrids (dracunculids) including *Philometra* and *Philonema* that mature in salmonids. Maturation of the latter genus is in synchrony with spawning of adult fish and heavy worm burdens can induce notable host tissue pathology of visceral adhesions and fibrogranulomatous (pseudomembranous) peritonitis. Less frequent records involved adult specimens from the genera *Cystidicola* and *Cucullanus*/*Truttaedacnitis*, as well as the whipworms, *Pseudocapillaria*/*Capillaria*, in salmonids. Heavy parasitism of *Cystidicola* in the swimbladder of Alaskan salmonids could affect fish buoyancy resulting in excessive energetic demand that may impact fish survival. The literature suggests that *Cucullanus*/ *Truttaedacnitis* is associated with reduced fish growth. The pathogenicity of *Pseudocapillaria*/*Capillaria* in Alaskan salmonids is unknown. However, the worm has potential to reduce host survival based on reports in the literature of mortality in cyprinids associated with *Pseudocapillaria tomentosa*. Overall, the nematode fauna of Alaskan fishes has not been associated with any known population declines.

**Establishment and Growth of Ring Nematode Populations under Sweet Cherry Trees as Affected by Soil Management Practices**

Forge, Thomas^1^, P. Munro^1^ and T. Watson^2^

^1^Agriculture and Agri-Food Canada, Summerland Research and Development Centre, 4200 Highway 97, Summerland, BC V0H 1Z0, Canada

^2^Department of Plant Pathology and Crop Physiology, Louisiana State University, Baton Rouge, LA

## Abstract

The ring nematode, *Mesocriconema xenoplax*, is widespread but at variable population densities in mature sweet cherry orchards in the Okanagan Valley of British Columbia. Little is known, however, of the dynamics of population establishment and growth in newly planted cherry orchards or how orchard management practices might influence population growth in newly planted orchards. Here we describe natural establishment and growth of a *M. xenoplax* population over nine years after planting a new field experiment. The site was previously an apple orchard and *M. xenoplax* was not detected in soil or roots of the nursery stock prior to planting. In April 2014, four cherry trees (‘Skeena’ cultivar on Gisela 6 rootstock) from a certified nursery were planted into each of sixty 5m x 3m plots. The plots were arranged in a two-factor split-plot experimental design to assess interactive effects of irrigation systems (drip vs micro-sprinkler) and soil treatments (non-treated control, fumigation, compost, bark mulch, compost+mulch,) on early tree growth. By fall of 2015, *M. xenoplax* was present in only 3% of plots but by fall of 2020 and 2021 it was present in 53% and 55% of the plots, respectively. The overall average population density (including non-infested plots) was 52 *M. xenoplax*/100 cm^3^ soil, with a maximum of 965 *M. xenoplax*/100 cm^3^ soil. The multi-year *M. xenoplax* population data were converted to Area Under Population Curve (AUPC) by plot for further analyses. Treatments affected the likelihood of plots becoming infested by year (chi-square *p* < 0.001) and the level of population growth once they were infested. AUPC was greater under drip irrigation than micro-sprinkler (*p* = 0.016), and in compost treated soil than in mulched and non-treated soil (*p* < 0.05). Among compost treated plots there was a significant negative relationship between *M. xenoplax* AUPC and tree cross sectional area (*p* = 0.006). Our data describe the development of a natural *M. xenoplax* infestation in a new cherry orchard. They also demonstrate that irrigation and soil management can influence infestation development, and that subsequent effects of the nematode on tree growth may vary with soil management.

***Meloidogyne Enterolobii*: A South African Perspective**

Fourie, Hendrika^1^, M. Daneel^1,2^ and M. Marais^3^

^1^Unit for Environmental Sciences and Management, North-West University, Private Bag X6001, Potchefstroom 2520, South Africa

^2^Agricultural Research Council – Tropical and Subtropical Crops (ARC-TSC), Private Bag X11208, Mbombela 1200, South Africa

^3^Nematology Unit, Biosystematics, Agricultural Research Council – Plant Health and Protection (ARC-PHP), Private Bag X134, Queenswood 0121, South Africa

## Abstract

*Meloidogyne arenaria*, *M. hapla*, *M. incognita* and *M. javanica* are generally considered the economically most important root-knot nematode species globally, with *M. enterolobii* being worthy to be added to the list. During the last decade, this emerging threat species has been increasingly recorded from countries across the globe mainly due to the use of molecular techniques to accurately identify it. Although it is an A1 quarantine organism in Europe and various other countries, it does not apply for South Africa (SA) where it was first reported in 1997 (from guava roots). No comprehensive molecular and/or morphological knowledge existed for *M. enterolobii* populations from SA, resulting in an extensive study being conducted during which samples were obtained from local crop production areas; root-knot nematode species were identified using both morphological and morphometrical techniques. Information about the genetic diversity of species from the *Mi*-group, e.g., *M. enterolobi*i, *M. incognita* and *M. javanica*, were furthermore obtained using genotyping by sequencing (GBS), while knowledge on the life-stage development and life cycle duration, host plants and reproduction potential of such populations were also acquired. Morphological identification revealed the presence of large phasmids (surrounded by fine striae), fine striae on lateral sides of the vulva and the presence of atypical perineal-patterns (medium to high square-like dorsal arches) in perineal-pattern areas of *M. enterolobii* females, allowing differentiation of the species from its thermophilic counterparts. Molecular techniques verified the presence of *M. enterolobii* populations and 653 common single nucleotide polymorphisms (SNPs) were identified with principal component and phylogenetic analyses placing all populations of *M. enterolobii* in one clade, *M. javanica* in another and *M. incognita* in an intermediate clade. Alleles present only in the genome of *M. enterolobii* and located in genes involved in virulence in other animal species were identified too. Life cycle studies showed that *M. enterolobii* females produced single eggs from 15 days after inoculation of second-stage juveniles in roots of maize, soybean and tomato, while *M. incognita* and *M. javanica* produced egg masses from 20 DAI; suggesting a shorter life cycle for *M. enterolobii*. First reports of host plants added to the list for *M. enterolobii* were expanded to dry bean, eggplant, groundnut, lettuce, maize, soybean and spinach opposed to the known hosts (tomato, peppers and potato). In addition, its presence was expanded from three to five provinces, while the pathogenicity of *M. enterolobii* among populations showed substantial differences using reproduction factors. Recent research shed light on crucial aspects of South African *M. enterolobii* populations compared to its thermophilic counterparts. Such novel knowledge is crucial for knowing where the species is present and ultimately the management of *Meloidogyne* spp.

**The Impacts of Conservation and Regenerative Agriculture on Root and Soil Ecosystem Health using Nematodes**

Fourie, Hendrika, G. du Preez and A. Loggenberg

Unit for Environmental Sciences and Management, North-West University, Potchefstroom, South Africa

## Abstract

Conservation (CA) and regenerative agriculture (RA) are endorsed as systems that promote environmental health, agroecosystem resilience, and sustainable food production. Healthy and functioning soil ecosystems are key aspects of these two approaches. Information about the potential of soil ecological restoration after transitioning from conventional agriculture (CT) systems to either CA or RA is, however, limited especially in the African context. The focus of this study was hence on soil ecosystem health and functioning in CA and RA-based crop production systems in multiple croplands situated in two provinces of South Africa. Replicated root and soil samples were collected during the summer and winter growing seasons since 2019 from CA and RA croplands, as well as from a CT cropland (negative reference) and undisturbed grassland (positive reference). Nematode community data were used to calculate nematode-based indices, while active carbon, soil respiration, total organic matter, and physico-chemical parameters were also measured. Results for RA croplands showed significant ecological restoration and increased ecosystem functioning with structured food webs and fertile soils. According to redundancy analyses, the observed effects mainly resulted from the change in systems, i.e., from CT to RA. Results from CA croplands did not necessarily provided evidence that ecological restoration took place and substantial increases in densities of plant-parasitic nematodes were recorded for root samples. Undisturbed grassland consistently presented mature and structured food webs, supporting their use as positive reference sites for agroecosystem restoration. Correlations between physico-chemical parameters and nematode data represented a linear relationship between inorganic nitrogen and soil food web structure (R^2^=0.51; p<0.0001), and between copper and soil respiration (microbial activity) (R^2^=0.6; p<0.0001) for the RA croplands. This indicates the potential negative effects of agrochemicals on soil ecological restoration. Results confirmed that RA can restore the health and functioning of soil ecosystems and ultimately promote service delivery, while evidence for CA is not as clear.

**Novel Diagnostic Markers to Detect and Assess *M. Chitwoodi* Race 1, Race 2 and Roza Presence within Washington State**

Franco, Jessica^1^, S. Hu^2^, S. Akinbade^3^, V. Sathuvalli^2^ and C. Gleason^1^

^1^Plant Pathology Department, Washington State University, Pullman, WA 99164

^2^Hermiston Agricultural Research and Extension Center, Oregon State University, Hermiston, OR 97838

^3^Washington State Department of Agriculture, Prosser, WA 99350

## Abstract

The Columbia root-knot nematode, *Meloidogyne chitwoodi*, is a potato pest within the Pacific Northwest. It poses a greater threat to the potato industry than the other *Meloidogyne* species present within the area because of its wider host range, adaptation to cooler temperatures, and infected potato tubers possess more severe internal and external defects. Crop rotations and nematicide applications have been used to control *M. chitwoodi* populations. However, *M. chitwoodi* has a wide host range providing significant challenges for its management. Additionally, nematicides are costly and toxic to the environment and human health. *M. chitwoodi* was first identified in the region the early 1980s. Since then, populations have emerged with different host ranges. The first identified population, Race 1, can infect carrot (“Chantenay”) but not alfalfa (“Thor”). Alfalfa was used as a rotation crop to reduce *M. chitwoodi* populations. After five years, a novel *M. chitwoodi* population was able to infect alfalfa but not carrot. This isolate has been designated Race 2. Resistance against Race 1 and Race 2 was identified and introgressed from the wild potato species, *S. bulbocastanum*, to generate a resistant breeding clone. The resistance offered by this clone was overcome by the *M. chitwoodi* pathotype, Roza. Roza, like Race 1, can infect carrot and not alfalfa. Race 1, Race 2, and Roza are morphologically identical and can be differentiated by their host ranges or pathogenicity on the resistant breeding clones. Host tests are time consuming therefore methods to quickly detect these isolates are needed. We have taken a DNA-based approach to generate markers against the *M. chitwoodi* isolates. Comparative genome analyses between Race 1, Race 2, and Roza genomes revealed regions that display the most genetic diversity. A total of 220 primer sets were generated and tested. We have successfully identified race and pathotype specific markers that can amplify in both pure DNA and in more complex soil samples. We have tested over 50 field sites within the Washington side of the Columbia Basin using our isolate-specific markers and the previously published *M. chitwoodi* marker, JMV1/JMV2. Race 1 and Roza were detected but not Race 2. Between Race 1 and Roza, Roza is the most prevalent *M. chitwoodi* isolate thus far. Co-infection with Race 1 and Roza was observed within a subset of the tested field sites. Interestingly, in some fields, *M. chitwoodi* was detected using JMV1/ JVM2 but we were not able to resolve it to the isolate level. We are currently working on developing a multiplex reaction to detect all three isolates within a single reaction. Robust and sensitive diagnostic markers are urgently needed to guide management strategies to reduce losses and improve potato production.

**Biological Activity of TYMIRIUMTM Technology as a Soil- and Seed-Applied Nematicide**

Gaberthuee^l^, Matthias^1^, S. Morsello^2^, J. Hamill^2^, D. Ireland^2^, J. Simmons^3^ and A. Bachiega^4^

^1^Syngenta Crop Protection AG, Rosentalstrasse 67, 4002 Basel, Switzerland

^2^Syngenta Crop Protection LLC, 410 Swing Rd, Greensboro, NC 27409

^3^Syngenta Crop Protection LLC, VBRC, 7145 58th Avenue, Vero Beach, FL 32967

^4^Syngenta Proteção de Cultivos Ltda, Av. das Nações Unidas, 18001, Santo Amaro, São Paulo, Brazil

## Abstract

Plant-parasitic nematodes are hard to eradicate once a field site has been infested. The management of nematodes is very challenging and practices like crop rotation and cultural measures are difficult for growers to implement. A growing world population and a limited production area coupled with a demand for higher yields in existing production areas has led to an increased intensification which favours nematode multiplication. An efficient way to control nematodes is to apply a nematicide prior to or at sowing/transplanting. This is one of the key elements to protect the root development of plants in an early growing phase. A chemical nematicide can actively protect the young seedling in the first critical weeks of early establishment and secure crop yield. The newest active substance with outstanding nematicidal efficacy is TYMIRIUM^TM^ technology. TYMIRIUM^TM^ technology is highly active against a broad range of plant-parasitic nematodes and can be applied either as soil- or a seed treatment. During the presentation we will cover the activity of TYMIRIUM^TM^ technology in key crops against different genera of plant-parasitic nematodes and the benefit this new chemical nematicide can bring to growers.

**Deadly Scents: Exposure to Plant Volatiles Increases Mortality of Entomopathogenic Nematodes During Infection of *Galleria Mellonella***

Gaffke, Alexander^1,2^, H. Alborn^1^ and D. Shapiro-Ilan^3^

^1^Center for Medical, Agricultural, and Veterinary Entomology, Agricultural Research Service, United States Department of Agriculture, Gainesville, FL

^2^Department of Entomology, Louisiana State University, Baton Rouge, LA

^3^Southeastern Fruit and Tree Nut Research Station , Agricultural Research Service, United States Department of Agriculture, Byron, GA

## Abstract

Plants attacked by insects commonly mobilize various defense mechanisms, including the biosynthesis and release of so-called herbivore-induced plant volatiles (HIPVs). Entomopathogenic nematodes (EPNs) can be attracted to these belowground HIPVs, which can enhance biocontrol services from EPNs. However, recent research has also demonstrated that HIPVs can induce and initiate insect immune responses, decreasing the insect’s susceptibility to pathogens and parasites. Therefore, experiments were conducted to test the impact of HIPVs on insects and EPNs during the initial stage of EPN infection. Compounds that can impact EPN attraction and infectivity such as pregeijerene, β-caryophyllene, and α-pinene, and compounds that have been determined to increase or decrease susceptibility of insects to pathogens, such as (*Z*)-3-hexenyl acetate, linalool, and β-ocimene, were selected. Exposure *of Galleria mellonella* larvae to pregeijerene, linalool, β-ocimene and α-pinene during invasion significantly increased mortality of *Steinernema diaprepesi* and *Heterorhabditis bacteriophora* after 48 hrs. Larval treatment with β-caryophyllene only increased mortality for *Heterorhabditis bacteriophora*. (Z)-3-hexenyl acetate did not cause differential mortality from the controls for either nematode species. In additional experiments, we found that EPNs exposed to α-pinene and linalool were more readily recognized by the insects’ immune cells compared to the control treatment, thus the observed increased mortality was likely due to HIPVs-EPN interactions with the insect’s immune system. These results show that the presence of HIPVs can impact EPN survival in the model host, *G. mellonella*.

**Cover Crops for *Meloidogyne Hapla* Management in Washington Vineyards**

Gagnier, Bernadette^1^, I. Zasada^2^, M. Mireles^1^ and M. Moyer^1^

^1^WSU IAREC, Prosser, WA 99350

^2^USDA-ARS HCRU, Corvallis, OR 97331

## Abstract

The northern root-knot nematode (*Meloidogyne hapla*) is the most prevalent plant-parasitic nematode affecting Washington State *Vitis vinifera* (European winegrape) vineyards. This nematode induces small galls on roots, restricting water and nutrient uptake. In new vineyards this can impede establishment. In existing vineyards, it can exacerbate decline in chronically stressed vines. While preplant fumigation is a common strategy for *M. hapla* management, its efficacy is temporary. Pre- and post-plant cover crops are potentially viable additions to an integrated management approach for this grape-nematode system. Two trials were established evaluating cover crops to reduce *M. hapla* densities in in fallow and established vineyard sites. Litchi tomato (*Solanum sisymbriifolium*) was used as a pre-plant fumigation alternative in a vineyard replant site in Mattawa, WA. After one growing season (seeded on May 27, 2020), litchi tomato reduced existing *M. hapla* densities by 75% relative to a fallow control (fall 2020; p < 0.0002). This effect continued in the following spring with a 65% reduction relative to a fallow control (spring 2021; p < 0.0002). In Fall 2021, plots that were planted to litchi tomato for two years had *M. hapla* populations densities reduced by 84% compared to the fallow control (fall 2021; p = 0.014). To evaluate potential post-plant cover crop options for *M. hapla* suppression, a trial was established in 2020 evaluating ‘Dracula’ oilseed radish (*Raphanus sativus var. oleiformis*), ‘White Dutch’ clover (*Trifolium repens*) and ‘Pacific Gold’ mustard (*Brassica juncea*). After 1 year of cover crop establishment Dracula oilseed radish had 81% fewer *M. hapla* relative to the bare ground control (fall 2021; p = 0.048). While not statistically supported (p = 0.078), *M. hapla* population densities trended lower in plots planted with White Dutch clover and Pacific Gold mustard compared to control plots. Combined, our results demonstrate that pre- and post-plant cover crops may be viable options in the integrated management of *M. hapla* wine grape vineyards.

**Variation in Root Architectural Response of Three Sweetpotato Genotypes to Parasitism by *Meloidogyne Enterolobii* and *M. Incognita***

Galo, David^1^, A. Villordon^2^, D. Labonte^3^, C. Clark^1^ and T. Watson^1^

^1^Department of Plant Pathology and Crop Physiology, Louisiana State University Agricultural Center, Baton Rouge, LA 70803

^2^Sweet Potato Research Station, Louisiana State University Agricultural Center, Chase, LA 71324

^3^School of Plant, Environmental, and Soil Sciences, Louisiana State University Agricultural Center, Baton Rouge, LA 70803

## Abstract

Root architecture response of three sweetpotato genotypes infected with *Meloidogyne enterolobii* (*Me*) and *M. incognita* (*Mi*) was evaluated under greenhouse conditions. The sweetpotato genotypes ‘Beauregard’ (susceptible to *Me* and *Mi*), ‘Jewel’ (resistant to *Me* and tolerant to *Mi*), and LSU AgCenter breeding line ‘14-31’ (resistant to *Me* and *Mi*) were examined. Sweetpotato vine cuttings were transplanted into PVC pots filled with pasteurized sand, and inoculated with approximately 3,000, 500, or no eggs of either *Me* or *Mi* added immediately after planting. Plants were watered every other day alternating between deionized water and half-strength Hoagland’s solution. Twenty-one days after planting, entire root systems were washed free of sand, floated on waterproof transparent trays, scanned using a dual lens optical scanner, and analyzed using the software RhizoVision Explorer. Root architecture (RA) attributes such as lateral root (LR) length, surface area, volume, and number of root tips per plant were evaluated. Root galls were visually counted each time an image was collected. Statistical analyses were conducted using analysis of variance and means were separated using the LSD test. The only genotype that showed gall development was ‘Beauregard” using either inoculation level or nematode species. There were inherent genotype effects observed in root surface area (P<0.001) and volume (P<0.001), with the resistant genotype ‘14-31’ having a greater root surface area and volume than the other two genotypes, regardless of the level of nematode inoculum. However, ‘Beauregard’ inoculated with 500 eggs and ‘Jewel’ inoculated with 3000 eggs of either *Me* or *Mi* showed a significant increase in root surface area when compared to the respective non-inoculated controls. For LR length, results showed that ‘Jewel’ plants inoculated with 3000 and 500 eggs of *Me* or *Mi* have greater root length than the non-inoculated control (P<0.05). Also, a constant Genotype by Nematode interaction effect was observed for LR length, surface area, and volume. Although ‘Jewel’ plants inoculated with nematodes are the only treatments showing an increase of RA attributes, there is a positive correlation between *Me* or *Mi* inoculum level and the increase in RA attributes on the genotype ‘Beauregard’ and the breeding line ‘14-31’. Moreover, nematode inoculated plants had increased root length when compared to the non-inoculated controls within each genotype, and plants inoculated with *Me* showed a greater increase in RA attributes when compared to plants inoculated with *Mi*. In conclusion, this study suggest that compensatory root growth may be a contributing factor to nematode resistance in these sweetpotato genotypes.

**Evaluation of the Effect of Winter Cover Crops on Population Density of *Meloidogyne Incognita* under Field Conditions**

Gitonga, Denis and A. Hajihassani

University of Florida, Dept. of Entomology and Nematology, Fort Lauderdale Research and Education Center, Davie, FL 33314

## Abstract

Cover crops have been widely used for soil conservation and plant-parasitic nematode management. Two independent trials were conducted in winter 2021 and 2022 in a field naturally infested with the root-knot nematode (RKN; *Meloidogyne incognita*) to determine the effect of different termination timing of cover crops on nematode development in South Georgia, USA. The study utilized a randomized complete block design with five treatments [oilseed radish (cv. Carwoodi), oat (Tachibuki), rye (Wrens Abruzzi), mustard (Caliente), and rye-oat mixture]. Two fallows (with and without weeds) were included as controls. Each plot was 5 ft wide and 25 ft long with 6 ft between them. The cover crops were planted in early October and terminated 90 and 120 days after planting. Cover crop residues were then incorporated into the soil by a moldboard plow to achieve a maximum volume of allelopathic and/or nematicidal activity. RKN population densities were examined before planting, at termination, and two weeks after incorporating the crop residue into the soil. Biomass accumulation was determined using 10 random representative plants at the termination of cover crops. Oven-dried aboveground parts of plants were weighed, and their root systems were assessed for RKN gall severity. The RKN abundance differed (*P* < 0.01) between the two years (2021 and 2022). In 2021, there was an increase in the nematode population (*P* < 0.05) when the cover crops were left longer (120 days) in the field compared to a shorter period (90 days). The incorporation of cover crops in the soil was not significant in terms of RKN abundance in both trials. In 2021, rye-oat had the lowest RKN abundance (*P* < 0.01) compared to other treatments, while radish had the highest abundance (*P* < 0.01). There were no significant differences in the nematode density between rye, mustard, and the two fallows. In 2022, RKN abundance was not significantly affected by either the termination period (90 and 120 days) or cover crop incorporation in the soil. However, there were differences among the treatments with the fallow with weeds having the highest RKN abundance (*P* < 0.01) compared to other treatments. Oat, rye, and fallow without weeds had the lowest RKN abundance. The cover crop biomass was significantly higher in 2021 than in 2022, with radish having the highest biomass. Mustard had a higher galling index (*P* < 0.01) than other cover crops in both 90 and 120 termination days, while oat, rye and rye-oat mixture had the lowest galling index. In conclusion, rye and rye-oat mixture can reduce *M. incognita* populations in the soil. Additionally, leaving cover crops in the field for a longer period can lead to nematode population build-up that would negatively impact consecutive cash crops.

**Aliphatic Glucosinolates Enhance Plant Defense to the Root-Knot Nematode *Meloidogyne Incognita***

Godinez-Vidal, Damaris and S. C. Groen

University of California, Dept. of Nematology, Riverside, CA 92521

## Abstract

Root-knot nematodes (*Meloidogyne* spp.) are among the most critical pests of economically important horticultural crops causing significant yield losses. *M. incognita* (Kofoid et White) Chitw. is one of the four most common species in this genus of plant-parasitic nematodes that affect numerous hosts worldwide. Chemical control has been efficient in fighting plant-parasitic nematodes but has a negative environmental impact; thus, it is necessary to find a sustainable ecological alternative. Glucosinolates, a group of natural products found in the genetic model plant *Arabidopsis thaliana* and other members of the Brassicaceae family, have been shown to form an option for controlling pests and pathogens. Profiles of glucosinolates and their most potent derivatives, the isothiocyanates, differ among and within plant species and their toxicity against nematodes. Biosynthesis of the most diverse class of glucosinolates, the methionine-derived aliphatic glucosinolates, in *A. thaliana* is regulated by transcription factors MYB28 and MYB29; both are partially redundant, but synthesis of all aliphatic glucosinolates is blocked in the absence of both. Plants of a *myb28 knock-out* mutant no longer produce aliphatic glucosinolates with long side chains (6 carbons or more), but they still produce short-chain aliphatic glucosinolates. Most studies on glucosinolates have been carried out on leaves, where glucosinolates profiles are predominantly made up of short-chain; however, long-chain aliphatic glucosinolates accumulate at higher levels in the roots, which suggests they might play a role in defense against soil pathogens such as plant-parasitic nematodes. Considering this, we evaluated the infection of second-stage juveniles (J2) of *M. incognita* in *myb28* mutant and Col-0 wild-type roots. Two-weeks-old seedlings were inoculated with 200 J2 of *M. incognita*, and at 24, 48, and 72 h post-inoculation (hpi), root architecture was evaluated, and roots were stained with acid fuchsin. We observed no differences in root development between *myb28* and wild-type seedlings, but we found significant differences in the infection of *myb28* compared with wild-type roots at 48 and 72 hpi, suggesting that the absence of long-chain aliphatic glucosinolates could promote nematode infection. To confirm the results observed *in planta*, we assayed *in vitro* the biocidal activity of isothiocyanates derived from three aliphatic glucosinolates on J2 of *M. incognita* as expressed by their nematicidal and immobilization effects after 24 h. Isothiocyanates derived from the short-chain aliphatic glucosinolate glucoraphanin (4MSOB), and the long-chain aliphatic glucosinolates

(*Continued*)

## Abstract (Continued)

glucohesperin (6MSOH) and glucohirsutin (8MSOO) were prepared at 0.05 and 0.5 μmol concentrations. After 24 h of exposure in the dark at 21oC+1, the J2 were rinsed, the isothiocyanate solutions were removed, and distilled water was added. After 24 h, J2 treated with the different isothiocyanates showed immobilization without recovery. Significant differences between isothiocyanates derived from short- and long-chain glucosinolates were found, with J2 showing higher mortality after exposures to 6MSOH and 8MSOO isothiocyanate with the lower concentration assayed. These findings suggest that long-chain aliphatic glucosinolates and their isothiocyanate derivatives have relatively potent effects on plant defense against the root-knot nematode *M. incognita*, highlighting the potential relevance of these compounds for plant-parasitic nematode management.

**Development of a Recombinase Polymerase Amplification Assay for Rapid Detection of the Stubby Root Nematode, *Paratrichodorus Allius***

Goraya, Mankanwal and G. Yan

Department of Plant Pathology, North Dakota State University, Fargo, ND 58108

## Abstract

The stubby root nematode, *Paratrichodorus allius*, is an economically important ectoparasitic nematode that limits the profitability of potato industry by feeding on plant roots and transmitting *Tobacco rattle virus*, the causal agent of corky ringspot disease in potatoes. Successful stubby root nematode management relies on fast and precise detection and identification of the species in fields. Molecular techniques such as conventional PCR and quantitative real-time PCR (qPCR) are available for the diagnosis of *P. allius*. In this study, a more efficient recombinase polymerase amplification (RPA) assay was developed. This method requires minimal sample preparation, low temperature range, and 20-40 minutes for DNA amplification. Four pair of primers applicable to RPA reaction targeting internal transcribed spacer-ribosomal DNA (ITS-rDNA) of *P. allius* were designed. RPA was carried out with DNA from single nematodes in the extraction buffer using TwistDx Basic kit, and all the primers were evaluated at various temperatures (37°C, 39°C, 40°C, and 42°C) and different time durations (20, 30, and 40 minutes). The results were visualized using agarose gel electrophoresis. The primer pair, MG2F/MG2R producing amplicon length 326 bp was the best to detect genomic DNA of *P. allius* at 40°C within 20 minutes. *In-silico* analysis was performed to evaluate the specificity of template-primer bond using 60 species of stubby root nematodes. The specificity of RPA assay was tested using different plant-parasitic and free-living nematodes found in potato fields in North Dakota, Washington, and Idaho, and results showed single amplicons specific to *P. allius* only. The sensitivity of the assay was measured with serially diluted DNA samples from a single nematode. The gel electrophoresis-based RPA assay was able to detect *P. allius* equivalent to ½ nematode. The RPA product was verified using the published qPCR assay for *P. allius*. The RPA primers were further tested with the real-time fluorescence detection method using TwistDx exo kit. A FAM-labeled hybridization DNA probe, showing homology with the amplicon of MG2F/MG2R primers and specificity with the target species, was designed. The results were observed in a portable real time- fluorescence reader. A threshold level for *P. allius* identification was set up in the fluorescence reader. The real-time RPA can detect *P. allius*, equivalent to ¼ nematode. To assess the RPA’s ability detecting DNA from the soil, autoclaved soil was inoculated with different number of stubby root nematodes, DNA was extracted directly from the soil, and RPA was performed. The sensitivity and specificity of the assay to detect *P. allius* in artificially inoculated soil

(*Continued*)

## Abstract (Continued)

were found to be similar with detecting *P. allius* using DNA extracted directly from nematode materials. This method will be evaluated for detection of *P. allius* from naturally infested field soils. The RPA assay has the potential for rapid field-based and point-of-care applications to efficiently detect the stubby root nematode species for effective nematode management.

**Evaluation of Cereal and Broadleaf Cover Crops in a Greenhouse Bioassay for Host Status and Suppression of *Meloidogyne Enterolobii* in Soybean**

Gorny, Adrienne, N. Saha, T. Schwarz and P. B. Jeffreys

Department of Entomology and Plant Pathology, North Carolina State University, Raleigh, NC 27695

## Abstract

North Carolina is an agriculturally diverse landscape, with nearly 90 different crops and commodities grown across the state. *Meloidogyne enterolobii* is a newly introduced species of root-knot nematode in North Carolina. The species has a broad host range, potentially threatening production of many of the vegetable and agronomic crops grown in the state. In crops such as soybean, corn, or cotton, the use of expensive nematicides is cost prohibitive. Instead, short-term cultural management tactics for *M. enterolobii* are needed to manage the pathogen as long-term solutions (i.e. host resistance) are developed. The use of cover crops is popular in North Carolina, as they provide the benefits of improving soil structure, addition of nutrients and organic matter, and in some cases, provide an additional harvested crop or grazing opportunities. Crop covers may also provide a non-chemical alternative to soilborne disease management through being a non-host or antagonist to plant-parasitic nematodes. A greenhouse bioassay was performed to evaluate the host status of several popular cover crops and to quantify their effectiveness in reducing *M. enterolobii* pathogen pressure in a following soybean crop. Eight cover crops (three broadleaf and five cereal species) were seeded in pots in the greenhouse and inoculated with 10,000 eggs of a North Carolina isolate of *M. enterolobii*, three weeks after seeding. At approximately 80 days post seeding, cover crop plants were destructively harvested and roots were evaluated for the presence of galling. From each pot, 200 g of soil was removed and transferred to a new pot and amended with fresh soil. Into these pots, three soybean seeds were sown and plants maintained for 60 days post emergence. Soybean plants were then destructively harvested and data collected on *M. enterolobii* infection and reproduction. The trial was performed twice and data analyzed separately. Of the cover crops tested, none of the cereal cover crop species were host to *M. enterolobii* and resulted in significantly reduced root galling symptoms and nematode reproduction in the following soybean crop. All of the broadleaf cover crops included in the study (Yellow Mustard, Hairy Vetch, and Crimson Clover) were host to *M. enterolobii* and resulted in significantly more severe root galling and increased nematode reproduction in the following soybean plants. There were no significant differences in soybean shoot weight or soybean root weight in the first trial; however, significant differences of these parameters were present in the second trial. These data provide preliminary support for management recommendations for local farmers and inform selection of cover crop species for further investigation in field-based studies.

***Meloidogyne Arenaria* Management in Peanut Using Nematicides and Host Resistance**

Grabau, Zane J. and C. Liu

University of Florida, Entomology and Nematology Department, Gainesville, FL 32611

## Abstract

Peanut is an important crop in the United States with over 640,000 hectares planted in 2021 and harvested peanuts worth $1.5 billion. Much of this peanut production occurs in the Southeast where *Meloidogyne arenaria* (peanut root knot nematode, PRKN) ranks among the major pests and pathogens of peanut. Crop rotation, nematicide application, and use of resistant cultivars are the main practices growers employ to manage PRKN. In particular, choice of a particular nematicide or cultivar are the main short-term choices for PRKN once a grower has decided to plant a field to peanut. Both relatively new nematicides (fluopyram) and resistant cultivars (TifNV High O/L) as well as older nematicide chemistries (oxamyl and aldicarb) are available. The objective of this research was to evaluate efficacy of nematicides and host resistance for PRKN management in peanut production. To test this objective, a series of small plot field trials were completed at research centers in central and north-central Florida. Soil and root populations of PRKN, root galling, and peanut yield were assessed to determine efficacy of nematicides and host resistance.

**Overexpression 0f the Soybean α-Snap in Rhg1-a Type Resistant Soybean Background Boosts Inherent Resistance Against Hg 2.5.7 Soybean Cyst Nematode without Yield Penalties in Greenhouse**

Haarith, Deepak and A. F. Bent

Department of Plant Pathology, University of Wisconsin-Madison, Madison, WI 53726

## Abstract

Soybean cyst nematode populations with an HG Type of 2.5.7 are an increasingly widespread problem in U.S. soybean production. HG 2.5.7 populations partially overcome soybean resistance mediated by the widely deployed *rhg1-b* SCN resistance locus. *Rhg1* has two resistant haplotypes, *rhg1-a* and *rhg1-b*, that are commonly deployed in commercial soybean varieties. The haplotypes differ structurally and possibly in their mode of action. One of the three constituent *Rhg1* genes encodes an α-SNAP protein that differs between the haplotypes. In our previous growth chamber studies, an *rhg1-a* type SCN-resistant soybean line (IL3849N) that overexpressed a transgenically added extra copy of either the *rhg1-a* or *rhg1-b* type α-SNAP had significantly lower female indices (FI) compared to the already resistant parental line. We have now completed three greenhouse assays, with four replicates in each, to examine the yield characteristics and nematode suppression over full growing seasons. Overexpression of either type of α-SNAP did not have any yield penalties in terms of seed biomass either with or without HG 2.5.7 SCN at 1500 eggs/100cc initial inoculum level. Furthermore, the protein, oil, fiber, and moisture content also had no significant differences in either of the overexpression lines, compared to the parent line. Between the parent line IL3849N and IL3849N overexpressing the *rhg1-a* type α-SNAP there were no significant differences in nematode reproduction (eggs/100 cc of soil at the end of the season). However, overexpressing *rhg1-b* type α-SNAP significantly boosted the inherent resistance of the *rhg1-a* type IL3849N parental line against HG 2.5.7 SCN.

**TYMIRIUM™ Technology, a Novel Seed Treatment for Control of Nematodes in Cotton**

Hamill, Jon^1^, S. Moore^2^, M. Gaberthueel^3^ and J. Simmons^4^

^1^Syngenta Crop Protection, 410 Swing Road, Greensboro, NC 27409

^2^Syngenta Crop Protection, Monroe, LA

^3^Syngenta Crop Protection AG, Rosentalstrasse 67, 4002 Basel, Switzerland

^4^Syngenta Crop Protection, Vero Beach Research Center, Vero Beach, FL

## Abstract

Nematodes are among the most damaging pests to cotton production in both the USA and worldwide. Estimates for yield losses can be as high as 10.7% of the total production across cotton producing areas globally. Crop rotation, resistant cultivars, and chemical management have been the backbone of cotton production systems to manage nematodes. Due to the cost effectiveness and ease-of-use, several chemical seed-treatment nematicides have been adopted by growers to manage nematodes, including the seed treatments AERIS^®^, AVICTA^®^, and COPEO^®^. These seed treatment chemical nematicides have all been recognized to be effective in managing nematodes in cotton in conjunction with crop rotation and resistant/tolerant cultivars. Over the past 6 years, both field and greenhouse trials have been conducted to demonstrate the activity of TYMIRIUM™ technology, a new optimized carboximide developed by Syngenta, for management of nematodes in cotton. TYMIRIUM™ technology has demonstrated a high degree of efficacy on both Reniform, *Rotylenchulus reniformis* and Root-knot, *Meloidogyne incognita* nematodes with significant to favorable results for nematode control, 85.7% of field trials (n=43) and lint yield in 81.3% of field trials (n=43). Direct comparison of TYMIRIUM™ technology and COPEO^®^ seed treatment have demonstrated a mean yield increase of 116 Kg/ha, compared to 40 Kg/ha, in favor of TYMIRIUM™ technology, with both treatments compared to fungicide and insecticide only plots (n=50).

**Evaluating Potential Soil Health Benefits of Non-Fumigant Nematicides in Louisiana Sweetpotato Production**

Hamm, Caleb^1^, J. C. Gregorie^2^ and T. T. Watson^1^

^1^Department of Plant Pathology and Crop Physiology, LSU AgCenter, Baton Rouge, LA 70803

^2^Sweet Potato Research Station, LSU AgCenter, Chase, LA 71324

## Abstract

Plant parasitic nematodes are major pests of *sweetpotato* worldwide. In the United States, the reniform nematode, *Rotylenchulus reniformis*, and the southern root-knot nematode, *Meloidogyne* incognita, are two of the most damaging nematodes on *sweetpotato*. Plant parasitic nematodes are most often managed by using nematicides to lower nematode populations. The goal of this research is to evaluate the efficacy of currently registered nematicides for sweetpotato production and the effects that those nematicides have on soil health. To evaluate efficacy of *nematicides*, a field trial was conducted where seven nematicides were applied before planting, and reniform and root-knot nematode populations were quantified and compared to a non-treated control at four soil sampling dates throughout the 2021 growing season. To evaluate nematicide effects on soil health, free-living nematode trophic groups were quantified and two different soil suppressiveness assays (plant-based and soil-based assays) were conducted to evaluate the soil’s ability to suppress introduced root-knot nematodes. Results from the 2021 field trial showed no significant differences in reniform nematode soil population densities or yield of US#1 sweetpotatoes due to treatment; however, Velum and Majestene showed a strong numerical trend toward increased total sweetpotato yield (US#1, Jumbo, and Canner grade). Nematicide application did not impact soil population densities of free-living nematode trophic groups or soil suppressiveness in the plant-based and soil-based assays, suggesting that the nematicides did not negatively impact these aspects of soil health. In 2022, this field trial was repeated, along with two complimentary greenhouse nematicide efficacy trials using naturally infested field soil from two separate locations. Preliminary results from the 2022 experiments will be presented.

**First Report of *Punctodera Stonei* Brzeski, 1998 (Nematoda: Heteroderidae) From Virginia, U.S.A**.

Handoo, Zafar A.^1^, M. Kantor^1^, S. Subbotin^2^ and G. Huse^3^

^1^Mycology and Nematology Genetic Diversity and Biology Laboratory, USDA, ARS, Northeast Area, Beltsville, MD 20705

^2^Plant Pest Diagnostic Center, California Department of Food and Agriculture, 3294 Meadowview Road, Sacramento, CA 95832

^3^Arlington National Cemetery, 1 Memorial Avenue Arlington, VA 22211

## Abstract

In August 2021, eight soil samples were collected during a vegetation survey conducted at the Arlington National Cemetery and the samples were processed at the Mycology and Nematology Genetic Diversity and Biology Laboratory (MNGDBL) at Beltsville, Maryland. Among other plant-parasitic nematodes the soil sample contained few cysts and juveniles of cyst nematode. The analyzed cysts were light to dark brown in color, oval-shaped without bullae and with a prominent vulval cone. In older cysts bullae could be observed. The second-stage juveniles had slightly concave stylet knobs, tail tapering gradually to a narrowly rounded terminus, and hyaline tail terminus conspicuous at least twice the length of stylet. The molecular analysis included sequence and phylogenetic analysis of internal transcribed spacer (ITS rRNA), D2-D3 expansion segments of 28S rRNA and mitochondrial cytochrome oxidase I (*COI* mtDNA) genes. The nematode species was identified by both morphological and molecular means as *Punctodera stonei*. To our knowledge this represents the first report of *Punctodera stonei* from the United States.

**Above- And Belowground Herbivores Influence Plant Chemistry Changes in *Solanum Tuberosum***

Hauri, Kayleigh and Z. Szendrei

Michigan State University, Dept. of Entomology, East Lansing, MI 48824

## Abstract

Plants are regularly challenged by both aboveground and belowground herbivores, but the outcomes of these interactions vary significantly based on species identity and host plant traits. We used laboratory experiments with two genetically modified potatoes, one that contains the glycoalkaloid leptine - a secondary metabolite that provides resistance to *Leptinotarsa decemlineata* (Colorado potato beetle) - and one that does not. Leptine is particularly of interest to plant breeders because it is produced in aboveground tissues only, but it is unclear how the effectiveness of this trait will interact with belowground herbivory, which can alter plant defenses and nutrition. We exposed each line to no herbivory, aboveground herbivory by *L. decemlineata*, belowground herbivory by *Meloidogyne hapla* (Northern root knot nematode), or both. We predicted that *L. decemlineata* would perform better on plants without leptine, but that *M. hapla* presence would reduce this difference. Additionally, we predicted that above- and belowground herbivory would both alter volatile production and internal chemistry in distinct ways. We assessed herbivore performance as well as plant internal chemistry and headspace volatiles. Understanding how plant chemistry mediates aboveground-belowground interactions between herbivores will inform effective multiple-pest management strategies.

**Survey of Plant Parasitic Nematodes in Delaware and Maryland Corn Acreage**

Henrickson, Madeline G. and A. M. Koehler

University of Delaware Department of Plant and Soil Sciences, 16483 County Seat Highway Georgetown, DE 19947

## Abstract

Plant parasitic nematodes reside and thrive within the sandy soils found across Delaware (DE) and eastern shore Maryland (MD). Nematode surveys were conducted in soybean from 2019-2020. In addition to anticipated soybean cyst nematodes, lance, root knot, stubby root, and lesion nematodes were present across the region. Since many of these nematodes can infest corn and no prior corn nematode surveys had been conducted in the region, a survey was initiated to identify the prevalence and distribution of plant parasitic nematodes in corn. In 2021, nematode samples were obtained from 66 fields across DE and MD. Ten nematode genera were recovered: lance, lesion, root knot, stunt, spiral, stubby root, dagger, soybean cyst, pin, and ring. Lance nematodes were most notable, recovered in 73% of fields, with 12% of fields containing population levels above high damage threshold and 23% at medium threshold. Stunt nematodes were recovered in 74% of fields, with 5% above high damage threshold. Lesion nematodes were present in 56% of fields, but with populations at low threshold. Root knot and stubby root nematodes were identified less frequently with 3% and 5% of positive samples above high threshold, respectively. This work provides a preliminary data set in corn to connect which nematode genera are most prevalent in damaging numbers throughout the region. Nematode surveys will be continued in 2022 to further understand the effect of plant parasitic nematodes on corn production in DE and MD.

**What Worms and their Microbes can Teach us about Symbiosis**

Heppert, Jennifer K.^1^, O. Alani^1^, T. Myers^1^, M. Cao^2^, G. Chen^3^ and H. Goodrich-Blair^1^

^1^Department of Microbiology, University of Tennessee-Knoxville, Knoxville, TN 37996

^2^Division of Biology and Biological Engineering, California Institute of Technology, Pasadena, CA 91125

^3^NOMIS Center for Immunobiology and Microbial Pathogenesis, The Salk Institute for Biological Studies, La Jolla, CA 92037

## Abstract

Animals live in symbiotic cooperation and conflict with microbes inhabiting their environment and colonizing their tissues. Nematodes are attractive models for discovering both unique and universal mechanisms that underlie these animal-microbe symbioses because of their incredible taxonomic, habitat, trophic, and lifecycle diversity. Entomopathogenic nematodes, or nematodes that prey on insects, including *Steinernema* nematodes, have been particularly fruitful models for symbiosis research. The symbiosis is a naturally occurring mutualism between individual *Steinernema* nematode species and specific *Xenorhabdus* bacterial partner species. Together, the nematodes and bacteria infect insect prey. The nematodes carry the bacteria within an intestinal pocket and, upon infection, release them into a new insect host. The bacteria aid in parasitizing and catabolizing the insect and act as the nematodes’ primary food source. At the end of each infection cycle, the nematodes and bacteria reassociate to search for new insect prey. This experimentally tractable, naturally occurring system provides ample opportunity to mechanistically study important processes related to symbiotic relationships such as colonization, transmission, competition, and host-switching. The large array of known nematode-bacterial pairs, and the ability to raise nematodes and bacteria in the lab, either both together or each individually, further enables comparative biology and coevolution studies. Recently, we have been collaborating to develop a functional genetic model system based on a newly re-isolated species of nematode, *Steinernema hermaphroditum*, and its *Xenorhabdus griffiniae* bacterial symbiont. We will report on our efforts to characterize and manipulate the *X. griffiniae* bacterial symbiont genome through sequencing, generation of specific mutants and genetically modified strains, and the creation of screenable mutant libraries. We will describe the use of these tools and others under development, along with genetics in *S. hermaphroditum*, to uncover molecular mechanisms central to symbiotic lifestyles.

**Evaluating Trap Crops and Crop Rotations for Potato Cyst Nematode Eradication in Idaho**

Hickman, Paige and L. M. Dandurand

University of Idaho, Dept. of Entomology, Plant Pathology, and Nematology, Moscow, ID 83844

## Abstract

*Globodera pallida*, the pale cyst nematode is a species of potato cyst nematode (PCN) present and quarantined in Idaho. PCN has the potential to cause devastating yield loss and can remain viable in the soil for decades. The main focus for PCN in Idaho is containment through phytosanitary measures and eradication through soil fumigation. Research efforts for new strategies to eliminate PCN are of high importance. Trap crops and crop rotations have potential as feasible strategies for PCN control. *Solanum sisymbriifolium*, has been shown to significantly reduce PCN. *Chenopodium quinoa* was also found to induce PCN hatch, although the research on value as a trap crop is limited. This study seeks to compare efficacy of litchi tomato and quinoa as trap crops. Three-year crop rotations with litchi tomato and a resistant potato variety are also being evaluated in the field to determine impact on PCN population decline. The effect of these treatments on PCN are evaluated through encysted egg counts, egg viability assays, hatching assays, and enumeration of reproduced cysts following a susceptible potato. The first in-field comparison of litchi tomato and quinoa showed that after susceptible potato, quinoa plots had 40% less PCN reproduction than non-host plots, while litchi tomato plots had 97% less reproduction. Viability assays show similar trends in that litchi tomato reduces viable eggs significantly more than quinoa, however quinoa reduces eggs significantly more than the nonhost control. This suggests that while it is not as effective as litchi tomato, quinoa may substantially decrease PCN. The three-year crop rotations are still underway, but seasonal cyst evaluations show both litchi tomato and resistant potato reduce the initial PCN population dramatically after year one. Annual soil samples indicate no reproduction on litchi tomato and low reproduction on the resistant potato, meaning overall PCN population is reduced. The third year in rotation will be a susceptible potato, after which, soil will be extracted to determine reproduction. Ultimately, Idaho potato growers with infested acreage may be able to employ these strategies in the future to help eradicate PCN from their fields.

**Determining the Effect of Suppressive Soils in Ornamental (*Hemerocallis* Spp.) Fields for Northern Root-Knot Nematode (*Meloidogyne Hapla*) Management**

Howland, Amanda D., E. Cole and M. Quintanilla

Michigan State University, Department of Entomology, East Lansing, MI

## Abstract

In the United States, the floriculture industry was valued at $4.8 billion in 2020. Michigan is the third largest producer of the floriculture industry, and a major component of that industry is the production of bare-rooted daylilies, *Hemerocallis* spp. However, production of clean nursery plant material is challenging for bare-root ornamental crops grown under field conditions due to plant-parasitic nematodes. In northern North America, the northern root-knot nematode, *Meloidogyne hapla*, is one of the main pathogens in the floriculture industry causing over 20% yield loss. Previous field trials at a commercial nursery in Zeeland, MI showed possible *M. hapla* suppression occurring in long-term ornamental fields. To determine the impact of these possible suppressive soils on *M. hapla* management, a greenhouse trial was established in 2021. Field soil was obtained from an ornamental field at two time points: two weeks before the field was fumigated with Telone^®^ and two weeks after application. Nursery-grade first-year bare-rooted *Hemerocallis* spp. cv. ‘Going Bananas’ plants were potted directly into the field soil in 3.7 L pots. Each pot was inoculated with 9,000 *M. hapla* eggs/pot and two weeks after inoculation, treatments were applied to each respective pot. Treatments applied were based on the results of previous field trials that showed the best *M. hapla* management, plus a fungicide to determine if soil suppression is caused by a nematophagous fungus. Treatments applied were 1) Indemnify^®^, 2) Majestene 304^®^, 3) a high-carbon compost, 4) Bravo Weather Stik^®^ fungicide, and 5) a control. Pots were arranged in a randomized block design with eight replications for a total of 80 pots. Plants were kept at a 16h:8h light:dark photoperiod at 26°C. Bi-weekly plant evaluations were recorded to measure plant growth, such as plant height (cm), crown diameter (cm) N-S and E-W, number of eyes, number of buds, and number of scapes; the plant height and two crown diameters were taken to generate a growth index. This trial will be concluded in October of 2022. Final plant measurements will be taken along with a *M. hapla* gall rating and determination of final *M. hapla* and beneficial nematodes populations. Preliminary results show that the fumigated compost pots have the highest yield (in terms of number of eyes) and the fumigated fungicide pots have the lowest yield, while the fumigated Majestene 304 pots have the smallest growth index and the non-fumigated Indemnify have the largest growth index. The final results of this study will provide great insight to help determine if any natural suppression is occurring in long-term ornamental fields which can be used to develop effective management strategies for *M. hapla*.

**Gene Annotation and Comparative Genome Analyses of Different Races of *Meloidogyne Chitwoodi***

Hu, Shengwei^1^, S. Bali^2^, C. Gleason^2^, E.T.J. Danchin^3^, K. Vining^4^ and V. Sathuvalli^1^

^1^Oregon State University, Dept. of Crop and Soil Science, Hermiston, OR 97838

^2^Washington State University, Dept. of Plant Pathology, Pullman, WA 99164

^3^Institut Sophia Agrobiotech, Université Côte d’Azur, INRAE, CNRS, Sophia Antipolis, France

^4^Oregon State University, Dept. of Horticulture, Corvallis, OR 97331

## Abstract

Columbia root knot nematode (CRKN, *Meloidogyne chitwoodi*) parasitizes potato plants and causes small brown dots in the tuber flesh that dramatically reduces the market value of the crop. In the Pacific Northwest, two races of *M. chitwoodi*, Race 1 and Race 2 exist and are differentiated based on their host range. A pathotype of Race 1, Race 1^Roza^ was also isolated from the fields grown under the potato clones carrying the resistance to Race1 and Race2. In order to understand the phylogeny of *M. chitwoodi* and develop molecular markers to identify the different races, we sequenced the genomes of *M. chitwoodi* Race 1, Race 2 and Race 1^Roza^ using Illumina and PacBio sequencing. The final genome assembly were 47.47 Mb for Race 1 (30 contigs), 46.98 Mb for Race 2 (39 contigs), and 47.78 Mb for Race 1^Roza^ (38 contigs). All three nematode genomes contain an average 25% GC content. We annotated these genomes using RNAseq data from CRKN second-stage juveniles (J2) and validated proteome files. The genome annotation averaged 11,000 genes across the three genomes. The mitochondrial genomes of Race 1, Race 2 and Race 1^Roza^ were also assembled from PacBio sequencing data with over 90% sequence similarity from the existing reference. Comparisons of ortholog genes and mitochondrial genomes provided new evidence to elucidate the complex evolutionary history of this species and analyze host plant resistance.

**Nitrogen Uptake in C3 Plants Under CO_2_ and Nematode Stress**

Hughes, Kody, S. Mishra and P. M. DiGennaro

Department of Entomology and Nematology, University of Florida, Gainesville, FL 32611

## Abstract

Increasing levels of atmospheric CO_2_ stimulates growth resulting in more biomass in some plants; however, this can also lower nutrition levels as the ratio of carbon to nitrogen content decreases. Lower nutrient levels can mean more plant mass must be consumed to meet nutrient requirements, both for the humans that depend on these crops and for plant pests, increasing their impact on crop quality and yield. Understanding how abiotic stressors (high CO_2_ levels) and biotic stressors (plant pests) interact is vital to preparing management strategies to mitigate potential decreases in yield and quality under projected increasing global CO2 levels. Here I employed root-knot nematode (RKN) due to its agricultural significance, ubiquity, and wide host range in a tomato pathosystem. Under four different CO_2_ levels, we labeled control and RKN-infected tomato plants with heavy nitrogen and quantified plant and nematode nitrogen assimilation rates with high-throughput proteomics. We also examined root and shoot weight ratios and gall ratings. Comparisons of heavy labeled nitrogen isotope ratios and characterization of assimilation rates, nematode proliferation, and weight ratios yield novel insights into the potential impacts to crop yield and quality as our climate changes over the next century.

**Efficacy of Biologically-Active Nematicides Against Sting Nematodes, *Belonolaimus Longicaudatus* Infecting Bermudagrass, *Cynodon Dactylon* under Greenhouse and Field Conditions**

Jagdale, Ganpati^1^, B. Vermeer^2^, K. Martin^1^, A. D. Martinez-Espinoza^2^, P. M. Severns^1^ and D. Shapiro-Ilan^3^

^1^Department of Plant Pathology, University of Georgia, Athens, GA 30602

^2^Department of Plant Pathology, University of Georgia, Griffin GA 30223

^3^USDA-ARS, Byron, GA 31008

## Abstract

There is increasing interest among the turfgrass managers in using novel and environmently friendly biologically-active nematicides to manage economically important plant-parasitic nematodes (PPNs) especially sting (*Belonolaimus longicaudatus*) and ring (*Mesocriconema* spp.) nematodes in turfgrasses. We compared the efficacies of biologically- active nematicides including Nortica^®^ 10 WP, (a.i.- living bacteria: *Bacillus firmus*), Zelto^®^ (a.i.- heat-killed *Burkholderia* spp. strain A396) and MeloCon^®^ WG (a.i.- fungus: *Paecilomyces lilacinus*), compost, and the metabolites from two entomopathogenic bacteria (*Xenorhabdus szentirmaii* and *X. bovienii*) against conventional non-fumigant nematicides, Nimitz® (a.i. Fluensulfone) and Indemnify^TM^ (a.i. Fluopyram) at two different rates on sting and ring nematodes under greenhouse and field conditions. Under greenhouse conditions, population densities of sting nematodes were suppressed to the lowest levels (≤ 2 nematodes/100 cm^3^ soil) in pots treated with both low (2.25 kg/ha) and high (9.88 kg/ha) rates of MeloCon and a high rate of *X. bovienii* metabolite (449.72 L/ha), but they did not statistically differ when compared to the control and all other treatments except a high rate of compost treatment 15 days after treatment (DAT). Furthermore, only a high rate of MeloCon WG consistently suppressed sting nematode populations to ≤ 2 nematodes/100 cm^3^ soil from 15 to 120 DAT. Under field conditions, the plots treated with the high and low rates of MeloCon WG, low rate of Indemnify (0.62 L/ha), and high rates of Nimitz (135.61 kg/ha), Zelto (9.36 L/ha), metabolites of *X. szentirmaii* and *X. bovienii* bacteria (449.72 L/ha) suppressed sting nematode populations compared to the untreated control 30 DAT, but not at 120 DAT. In most treatments, reproduction of sting nematodes appeared to be suppressed only for a short-term (30 DAT) under field conditions. In contrast, sting nematode reproduction was not significantly suppressed in all the treatments under greenhouse conditions either 30 or 120 DAT. None of the products negatively affected the population densities of ring nematodes compared with the untreated control under both greenhouse and field conditions. In conclusion, only one bio-nematicide (MeloCon WG) appeared to consistently suppress sting nematodes under both greenhouse and field conditions, but the effect was short-lived under field conditions. Our results suggest that MeloCon WG may be effective for controlling sting nematodes, but repeated applications may be necessary for field efficacy. More studies are needed to confirm these results and improve their long-term efficacies against PPNs.

**Response Comparison of *Pratylenchus Neglectus* Pathotypes on Winter Wheat, Pea, Lentil, and Barley**

Johnson, Hannah, E. Consoli and A. T. Dyer

Montana State University, Dept. of Plant Sciences and Plant Pathology, Bozeman, MT 59717

## Abstract

In a 2016 survey, three pathotypes of *Pratylenchus neglectus* were found in Montana’s winter wheat fields, i.e., populations able to multiply on i) wheat; ii) wheat and barley; and iii) wheat, barley and lentils. Here we present a histological study aimed at comparing the root invasion between two of the three *P. neglectus* pathotypes including a wheat pathotype (P1) and a wheat-barley-lentil pathotype (P3). These pathotypes were collected from infested fields that showed higher than average nematode densities - more than 1000 *P. neglectus/*kg soil - and were maintained in pot cultures under greenhouse conditions. Nematodes were extracted from plant roots and the resulting extract with mixed developmental stages were used as inoculum. The experiments were carried in 66 cc conetainers using a sterilized loam field soil combined with sand in a 1:1 mixture. Treatments consisted of the two *P. neglectus* pathotypes (P1 and P3), four harvest dates, and five crop treatments: two winter wheat double haploid lines (DH39 susceptible and DH60 resistant), pea cv. Delta, barley cv. Harrington, and lentil cv. Richlea. Each treatment has four replicates arranged in a randomized block design. To establish the trials, seedlings were surface sterilized in 10% bleach, pre-germinated on moist paper, and transplanted into individual cone-tainers four days post germination. One week after transplanting, approximately 150 *P. neglectus* (mixed stages) were inoculated per cone-tainer. Non-inoculated plants were used as negative controls. The four sampling time points were at four days, and two, four and six weeks after inoculation. *P. neglectus* invasion was assessed by root staining with acid fuchsin. At the fourth time point, nematodes were extracted from roots and their numbers were measured. Overall, populations of P3 were statistically greater for all the tested crops compared to populations of P1 (*p*-value = 0.034). Interestingly, although pea was found as a resistant crop to *P. neglectus* in our previous studies, we identified *P. neglectus* invading pea roots in the P3 treatment. However, no statistical differences were found when comparing nematode counts across crops for each population (*p*-value = 0.54), likely due to the high variability observed. Although crop damage was not assessed, these results suggest that *P. neglectus* P1 and P3 behave differently with regards to the root invasion, and this might play a significant role in the capabilities of the pathotypes found in Montana fields. The emergence of pathotypes and the phenotypic differences demonstrated by these trials highlight the increasing need for better management alternatives, and better understandings of genetic changes behind the emergence of virulent populations of *P. neglectus*.

**Molecular and Morphological Characterization of Fresh and Fixed Specimens of *Sauertylenchus Maximus* (Allen, 1955) Siddiqi, 2000 From Virginia, United States**

Kantor, Mihail R.^1^, P. Vieira^1^, A. Skantar^1^, G. Huse^2^ and Z. Handoo^1^

^1^Mycology and Nematology Genetic Diversity and Biology Laboratory, USDA, ARS, Northeast Area, Beltsville, MD 20705

^2^Arlington National Cemetery, 1 Memorial Avenue, Arlington, VA 22211

## Abstract

During a survey of natural grass conducted at the Arlington National Cemetery, Virginia in 2000 and 2021 for type specimens of *Xiphinema americanum* and *Hoplolaimus galeatus*, adult and juvenile specimens of *Sauertylenchus maximus* (Allen, 1955) Siddiqi, 2000 were discovered. In the present study, we characterize fresh and fixed specimens of *Sauertylenchus maximus* collected from the Arlington National Cemetery, VA by molecular and morphological means. Fresh females and juveniles of *S. maximus* were recovered from the root of *Festuca arundinacea* L. (tall fescue) and soil samples using the sugar centrifugal flotation. The fixed specimens used in this study were deposited in the United States Department of Agriculture Nematode Collection (USDANC), without any further identification. Morphology and morphometrics of specimens collected in VA were consistent with the original and other descriptions of this species. Phylogenetic analysis of 18S rDNA, internal transcribed spacer (ITS) and 28S large subunit ribosomal DNA sequences validate the identification of both fresh and fixed specimens as *S. maximus*. This represents the first report of *S. maximus* from Virginia, U.S. and the first report of a successful DNA extraction from fixed nematode specimens deposited in the USDANC.

**Bioinformatic Analysis of *Cardinium* Symbionts in Soybean Cyst Nematodes**

Kaur, Amandeep, and A.M.V. Brown

Department of Biological Sciences, Texas Tech University, Lubbock, TX

## Abstract

Plant-parasitic nematodes (PPNs) are agriculturally important pests resulting in significant crop yield loss costing $100B annually. Despite the considerable losses caused by nematodes in agriculture, they have not received much attention in advanced genomic or transcriptomic research programs – few species have been sequenced. PPNs harbor endosymbionts such as *Wolbachia*, *Cardinium*, *Xiphinematobacter*, and others, which are thought to be important players in some of the most significant nematode pests. This study examined metagenomes from soybean cyst nematode (SCN), *Heterodera glycines*, which causes yield loss of up to 50% and is difficult to control, making its endosymbiont *Cardinium* (Bacteroidetes) a focus for potential control of SCN populations. The endosymbiont *Cardinium* from the SCN may be important as a reproductive manipulator of SCN, but to date, only one published genome is available. In this study, DNA was extracted from 15 different populations of SCN and sequenced using Illumina, yielding 27.3 to 67 million raw reads per sample. Based on initial results that revealed *Cardinium* endosymbionts in these samples, the data were bioinformatically analyzed in further detail to try to retrieve high quality draft genomes of *Cardinium* strains that will enable comparative population genomics of this endosymbiont to better understand its potential role. Raw reads were filtered then assembled with metaSPAdes, then to extract improved draft genomes, binning and re-assembling was performed using metaWRAP. One third of SCN isolates produced nearly complete *Cardinium* genomes from a custom in-house bioinformatic pipeline while a further 2/3 of samples had fragments of *Cardinium* suggesting past horizontal gene transfers. MetaWRAP generated one high-quality *Cardinium* genome (cHgF63) with size 1.3 Mb, sharing 763 and 401 genes with strain cHgTN10 from SCN and cPpe from a root lesion nematode, respectively. Genomes cHgF63 and cHgTN10 revealed 33 and 83 unique predicted genes, respectively, suggesting potentially important functional divergence across populations. Ongoing population genomics on these strains will potentially reveal critical genes with host-manipulation functions that may inform strategies to aid symbiont-based integrated pest management and biocontrol.

**Screening of Pepper Lines for Peach Root-Knot Nematode Resistance**

Khanal, Churamani^1^, W. Rutter^2^, M. Alam^1^ and I. Alarcon^1^

^1^Clemson University, Dept. of Plant and Environmental Sciences, Clemson, SC 29634

^2^USDA-ARS, U.S. Vegetable Laboratory, Charleston, SC 29449

## Abstract

Six pepper (*Capsicum annum*) lines/cultivars (PM217, PA136, PM687, ‘Charleston Belle’, HDA149, and HDA330) carrying root-knot nematode (*Meloidogyne* spp.) resistance gene(s) were screened in a growth room environment against the peach root-knot nematode (*M. floridensis*). The USDA line PA136 susceptible to root-knot nematodes was used as a control. Six-week-old pepper lines with four replications of each were grown in six-inch plastic pots containing 1.5 kg steam-sterilized soil and were inoculated with 10,000 eggs of *M. floridensis*. The number of eggs per g of fresh roots two months after inoculation ranged from 167 to 54,222. Pepper lines HDA330, PM687, and PM217 including the control had lower *M. floridensis* reproduction compared to HDA149 and Charleston Belle. PM217 supported the least number of eggs (444/g root), followed by PM687 (454/g root), and HDA330 (10,259). Reproduction of *M. floridensis* was the greatest in HDA149 (46,275/g root) and intermediate in ‘Charleston Belle’ (25,434/g root) while the control had 456 eggs/g root. The lines PM217 having root-knot nematode resistance genes *Me1* and *Me2* as well as the control had no visible galls suggesting these lines have a high level of resistance against *M. floridensis*. On a scale of 0-10 (0 = no galls, 10 = completely galled roots), the average gall rating for PM687 carrying *Me3* and *Me4* genes as well as HDA330 carrying *Me1* gene was 3, an indication that these lines are moderately resistant to *M. floridensis*. ‘Charleston Belle’ carrying the *N* gene and HDA149 carrying *Me3* gene received an average gall rating of 5.2, suggesting these lines are susceptible to *M. floridensis*. Results from this study will be useful in *M. floridensis* resistant pepper breeding programs.

**Effects of the Root-Lesion Nematode, *Pratylenchus Penetrans*, on Early Growth, Photosynthesis and Water Relations of ‘Ambrosia’ Apple Trees on Three Different Rootstocks**

King, Lindsay^1,2^, P. Munro^1^, M. Jones^2^, H. Xu^1^ and T. Forge^1^

^1^Agriculture and Agri-Food Canada, Summerland Research and Development Centre, 4200 Hwy 97, Summerland, BC V0H 1Z0, Canada

^2^Biology Department, University of British Columbia – Okanagan, Kelowna, BC V1V 1V7, Canada

## Abstract

The root-lesion nematode, *Pratylenchus penetrans*, is recognized as a widespread pest of most temperate fruit trees. It is known to contribute to the broader replant disease complex that can significantly impair establishment of seedlings replanted into old orchard sites where populations of *P. penetrans* and other root pathogens had previously built up. The impacts of *P. penetrans* on mature trees are difficult to quantify but it is suspected of contributing to decline syndromes of mature trees such as the Rapid Apple Decline syndrome. For decades most apple acreage in the Pacific Northwest and British Columbia has been planted with M.9 rootstock. Recently growers have become increasingly interested in the Cornell-Geneva series rootstocks such as G.41 and G.935, owing to their apparently greater resistance or tolerance to apple replant disease. However, the responses of these rootstocks to *P. penetrans*, specifically, are poorly known. We used a field micro-plot experiment to compare the resistance and tolerance of G.41, G.935 and M.9 rootstocks (‘Ambrosia’ scion) to *P. penetrans*. The experiment was established in spring of 2020 at the Summerland Research and Development Centre. The experimental design was a 2 x 3 factorial combination of: *P. penetrans* inoculation (+/-) and rootstock (G.41, G.935, M.9), with 20 replicate micro-plots of each of the six treatment combinations arranged in a randomized complete block design. A subset of micro-plots were fitted with minirhizotron access tubes to facilitate quantification of root growth. The *P. penetrans* inoculum was 5400 *P. penetrans*/microplot or 54/L soil. Analyses of vegetative growth parameters (trunk cross sectional area, shoot length growth, leaf surface area, root length, root surface area) and physiological parameters (stomatal conductance, transpiration, photosynthesis, stem water potential) over two years revealed few rootstock × *P. penetrans* interactions, suggesting that these rootstocks are equally susceptible to *P. penetrans*. There were main-factor effects of *P. penetrans* on most parameters. After the first year of growth (2020), *P. penetrans* reduced trunk cross sectional area, shoot length growth and leaf surface area. In the second year of growth (2021), shoot length growth, root length and surface area, and tree physiological parameters (stomatal conductance, transpiration, photosynthesis, stem water potential) were all reduced by *P. penetrans*. Our results indicate that *P. penetrans* has modest but chronic effects on growth and physiological processes. The impacts on physiological processes relating to water relations and carbon balance suggest mechanisms by which *P. penetrans* may predispose trees to other stresses and contribute to replant and decline disease complexes.

**Pool-Seq Analysis for the Discovery of Candidate Genes Involved in Virulence of *Heterodera Glycines* on Resistant Soybean**

Kwon, Khee-Man^1^, W. W. Lorenz^2^ and M. G. Mitchum^1^

^1^University of Georgia, Dept. of Plant Pathology and Institute of Plant Breeding, Genetics and Genomics, Athens, GA 30602

^2^University of Georgia, Georgia Genomics and Bioinformatics Core, Athens, GA 30602

## Abstract

The soybean cyst nematode (SCN), *Heterodera glycines*, is a major pest of soybean most effectively managed through resistant cultivars. However, the repeated planting of the same resistance sources, predominantly plant introduction (PI) 88788 (>95% of varieties) and PI 548402 (Peking; <5% of varieties), has exerted a selection pressure on SCN populations which led to a widespread problem of virulence. While the PI 88788-type resistance only requires *Rhg1*, the Peking-type requires an epistatic interaction between *Rhg1* and *Rhg4* mediated by an α-SNAP (α-soluble N-ethylmaleimide sensitive factor attachment protein) and serine hydroxymethyltransferase, respectively, to confer resistance to SCN. The nematode genes responsible for virulence on these two major resistance types remain unknown; understanding the genetic determinants of adaptation to resistances can improve management strategies to reduce crop yield losses caused by this pathogen. To identify the genomic regions and genes potentially responsible for virulence on the Peking-type (*Rhg4*-mediated) resistance, we conducted a cost-effective method for whole genome-resequencing pools of individuals (Pool-Seq) on two pairs of independently derived avirulent and virulent populations adapted to the *Rhg4*-mediated resistance. Well-established software packages specifically designed for population genetic analysis of pooled samples were chosen to identify SNPs and determine their frequencies between populations. Further analysis is underway to filter the SNPs to identify regions of the genome under selection. The identification of virulence genes may provide insights into the mechanisms by which soybean cyst nematodes mediate host-pathogen interactions to overcome soybean resistance. Moreover, these virulence genes may be developed into molecular markers to rapidly determine the virulence profile of a SCN population, a much-preferred alternative to the laborious and time-consuming greenhouse bioassay.

**Increased Diversity in Ventral Nerve Cord Complexity Among Basal Clade Nematodes**

Lanzatella, Marissa, J. Han and N. Schroeder

University of Illinois at Urbana-Champaign, Urbana, IL 61873

## Abstract

The nervous system of nematodes is highly conserved; however, most neuroanatomical studies have focused on nematodes in Clades 8-12 (Holterman et al., 2006; van Megen et al., 2009). Scattered evidence in the literature suggests that nematodes in Clades 1 and 2 possess more neurons than those in Clades 8-12. We hypothesized that nematodes underwent a simplification of the nervous system during the evolution of Clades 8-12. To test this hypothesis, we used fluorescent microscopy to examine the ventral nerve cord (VNC) in Clades 1-6 and compared these with previously published data on nematodes in Clades 8-12, including the model organism *Caenorhabditis elegans* (Clade 9). Nematodes were isolated from diverse habitats and adult females were identified to the family level. Nematodes were fixed and stained with 4’,6-diamidino-2-phenylindole (DAPI) and neuronal-like nuclei along the ventral ridge were counted. In total, we isolated nematodes from seventeen families in Clades 1-6 for our analysis. No specimens from Clade 7 were found. While several families had substantially more VNC neurons than the 57 found in *Caenorhabditis elegans*, many families showed a similar number or fewer VNC neurons. The largest number of VNC neurons was found in Clade 2 (Longidoridae: 265-304 neurons), while the smallest number were found in Clade 1 (Prismatolaimidae: 28-29 neurons). When analyzed as a function of nematode length, we found several families with significantly greater neuronal density than *C. elegans*; however, this analysis did not find a consistent pattern of VNC evolution across the phylum. None of the isolated Clade 1 nematodes (Diptherophoridae, Prismatolaimidae, Tobrilidae) had a greater neuronal density than *C. elegans*. Among Clade 2 nematodes examined, the Longidoridae and Mononchidae had significantly greater VNC neuronal densities than *C. elegans*. Interestingly, among Clades 3-6 (basal Chromadoria), the Camacolaimidae, which represents our only family isolated from marine sediment, had a greater VNC density than *C. elegans*. Together, our data did not support a onetime evolutionary simplification of the nervous system between basal and derived clades. Rather, we suggest that nematodes in the basal clades have a greater diversity of neuronal structure. We plan to increase the coverage of the phylum in future studies, particularly of marine nematodes, and to expand our examination of the nervous system beyond the VNC.

**Parasitic Variability of *Meloidogyne Hapla* Relative to Soil Groups and Soil Health Conditions**

Lartey, Isaac^1^, A. Kravchenko^2^, G. Bonito^2^ and H. Melakeberhan^1^

^1^Department of Horticulture

^2^Department of Plant Soil and Microbial Sciences, Michigan State University, East Lansing, MI 48824

## Abstract

Managing *Meloidogyne hapla* in temperate vegetable production systems remains challenging due to the ban of broad-spectrum nematicides, lack of resistant crops and its broad host range and the presence of parasitic variability (PV) where populations look similar genetically and morphologically but differ in their reproductive potential and pathogenicity. While it has been known that M. hapla is broadly distributed and that its PV has some relationship with soil types, what soil health conditions may be related to its PV are unknown. The working hypothesis was that PV in M. hapla populations is related to SFW conditions. In an experiment repeated three times, the M. hapla populations were inoculated at 2000 (low) and 4000 (high) eggs/300cm3 of soil. Results showed that both the high and the low inoculum treatments had three categories of reproductive potential: the highest (Population 13), medium (Population 8), both from degraded mineral soil, and lowest from disturbed mineral (Population 2) and disturbed (Populations 4, 6 and 10) and degraded (Populations 5, 14 and 15) muck soils. The hypothesis was true for soil health and PV in mineral soils. This seems to suggest that other factors could be related to PV. Hence, the study lays the foundation for exploring the potential biophysicochemical conditions associated with M. hapla occurrence and PV.

**Plant Parasitic Nematodes Intercepted in Uk Trade, 2020-2022**

Lawson, Rebecca and T. Prior

Fera Science Ltd., Nematology Dept., National Agri Innovation Campus, Sand Hutton, York, YO41 1LZ, UK

## Abstract

Annually, Fera processes over 50,000 statutory samples for plant pests and diseases, around 5000 of which are for plant-parasitic nematodes. The review summarises significant nematode species found as part of import inspections in the United Kingdom (UK). Soil and plant samples relating to import inspections were collected by the Plant Health and Seeds Inspectorate. The very wide range of nematodes intercepted in UK trade poses a challenge to scientific services in plant health, from expert identification to PRA and effective solutions for their control. In addition, many intercepted species are not listed in international legislation and are little studied. Nematodes are also used as bio-indicators for non-compliance of phytosanitary conditions. It is evident that international trade provides a significant means of geographic spread for plant-parasitic nematodes. Prevailing trends for certain types of plant, as well as the choice of the most economic sites for their production, influences the risks that such trade poses. Current EU discussions pertaining to stricter controls at source raises the possibility of improved, sustainable control and consequent reduction in losses incurred.

**Comparative Bioactivity of Abamectin Formulations Against the Pine Wood Nematode, *Bursaphelenchus Xylophilus***

Lee, Jong Won^1^, M. Abraham Okki^1^, J. H. Choi^1^, H. Lee^1^ and D. W. Lee^1,2^

^1^Department of Entomology, Kyungpook National University, Sangju, Republic of Korea

## Abstract

In Korea, trunk injection of nematicides is the preferred method of control of the notorious pine wood nematode (PWN), *Bursaphelenchus xylophilus*. In this study, the efficacy of sixteen locally produced formulations of abamectin against the PWN are compared. The nematodes were exposed to varying concentrations of the formulations in vitro. Additionally, nematode populations were pre-exposed to 0.0212 and 0.212 μg/ml concentration of the tested formulations for 24 hours before being inoculated in trimmed tree branches. Despite the uniformity in the concentration of the active ingredient (1.8 %), efficacy was contrastingly different depending on the producer. Abamectin formulations evidently conformed to four distinct categories based on similarities in sublethal activity in vitro. A relatively similar trend was evident in the branch experiment albeit with significant variations. These variations may be attributed to differences in inert ingredients used by the producers.

**How Entomopathogenic Nematodes Accomplish Group Behaviors**

Lewis, Ed^1^, K. Cruzado-Gutierrez^1^, H. Erdogan^2^, G. N. Stephens^1^, D. I. Shapiro-Ilan^3^, F. Kaplan^4^ and H. Alborn^5^

^1^Department of Entomology, Plant Pathology and Nematology, University of Idaho, Moscow, ID 83844

^2^Bursa Uludağ Üniversitesi, Ziraat Fakültesi, Biyosistem Mühendisliği Böl., Bursa, Turkey

^3^USDA-ARS, SEA, SE Fruit and Tree Nut Research Unit, 21 Dunbar Road, Byron, GA 31008

^4^Pheronym, Inc., 1100 Main St, Ste 300-PL, Woodland, CA 95695

^5^USDA ARS CMAVE, 1600 S.W. 23RD Drive, Gainesville, FL 32608

## Abstract

Many nematode species have aggregated population structures that may possibly reflect the distribution of resources or different conditions within the soil. Entomopathogenic nematodes are obligate insect parasites. Their aggregation behavior has been documented and described to occur even in the absence of external stimuli. Aggregations which occur in conditions that are largely homogeneous suggest that communication among individuals serves to maintain their proximity. Many nematode species produce pheromones, which implies some level of communication amongst individuals. One result of communication that has been well-described in insects and other groups is trail-following, but this phenomenon has not been previously described to occur in any nematode species. Interactions among individuals are an essential basis of following behaviors and can have significant fitness consequences. We recorded intraspecific and interspecific interactions among three *Steinernema* species (*S. glaseri*, *S. carpocapsae*, and *S. feltiae*) in terms of trail following and discovered that following behavior is context dependent. Following behavior among conspecifics was significantly increased when the lead nematode had prior contact with host cuticle. However, trail following varied among heterospecific pairs. In general, communication that enables following behavior is a species-specific phenomenon and is enhanced when lead nematodes are excited by contact with host cuticle. This work brings together several years of collaborative work that seeks to understand the infection dynamics and population ecology of entomopathogenic nematodes in the context of biological control of crop pests.

**Profiles of *Meloidogyne Enterolobii* from Different Hosts and Comparison of Two Geographically Different Isolates**

Liang, Che-Chang^1^, B. Chinnasri^2^ and P. Chen^1^

^1^Department of Plant Pathology, College of Agriculture and Natural Resources, National Chung Hsing University, Taichung, 40227 Taiwan

^2^Department of Plant Pathology, Faculty of Agriculture, Kasetsart University, Bangkok 10900, Thailand

## Abstract

*Meloidogyne enterolobii* is considered one of the most damaging species due to its wide host range and the ability to develop and reproduce on several known root-knot resistant crops. In our root-knot nematode survey, *Meloidogyne enterolobii* is the dominant species in the guava production areas in Taiwan and is also found on several crops. To clarify whether this species has any intraspecific differences, GXG, CCC, and BTC isolates were collected from different host families and compared to a known *M. enterolobii* isolate (named THP) originally collected from Thailand based on morphological, molecular characteristics and isozyme phenotypes. The female perineal patterns, J2 de Man formula, mitochondria DNA CoII-IrRNA region RFLP profiles, and esterase phenotypes of these 4 isolates are consistent with the *M. enterolobii* species. The intraspecific variability of these 4 isolates was found in the 18S rRNA and 28S rRNA D2D3 sequences. The haplotypes of these sequences were confirmed by sequencing 3 clones from 3 batches of DNA obtained from 3 different generations. Six 18S rRNA haplotypes were found and haplotype 3 is the same as MN832683. The Taiwan CCC isolates had the same haplotype as MN832683, and the GXG and BTC isolates had haplotype 1, however, isolate THP from Thailand had distinct haplotypes 5 and 6 that were not found in other populations. Three 28S rRNA D2D3 haplotypes were found and GXG, BTC, and THP all had more than one haplotypes. However, the mtDNA CoII-IrRNA region sequences of all 4 *M. enterolobii* isolates were identical (MZ643270). The two geographically different *M. enterolobii* populations, GXG and THP were used to challenge several local crops revealing no significant differences in host response. Both isolates failed to parasite on runner peanut (*Arachis hypogaea* cv. TNG 9), *Citrus reticulate*, *Citrus tankan*, and green onion (*Allium fistulosum*). Isolate THP exhibited a slightly lower reproduction rate than GXG when assayed on the 2 pumpkin cultivars (cv. Acheng and Strongman) and the chili pepper (cv. Passion).

***Meloidogyne Enterolobii* Prevalence and Distribution in North Florida**

Liu, Chang and Z. J. Grabau

Entomology and Nematology Department, University of Florida, Gainesville, FL 32611

## Abstract

*Meloidogyne enterolobii* is an emerging tropical and subtropical pest. It has limited known distribution but can be highly damaging. It was first reported in the United States in 2004 in Florida, and has been reported in North Carolina, South Carolina and Louisiana. *M. enterolobii* has the ability to overcome the resistant genes commonly deployed in crops and is particularly damaging, which make it of particular concern for agricultural production especially for sweetpotato production. A study aimed at studying the prevalence and distribution of *M. enterolobii* on field and horticulture crops was carried out in North Florida counties since 2020. Sample resources include root and soil samples collected through field collection and samples submitted to the University of Florida Nematode Assay lab. Initial morphology diagnosis was conducted to screen for root-knot nematode positive samples. For each location, 5-10 females were picked out if root systems were available. If only soil samples were available, tomato seedlings (‘Rugters’) were transplanted into each soil sample and cultured for 3 months to obtain mature females. Root-knot nematode speciation was conducted based on mitochondrial haplotype-based identification. Samples diagnosed to be *M. enterolobii* were further confirmed with *M. enterolobii*-specific SCAR marker. Both Cytochrome oxidase II and 18S rDNA regions were sequenced for *M. enterolobii* positive samples. The survey was conducted in 17 counties in North Florida. A total of 287 samples were collected including 12 horticulture crops (sweetpotato, cabbage, okra, green bean, cowpea, lettuce, broccoli, potato, hemp, sunflower, carrot and pepper) and 5 field crops (peanut, cotton, corn rye, soybean), and 98 of these samples were positive for root-knot nematode. Four root-knot nematode species were found during the survey, including *M. incognita* (48%), *M. arenaria* (27%), *M. javanica* (8%), *M. enterolobii* (15%) and *M. arenaria* and *M. enterolobii* mixed species (2%). This survey is important in monitoring and limiting the spread of *M. enterolobii* in North Florida as well as awakening growers’ awareness of this emerging destructive pest. Follow up studies are underway in developing management strategies against *M. enterolobii* on sweetpotato.

**Reproduction of *Helicotylenchus*, *Scutellonema*, and *Tylenchorhynchus* in Commercial Soybean Fields in Paraguay**

Lopez-Nicora, Horacio^1,2^, A. Gini^2^, F. Lugo^2^, G. G. Caballero-Mairesse^2,3^ and G. A. Enciso-Maldonado^2,3^

^1^The Ohio State University, Department of Plant Pathology, Columbus, OH 43210

^2^Universidad San Carlos (USC), La Clínica Vegetal, Asunción 1421, Paraguay

^3^Centro de Desarrollo e Innovación Tecnológica (CEDIT), Hohenau, Itapúa 6290, Paraguay

## Abstract

Paraguay ranks sixth among soybean-producing countries in the world and third in soybean exports. Soybean is an important commodity for Paraguay’s economy and a major field crop based on planted area. The sub-tropical climate in Paraguay favors the occurrence and development of numerous pests and diseases. Recently, the number of commercial soybean fields affected by plant parasitic nematodes has increased. Soybean fields displaying aggregated areas with reduced plant emergence, height, and productivity (i.e., yield) are more frequently observed. The increasing numbers of soybean fields affected by nematodes in Paraguay could be related to the current agricultural system (soybean monoculture with little crop rotation) along with the edaphoclimatic conditions (loamy clay soils and warm, rainy winters). Plant parasitic nematodes of the genera *Helicotylenchus*, *Scutellonema*, and *Tylenchorhynchus* have been previously reported in soybean production areas, however, information about their reproduction in soybean fields in Paraguay is unknown. Therefore, the present study aimed to determine the reproductive factor of these nematodes in four commercial soybean fields in the districts of Jesus, Bella Vista, and Itapua Poty (Department of Itapua) and Curuguaty (Department of Canindeyu). These fields contained symptomatic areas where reduction in plant emergence, as well as stunted and chlorotic plants were observed. From each field, ten composite soil samples were collected at planting and at harvest to determine the initial (Pi) and final (Pf) population, respectively. The reproductive factor (RF) for each nematode genus was calculated using the formula RF = Pf/Pi and compared to RF = 1 using two-sided Student's t-test. *Helicotylenchus* was detected in all fields, with reproductive factor higher (*P* < 0.05) than one observed in Curuguaty and Itapua Poty. In Jesus and Bella Vista, however, *Helicotylenchus* RF was significantly lower than one. Of the four fields included in this study, *Scutellonema*, and *Tylenchorhynchus* were detected in three and two fields, respectively. *Scutellonema* was identified in the Jesus, Bella Vista and Itapua Poty fields with a RF above one. *Scutellonema* was not detected in Curuguaty. Finally, *Tylenchorhynchus* was detected in commercial soybean fields from Bella Vista and Curuguaty with RF significantly greater than one. Our results indicate that *Helicotylenchus*, *Scutellonema*, and *Tylenchorhynchus* reproduce in commercial soybean fields, and therefore can be considered possible pathogens to this crop. Moreover, the presence of symptomatic areas in these fields indicates that more research is needed to evaluate the relationship between these nematodes and soybean yield reduction. These nematodes could potentially become a more serious threat to soybean production without further action.

**Movement, Efficacy and Fungal Viability of Two Strains of *Purpureocillium Lilacinum* on *Meloidogyne Enterolobii***

Mayorga, Laura^1^, K. Medina-Ortega^2^ and J. Desaeger^1^

^1^Gulf Coast Research and Education Center, University of Florida, Dept. of Entomology and Nematology, Wimauma, FL 33598

^2^Certis Biologicals, Columbia, MD 21046

## Abstract

Root-knot nematodes (RKNs; *Meloidogyne* spp.) are widespread and known to cause considerable damage to several crops. *M. enterolobii* is a major emerging threat to agriculture in the southeastern United States. It was first reported in Florida in 2004 and is considered a highly virulent RKN species capable of breaking RKN resistance in soybean, sweet potatoes and tomatoes. *Purpureocilllium lilacinum (Paecilomyces lilacinus)* has been used as a biological control agent (BCA) of plant-parasitic nematodes for many years. There are several strains of *P. lilacinum*, the most used is PL251 which *is* commercialized as MeloCon® of which a new liquid formulation has just become available. The fungus mainly parasitizes eggs and females of root-knot and cyst nematodes, and other nematodes such as *Tylenchulus semipenetrans*, *Rotylenchulus reniformis* and *Radopholus similis*. Oftentimes, the efficacy of this BCA is related to its persistence in the soil, however, PL251 has low persistence, especially in sandy soils. In a first experiment, we tested the movement of fungal spores in the soil profile. One drench application of the PL251 at 2x106 spores/ g of soil was applied at planting to cucumber plants. Soil samples were taken at 3, 6, and 9 cm depth and then cultured in PDA to count the fungal colonies. Samples were taken weekly for 4 weeks. A higher number of spores was found in the top layer (3 cm) throughout the experiment accounting for 60 to 67% on average of the total spores per pot, followed by the middle layer (6 cm) accounting for 22 to 36% on average and the bottom layer (9 cm) accounting for 3 to 10% on average. Maximum number of spores was found in the first sampling date and spore density decreased over time. The results showed that after drenching *P. lilacinum* PL251 is mostly distributed in the top layer of the soil profile. In another ongoing study, *P. lilacinum* PL251 and strain PL11 are evaluated against *Meloidogyne enterolobii* on cucumber under greenhouse conditions. The soil used in this experiment is a local Myakka fine sand (95 % sand, < 1% organic matter). Half of the pots were filled with pasteurized (steamed) soil and the other half with natural soil. There were two application timings of the BCAs, seven days before planting and at planting. Plant heights and fungal persistence are measured weekly, and shoot and root fresh weight, root gall ratings and nematode egg counts will be taken at the end of the experiment 42 days after planting.

**Soybean Cyst Nematode Biology and its Effect on Resistance Development: What Can Modeling Teach us About Building Durable Resistance Management Plans?**

McCarville, Michael, J. Williams and J. Daum

BASF Morrisville, NC 27709

## Abstract

Plant-parasitic nematodes are a key yield limiting pest of crops around the world. Many of the most important plant-parasitic nematodes are managed through the deployment of plant resistance genes. The selection for virulence in nematode populations is a major threat to the effectiveness of resistance gene-based management. Little research has gone into resistance management modelling despite the importance of both plant-parasitic nematodes and resistance genes for their management. In this paper we report on a cyst nematode resistance management model created to explore the factors which are most important for determining the durability of resistance genes to this important family of plant-parasitic nematodes. The relative dominance of virulence expression, the level of inbreeding, and the number of generations per cropping season were the most important factors in predicting resistance gene durability. Other factors of cyst nematode biology that reduce the number of generations per season for only a subset of the population had a much smaller effect on the durability of resistance genes. These factors included delayed hatching within a season and early dormancy. The accuracy and utility of the model was tested using the soybean cyst nematode, *Heterodera glycines*, *rhg1*-mediated resistance system. The model accurately predicted the rate at which virulence to the *rhg1b* resistance gene developed in Iowa over a two-decade period. The model suggested resistance gene pyramids as the most durable management solution for soybean cyst nematode with multiple possible avenues to obtain acceptable efficacy and durability.

***Purpureocillium Lilacinum*, a Natural Fungal Agent Performace Review: Us Efficacy on Vegetable Crops**

Medina, Karla

Field Development Manager Southeast US, Certis Biologicals, 9145 Guilford Road, Ste #175, Columbia, MD 21046

## Abstract

*Purpureocillium lilacinum* is a natural soilborne fungus that feeds on plant-parasitic nematodes and is among the most used biological nematicides commercially available. It was formerly known as *Paecilomyces lilacinus*. A strong focus has been on learning more about *Purpureocillium lilacinum’s* performance in commercially viable formulations across different crops and target nematodes in organic and conventional agricultural systems. This has been in part due to the increased demand for bionematicides. One of the drivers for the increased demand for natural nematicides is the need for integrating more sustainable tools in the management of plant-parasitic nematodes. Other drivers involve increased awareness of environmental and climatic consequences on the use of certain chemicals, which has driven regulatory bodies across the globe to restrict and, in many cases, ban the use of older and toxic chemistries relied upon traditionally for nematode control. As a consequence, an increased demand for organically grown products has been trending by consumers across the world, and both retail and wholesale food industries are working with their growers to develop and commit to sustainable agricultural practices that lower the carbon footprint on the planet. MeloCon WG is the brand name for the product with active fungal spores of *P. lilacinum* strain 251 that needed to be frozen for extended shelf-life. A new liquid formulation was introduced in the market as a dispersible concentrate in 2022, a significant improvement in storage shelf life with no need to keep product frozen. Performance has been positive, and data will be presented and discussed. More research is needed to better understand how *P. lilacinum* interacts with other nematode control practices in a program and what factors need to be considered for best practices in implementing a bionematicide based on a natural fungal control agent.

**Potato Cyst Nematode in Guatemala: a Threat to the Lifestyle of Guatemalan Farmers and Consumers**

Mejia, Hans^1^, L.M. Dandurand^2^, I. Zasada^3^, A. Barrios^4^, C. Perez^4^ and L. Mendoza^4^

^1^Farmer-to-Farmer, Guatemala City, Guatemala

^2^University of Idaho, Moscow, ID 83844

^3^USDA-ARS, Corvallis, OR 97330

^4^POPYAN, various locations, Guatemala

## Abstract

The highlands of central and western Guatemala offer ideal conditions for growing potato. Guatemala is the largest producer of potato in Central America, with small-scale farmers responsible for 75% of this production. The potato industry is seriously threatened by the potato cyst nematodes (PCN) *Globodera rostochiensis* and *Globodera pallida*. Introduced into Guatemala decades ago, over the past 10-15 years farmers have noticed the impact of PCN on potato production. This is expressed as small and misshapen tubers, reduced yield, and in some cases an unmarketable crop. There are several factors at play that put the Guatemalan potato industry at risk from PCN. First, the extent of the distribution of *G. rostochiensis* and *G. pallida* is not known by farmers, private institutions, or government institutions. Additionally, the cost of having a nematode sample analyzed in Guatemala is approximately $20, a great expense to small-scale farmers due to the local economy. Second, the potato variety ‘Loman’ is primarily grown in Guatemala; this potato is susceptible to PCN. The market is dominated by ‘Loman’ with consumer acceptance of other varieties low. Third, because potato is the high value crop in a rotation, farmers usually grow potato every year. Fourth, there is a lack of access to certified seed free of PCN and other pathogens. Farmers often use seed from one field to plant another, spreading PCN to other fields and areas. Finally, there are only a few *G. rostochiensis* resistant varieties available in Guatemala with ‘Loman Roja’, ‘Soprano’, ‘Granola’ and ‘Ciklamen’ in the country. However these varieties are not widely available. There are no varieties available in Guatemala with resistance to *G. pallida*. Through one-on-one meeting with farmers in nine communities, it is evident that they are aware of the threat of PCN on their ability to produce potato. Unfortunately, lack of resistance, lack of certified seed, and costly diagnostics greatly limit the ability of crop consultants, and farmers to change this dire situation.

**Using Nematode Community-Based Models as Integration Platforms for Developing Sustainable Soil Health**

Melakeberhan, Haddish^1^ and A. Habteweld^2^

^1^Agricultural Nematology Laboratory, Department of Horticulture, Michigan State University, East Lansing, MI 48824

^2^Entomology and Nematology Department, University of Florida, Gainesville, FL 32611

## Abstract

The significance of nematodes as indicators of ecosystem disturbance, function and structure of the soil food web, nutrient cycling, and soil health is well established. Soil health has balanced biological, physiochemical, nutritional, structural, and water holding components that need to be kept in balance in order to generate desirable ecosystem services. Despite a substantial basic and applied knowledge on all soil health components as well as nematodes as indicators of soil health in response to agricultural practices, developing sustainable soil health remains challenging for several reasons. Major limitations include lack of: a) mechanisms that relate soil health indicators to specific soil health values, b) simultaneous consideration of multiple ecosystem services, and c) integration platforms for aligning the desirable ecosystem services. The goal of this workshop is to demonstrate how nematode community-based models can be used as integration platforms for understanding and identifying: i) agroecosystem suitability of the biophysicochemical process-driven soil health outcomes, ii) sustainability of the outcomes considering beneficial and non-beneficial organisms in the same environment, and iii) for the first time, connecting nematode numbers to soil specific health values. In order to facilitate discussion and participation, attendees are encouraged to familiarize themselves with the general (https://doi.org/10.3390/soilsystems5020032) and specific (https://doi.org/10.3390/soilsystems6020035) concepts in the models prior to the workshop.

**Benefits of White Clover as a Long-Term Cover Crop on Soil Health and Water Infiltration**

Mew, Justin, R. Paudel and K.-H. Wang

Department of Plant and Environmental Protection Sciences, University of Hawaii at Manoa, Honolulu, HI 96822

## Abstract

White clover (*Trifolium repens*) is a low growing, heat tolerant, perennial leguminous cover crop suitable as a ground cover for orchard crops in Hawaii. One shortfall of white clover is its slow growing habit making it easy to outcompete by weeds. The objective of this experiment was to examine if mixing fast-growing annual cover crops, buckwheat (*Fagopyrum esculentum*) and black oat (*Avena strigosa*), with white clover could help smother weeds, improve soil health and water infiltration. Two field trials conducted at Manoa, HI were superimposed on each other through a succession planting in a typical hard and firm inceptisol. Trial I was from 1 July to 1 December 2021. Trial II was from 4 January to 3 June 2022. Two treatments installed were: 1) cover crop (CC) and 2) no cover crop (Control). For CC, ‘New Zealand’ white clover, ‘Common’ buckwheat and ‘Soil Saver’ black oat were planted at 55, 66 and 77 kg seeds/ha, respectively. Control plots were maintained weed-free for 2 months using diquat dibromide herbicide. Tomato seedlings were transplanted 2 months after cover crop planting. Treatments were arranged in a randomized complete block design with 3 replications. Cover crop in Trial I was sprinkler irrigated resulting in overgrown weeds. Thus, a weed mat was used to tarp the entire Trial I area for one month to shade out the remaining cover crops and weeds. Cover crop in Trial II was regrown by drip irrigation and outcompeted weeds successfully. Soil samples collected at 2-month intervals over 5 sampling dates revealed that planting of cover crop mix increased nematode richness and diversity, abundance of omnivorous nematodes and structure index (SI), which were indications of a more complex and structured soil food web than Control (*P* ≤ 0.05). Furthermore, CC increased water infiltration (*P* ≤ 0.05). In Trial I, Canonical Correspondence Analysis (CCA) showed that water infiltration rate was most related to the abundance of bacterivorous and predatory nematodes, followed by nematode diversity and richness (first two axes explained 90.24% of variance). In Trial II, infiltration rate was most related to abundance of other soil fauna (enchytraeid worms, mites, rotifer, tardigrade etc.) followed by nematode diversity, numbers of bacterivorous nematodes and SI (first two axes explained 77.94% of variance). Microbial soil respiration was most related to abundance of omnivorous nematodes in Trial I but was also highly related to abundance of bacterivorous nematodes in Trial II. Faster infiltration rate resulted in higher volumetric soil moisture. Scatter plots of CCA showed that CC was slowly segregating from Control when transitioning from Trial I (first 5 months after planting) to Trial II (month 6 to 10 after planting). This result showed that succession planting of white clover improved not only soil health but also water infiltration and holding capacity of an inceptisol.

**Nematode Signal Peptide Enhance Nitrogen Assimilation in Plant and Nematode**

Mishra, Shova, W. Hu and P. M. DiGennaro

University of Florida, Department of Entomology and Nematology, Gainesville, FL 32611

## Abstract

C-terminally encoded peptides (CEP) are plant developmental signals that regulate plant growth and adaptive responses to nitrogen stress conditions. Currently fertilizer use in agriculture is not efficient with as little as 10-30% of applied fertilizer being captured by the plant roots. These signal peptides common to all vascular plants are also present in some plant parasitic nematodes. Thus, nematode CEP can be used as a tool to improve nitrogen use efficiency in a range of plants. We hypothesized nematode encoded CEP are involved in nitrogen uptake and allocation pathways in parasitized plants, transmitting nitrogen to the feeding sites. We screened six different species of *Meloidogyne* for the presence of putative CEP peptides at the genome level and identified approximately 60 CEP-like sequences encoded in the genomes of root-knot nematode (RKN; *Meloidogyne* spp.) species. However, the role of RKN CEP-like signals in terms of plant root phenotype and nitrogen uptake and assimilation is unknown. Exogenous application of MhCEP11 regulated plant root phenotype with reduced lateral root number in *Medicago* and inhibited primary root length in *Arabidopsis*. We demonstrated that exogenous application of plant and *M. hapla* CEP increases plant nitrogen transporter gene (NRT2.1) expression as shown by upregulation of NRT2.1 gene in root at 21 days post inoculation. Further, we incorporated a heavy labeled isotope of nitrogen in plant fertilizer to quantify the nitrogen assimilation in nematodes. We utilized high-throughput proteomics and quantified nematode nitrogen assimilation with and without the presence of exogenous *M. hapla* CEP. MhCEP11 increased nitrogen assimilation of 15N at 21 days post inoculation as measured by 15N/14N ratios and metabolic processes in nematode were significantly enhanced with MhCEP11 application suggesting a broader role of CEP in plant biology probably benefiting both nematodes and plants in capturing nitrogen.

**Evaluation of Tomato Root Exudates on Development of *Meloidogyne Incognita* and *M. Javanica* in Tomato**

Mohlala, Rachel M.^1^, A. O. Mbatyoti^1^, M. M. Slabbert^2^ and M. S. Daneel^1^

^1^ARC-Tropical and Subtropical Crops, Private Bag X11208, Nelspruit 1200, South Africa

^2^Tshwane University of Technology, Private Bag X680, Pretoria 0001, South Africa

## Abstract

Root-knot nematodes (specifically *Meloidogyne incognita* and *Meloidogyne javanica*) are a serious problem in tomato (*Solanum lycopersicum*) production where yields can be considerably reduced or in severe cases the crop can be completely destroyed. The tomato industry has been heavily reliant on class I nematicides, but due to the withdrawal of several of these products alternative control strategies have to be investigated. The efficacy of tomato root exudates in enhancing egg hatching was determined as part of a strategy in which *M. incognita* and *M. javanica* are allowed to hatch while no food/crop is available, resulting in the death of the juveniles before the actual crop is planted. Several experiments evaluated the effect of the number of root exudate applications (1, 2 or 3 times) as well as the waiting period between the last application of root exudates and planting of the crop (2, 5 and 7 weeks). Applications were done one week apart. Results indicated one root exudate application is probably sufficient but a 5-week waiting period is not long enough. The ideal waiting period was subsequently evaluated by determining the longevity of *M. incognita* and *M. javanica* J2 juveniles. Differently aged (1, 7, 14, 21 and 28 days old) J2 juveniles were inoculated on tomato seedlings and recovery rate was determined. Results for both *M. incognita* and *M. javanica* indicated that longevity decreased significantly with older juveniles. However, a low percentage survival was still observed with 28 day old juveniles which could explain the results of the root exudate trials.

**Incorporating Plant Litter Promotes Basal Nematodes and Distinct Microbial Communities in a Cultivated Organic Soil**

Mosdossy, Krisztina^1^, J. Whalen^1^, C. Kallenbach and B. Mimee^2^

^1^McGill University, Dept. of Natural Resource Sciences Department, Montréal, QC, Canada

^2^Agriculture and Agri-Food Canada, Research and Development Centre, St-Jean-sur-Richelieu, QC, Canada

## Abstract

Organic soils are ideal for growing vegetable crops, but are threatened by erosion and soil subsidence. Adding ligneous plant litter is expected to mitigate these losses. However, their relatively low nitrogen (N) content could affect microbial populations that govern N cycling. Furthermore, microbivorous nematodes could shift from nematodes that dominate in resource-poor environments to resource-rich environments. Therefore, multiple trophic levels in the micro-food web are at risk of changing when plant litter is added to cultivated organic soils, which has consequences to N fluxes. Our objective was to assess the change in nematode and microbial communities in a cultivated organic soil after applying ligneous plant litter to the soil. We hypothesized that incorporating litter will increase resource availability, and thus create distinct microbial communities, and increase soil nitrate and Rhabditidae (cp-1) nematodes. We tested the hypotheses in a greenhouse experiment with five litter treatments × two placements and used lettuce as a model crop. We tested four litters: tamarack (lignin:N = 338.9), white ash (lignin:N =131.7), willow (lignin:N = 64.7), and miscanthus grass (lignin:N=126.1). We determined the structure of bacterial, fungal and nematode communities from a metabarcoding analysis that targeted the 16S/ITS/18S rRNA genes. The ratio of the relative abundance of Cephalobidae (cp-2) / Rhabditidae (cp-1) was 5 times higher when plant litter was incorporated compared to when it was place on top (5.24 vs. 0.99). Incorporated larch decreased nematode diversity (ANOVA, *p* < 0.001). Litter placement and type explained ~20% of the variation of nematode diversity and composition (PERMANOVA, *p* < 0.001). Bacteria communities were similar across litter types when litter was on the soil surface, but distinct communities emerged between litter types when litter was incorporated into the soil (PERMANOVA, *p* < 0.001). Fungi communities, on the other hand, were distinct across litter type both when plant litter was on top or incorporated into the soil (PERMANOVA, *p* < 0.001). Soil nitrate was higher when litter was incorporated (*p* < 0.001) but lettuce biomass was unaffected by litter inputs. Samples were analyzed at a single time point (6 wks), which limits interpretability of N availability through time. Nonetheless, these results suggest that plant litter type and placement indeed alter the micro-food web. Cephalobidae are indicators of low resource availability, and thus fewer resources may be available when plant litter is incorporated. This compliments the decrease in nematode diversity when larch was incorporated, but is contradictory to high soil nitrate availability compared to when litter was on top. The effects of litter placement and type are also apparent in microbial communities. These results suggest a change in N flux, which will help inform whether plant litter is useful when managing cultivated organic soils.

**Management Potential of *Heterohabditis Indica* Against Queensland Long-Horned Beetle**

Myers, Roxana^1^, C. Mello^1^, C. Sylva^1^ and S. Chun^2^

^1^USDA-ARS, Daniel K. Inouye U.S. Pacific Basin Agricultural Research Center, Hilo, HI 96720

^2^State of Hawaii Department of Agriculture, Plant Pest Control Branch, Hilo, HI 97620

## Abstract

Queensland Long-horned Beetle, *Acalolepta aesthetica*, is a large cerambycid beetle reaching lengths of up to 4.5 cm. Larvae attack healthy trees causing extensive structural damage by feeding and tunneling though the trunk and branches. Girdling of the trunk, sawdust-like frass, and sap oozing from the oviposition sites are key symptoms of an infestation by this invasive pest. Round exit holes approximately 1.25 cm in size, branch dieback, and branch dropping are also observed. Confirmed hosts include citrus, avocado, cacao, breadfruit, and kukui. No known control methods currently exist for *A. aesthetica*. Prevention and destruction of infested material through chipping in place are the only recommendations. A local isolate of *Heterorhabditis indica* was explored as a potential biological control agent against *A. aesthetica* larvae. Preliminary laboratory bioassays demonstrated that larvae and pre-pupae were good hosts of the nematode with 200,000 – 400,000 IJs recovered from infected cadavers. Inoculation of *H. indica* into tunnels of *A. aesthetica*-infested kukui logs resulted in larval mortality of 18 – 46%. No differences were observed when comparing pluronic gel and water as carriers for the nematode suspension. When evaluating *H. indica* and the entomopathogenic fungus *Beauveria bassiana* in similar trials using *A. aesthetica*-infested logs, nematodes caused mortality of 35 - 52% of larvae whereas 11 - 18% of larvae were killed by *B. bassiana*. Combining the two pathogens at half the inoculum rate caused larval mortality of 29 - 54%. Further research will include inoculation of *H. indica* into oviposition and exit holes in field-grown trees with natural *A. aesthetic*a infestations.

**A Survey of Plant-Parasitic Nematodes Associated with Hop (*Humulus Lupulus*) and Hemp (*Cannabis Sativa*) in the Pacific Northwest**

Núñez-Rodríguez, Lester Antonio^1^, C. Wram^2^, A. Peetz^2^, D. Gent^1,3^, H. Rivedal^3^, C. Ocamb^1^ and I. Zasada^2^

^1^Department of Botany and Plant Pathology, Oregon State University, Corvallis, OR 97331

^2^USDA-ARS Horticultural Crops Disease and Pest Management Research Unit, Corvallis, OR 97331

^3^USDA-ARS Forage Seed and Cereal Research Unit, Corvallis, OR 97331

## Abstract

Hop (*Humulus lupulus*) and hemp (*Cannabis sativa*) are two economically important crops in the Pacific Northwest (PNW). In 2021, the value of the production of these crops together was $912 million. Washington and Oregon are two of the most important states for both hop and hemp production. Currently, there is little information about the occurrence, distribution, and densities of plant-parasitic nematodes (PPN) in these crops. In fall 2021, a three-year survey was started in the PNW to identify the PPN associated with hop and hemp. In the first year, 28 hop fields from Washington and 25 hemp fields (9 from Washington and 16 from Oregon) were sampled. Two composite soil samples were collected in each hop field and one to two composite soil samples (depending on the field size) from each hemp field. Seven different PPN were identified from soil sampled from hop fields. Second stage juveniles (J2) of the hop cyst nematode, *Heterodera humuli*, were present in 75% of samples with an average of 47 J2/250g of soil. *Heterodera humuli* cysts were found in 98% of samples with a maximum density of 550 cysts/100g of dried soil. Other PPN commonly found in hop were dagger (*Xiphinema* sp.) and stunt nematodes with >50% occurrence. Three populations of stunt nematodes (one with males and two with no males) were selected for DNA extraction and sequencing of the *ITS1*-5.8*S*-*ITS2* region. The phylogenetic analysis placed the populations without males in a clade with *Tylenchorhynchus clarus* (99.8% identity with NCBI GenBank Accession KJ461575). Meanwhile, the population with males was placed in a clade with *Bitylenchus hispaniensis* (90.6% identity with NCBI GenBank Accession MZ725020). Seven different PPN were identified in soil collected from hemp fields as well. *Pratylenchus* was the most frequently encountered PPN in hemp, being found in 47% of the samples with an average of 36 nematodes/250 g of soil. Two populations of *Pratylenchus* sp. from Oregon were identified as *P. penetrans* using the species-specific primers β14Ppf1/β14Ppr1. Our preliminary results indicate that a diversity of PPN are widely prevalent in Washington hop yards. This is the first time that *P. penetrans* has been reported associated with hemp in the PNW. Future research should focus on determining the pathogenicity of PPN to hop and hemp, and the development of management strategies.

**Integrated Nematode Management in Florida Strawberry – Opportunities and Limitations**

Oliveira, Clemen and J. Desaeger

Department of Entomology and Nematology, Gulf Coast Research and Education Center, University of Florida, Wimauma, FL 33598

## Abstract

Integrated nematode management requires a thorough understanding of the complexities of crop-nematode interactions. In Florida, plant-parasitic nematodes are one of the main pest problems in strawberry fields, so nematode management is extremely important. Strawberry is grown as an annual crop in Florida with transplants being grown out-of-state, mostly in nurseries in Canada, California, and North Carolina. Nematodes are often brought into Florida fields with those transplants, but the distribution, interaction with other pathogens, and management options are still not fully understood. To address these gaps, we performed a four-year survey to investigate what plant-parasitic nematodes are being introduced in Florida strawberry fields with the transplants. This revealed that the northern lesion nematode (*Pratylenchus penetrans*) and the northern root-knot nematode (*Meloidogyne hapla*) are being introduced to Florida via Canadian transplants. In one case, an *Aphelenchoides bessey*i infection was traced back to a nursery in North Carolina. We also assessed the distribution of plant-parasitic nematodes (PPN) in Florida strawberry fields, and a four-year field survey revealed that sting nematodes (*Belonolaimus longicaudatus*), stubby root nematodes (*Nanidorus minor*), *P. penetrans*, and *M. hapla* are the most common PPNs in strawberry grower’s fields. These results suggest that the introduced nematodes are surviving in Florida fields. To reduce the introduction of nematodes with strawberry transplants, a thermotherapy technique using steam, which was developed for pathogen control, was used before planting transplants in the field. This technique could be particularly useful for the growing acreage of organic strawberries in Florida. The effect of steaming transplants prior to planting was evaluated in an organic field with low nematode pressure, and no negative effect on plant growth and yield was noted for seven common strawberry cultivars. In terms of crop loss, sting nematode is the most important nematode in Florida strawberries, especially in organic fields where soil fumigation is not an option. Seven commercial strawberry cultivars were evaluated for host potential to sting nematode in the greenhouse. All cultivars were good hosts having reproduction factors from 13.4 to 23.8. When planted in a sting-nematode infested field, yields for all cultivars increased in beds that were fumigated with C-35 (chloropicrin + 1,3-D). Many nematodes and pathogens occur simultaneously in Florida strawberry fields, but no information is available on possible interactions between them. An important strawberry pathogen that is introduced with transplants is *Phytophthora cactorum*.

(*Continued*)

## Abstract (Continued)

We investigated its interaction with a native nematode, *B. longicaudatus* on seven strawberry cultivars, and demonstrated there is an antagonistic interaction, where *P. cactorum* suppresses *B. longicaudatus*. Integrated nematode management in Florida strawberries is complicated and data-intensive with many different variables, including the presence of multiple nematodes and pathogens in fields, nematodes coming in with transplants from outside, and limited chemical options. Our studies provide useful information that will help to develop future INM programs for Florida strawberries.

**Response of *Pratylenchus Penetrans* and *Verticillium Dahliae* to Manure-Based Amendments with Different C:n Ratios**

Parrado, Luisa, E. Cole and M. Quintanilla

Michigan State University, Department of Entomology, East Lansing, MI

## Abstract

Michigan ranks 8th in the nation with more than 44,000 potato acres, and a $1.24 billion economic value. Plant-parasitic nematodes cause millions of dollars in economic loss each year to the state’s $104.7 billion food and agriculture industry. In potato production, the root-lesion nematode *Pratylenchus penetrans* can interact with the wilt-inducing fungi *Verticillium dahliae* causing a disease known as Potato Early Die (PED). This disease causes damage to the root system and reduces photosynthesis rate, negatively impacting crop productivity by 30%-50%. As a major problem in potato production, intensive management practices are required and include soil fumigation and regular applications of fungicides and nematicides. However, such practices can negatively impact soil health and quality, highlighting the need for new sustainable management alternatives for PED. Manure-based amendments have been widely used to improve soil quality; however, such amendments can also suppress a variety of soil-borne pathogens. The effectiveness of manure-based amendments to suppress soil-borne pathogens is thought to be dependent on a variety of factors including carbon and nitrogen ratio (C:N). Therefore, the main goal of this study was to investigate the response of *P. penetrans* and *V. dahliae* to manure-based amendments with different C:N ratios under field and laboratory conditions. Firstly, to determine the direct effect on *P. penetrans* and *V. dahliae*, extracts of six different compost and manures were prepared. Mixed stages of *P. penetrans* were exposed to different concentrations of the amendments extracts for 7 days on 12-well plates. Daily observations of nematode motility were recorded in order to establish mortality. As for *V. dahliae*, PDA media amended with different concentrations of the amendment’s extracts were prepared, and either single *V. dahliae* miscrosclerotia or a plug of mycelia were placed in the middle of the petri dish. Germination of microsclerotia, radial growth, and production of microsclerotia was recorded daily for 15 days. Each amendment extract concentration had 4 replicates and the experiment was repeated twice. A field trial was additionally established at a commercial potato field with a high-pressure history of *P. penetrans* and *V. dahliae* in Three Rivers, MI. The potato variety cv. ‘Russet Norkotah’ was used for this experiment given its high susceptibility to

*V. dahliae*. Measurements regarding *P. penetrans* incidence in soil and roots, *V. dahliae* incidence in soil and stems, crop productivity and tuber quality were recorded throughout the growing season of 2021.

(*Continued*)

## Abstract (Continued)

Results from the field experiments allow us to conclude that the different compost and manures evaluated have different effects on *P. penetrans* populations. For instance, application of poultry compost reduces *P. penetrans* populations in soil, when compared to Compost A + Gypsum, where populations increased. As for the laboratory experiments, they will be concluded by August. Results from these experiments are significant to the potato industry by providing sustainable management alternatives that successfully aid to reduce the impact of PED.

**Cover Crop Implications on the Soil Faunal Community**

Pate, Shelly N.^1^, H. M. Kelly^1^, and L. A. Schumacher^2^

^1^Entomology and Plant Pathology Department, University of Tennessee, 370 Plant Biotechnology, Knoxville, TN 37996, and 605 Airways Blvd, Jackson, TN 38301

^2^USDA, Agricultural Research Service, Crop Genetics Research Unit, 605 Airways Blvd, Jackson, TN 38301

## Abstract

Many growers plant cover crops ahead of planting soybean. Factors that accompany cover crop/soybean management can produce unexpected effects on agronomic traits, nematodes (plant parasites, fungivores, bacterivores, omnivores, predators), and other soil fauna (rotifers, tardigrades, mites, oligochaetes, Collembola). During the 2020-2021 season, a randomized factorial trial was conducted in Madison County, TN consisting of three factors: 1) cover crop mixes (fallow, five-way mix without *Brassica* spp., and six-way mix with *Brassica* spp.); 2) burndown timing (three weeks before planting and at planting); and 3) seed treatments (fungicide-only, insecticide-only, fungicide/insecticide, and fungicide/insecticide/nematicide). Soil samples were taken at four time points. Seedling emergence, biomass, yield, and the soil faunal community were analyzed. Early burndown of cover crop treatments led to significant reductions in the reproduction factors of soybean cyst nematode, fungivores, and bacterivores. The only significant two-way interaction consisted of burndown timing and seed treatment, which showed increased reproduction of Collembola in early burndown plots with the 3-way seed treatment. Fallow treatments had greater emergence and yield than both cover crop treatments. These emergence and yield differences may be due to poor seed-to-soil contact resulting from one planting depth used across all treatments. Combination seed treatments outperformed stand-alone treatments in terms of yield. Overall, to obtain optimum yield and reduce soybean cyst nematode populations, growers should utilize an early burndown timing with a combination seed treatment.

**Expediting Soil Health Improvement Effects of Sorghum/ Sorghum-Sudangrass Hybrids Through Low-Till Practice in a Rotylenchulus Reniformis Infested Soil**

Paudel, Roshan and K.-H Wang

Department of Plant and Environmental Protection Sciences, University of Hawaii at Manoa, Honolulu, HI 96822

## Abstract

Growing sorghum and Sorghum-sudangrass hybrids (SSgH) as cover crop followed by a no-till practice have a great potential to improve soil health by adding soil carbon, improving water conservation and suppressing soil-borne pathogens through the allelopathic effect. However, practicing no-till cover cropping with SSgH to improve soil health often takes years. Seven SSgH varieties were examined for their efficiency in 1) suppressing *Rotylenchulus reniformis* through allelopathic effect, 2) soil water conservation, and 3) overall soil health improvement in a tropical Oxisol. A greenhouse dibble tube bioassay was conducted to compare the allelopathic effects of tissue from 7 SSgH varieties to sunn hemp (*Crotalaria juncea*) against *R. reniformis* in a sterile sand: soil mix. An unamended control was included. The sand: soil mix was amended with macerated tissues of the 7 SSgH collected from 1-,2-, and 3-month-old plant tissues at 1% (dw/ dw).A ‘California Blackeye’ cowpea (*Vigna unguiculata*) seedling was planted and inoculated with 50 juveniles of *R. reniformis* per tube for bioassay. Number of females per gram of root were examined at one month after inoculation through root staining. Energy sorghum ‘NX-D-61/NX2’ and SSgH ‘Latte’ and ‘542’ had the lowest *R. reniformis* females regardless of the age of the tissues. However, other SSgH varieties lost their allelopathic effect at 2- and 3- month old though their 1-month old tissues were suppressive to *R. reniformis*. A field trial was conducted at Poamoho Experiment Station where 7 SSgH varieties were grown for 3 months and terminated using a flail mower followed by growing eggplant (*Solanum melongen*^a^) for 5 months. SSgH biomass was incorporated into the soil following a low-till practice where cover crop residues were shallowly tilled (10 cm deep) in narrow strips (20 cm wide) using a two-tine tiller. While SSgH ‘542’and ‘NX2’ produced the highest biomass, low-till practice of ‘NX2’, ‘LA’, and ‘Piper’ resulted in fastest soil water infiltration rate compared to a bare ground (BG) till control. Soil carbon and soil microbial respiration were also highest in ‘NX2’ throughout the SSgH-eggplant cycle. The abundance of fungivorous and omnivorous nematodes was highest in ‘Piper’, ‘BK’, and ‘NX2’, but only ‘Piper’ reduced *R. reniformis* abundance compared to BG. Total microbial biomass estimated by phospholipid fatty acid (PLFA) analysis was only increased by ‘NX2’ 2 months after growing SSgH, though this effect dissipated towards the end of the eggplant crop. Multivariate

(*Continued*)

## Abstract (Continued)

analysis among all parameters showed positive relationships among soil health indicators: structure index, soil microbial respiration rates, soil carbon content, nematode richness, diversity, and water infiltration rate. Eggplant yield was positively related to volumetric soil moisture, abundance of bacterivorous nematodes, total microbial biomass, and arbuscular mycorrhizal fungi biomass. Overall findings revealed that low-till practice of ‘NX2’ and ‘Piper’ cover crops could improve soil health and suppress reniform nematode within one cropping cycle of eggplant, respectively.

**Effects of Organic and Inorganic Compounds on Hatching of *Pratylenchus* Spp**.

Pennewitt, Monica and G. L. Tylka

Department of Plant Pathology, Entomology and Microbiology, Iowa State University, Ames, IA 50011

## Abstract

Root-lesion nematodes (*Pratylenchus* spp.) have a broad host range of nearly 350 plant species and are known to reduce yields of many crops. Eggs of plant-parasitic nematodes have great overwintering ability, and hatching can be induced by complex compounds including various organic acids, amino acids, and sugars released by host seeds and roots into the rhizosphere. Among the compounds exuded from corn (*Zea mays*), which is a host of several *Pratylenchus* species, are three common organic acids: citric, malic, and succinic. Because host plants naturally exude these chemicals into the soil during the growing season, it is hypothesized that one or more may stimulate hatching. Additionally, various ionic solutions, including sulfates (Na^2^SO^4^, ZnSO^4^), nitrates (NH^4^NO^3^, KNO^3^), and chlorides (MgCl^2^, CaCl^2^, KCl), have been studied for effects on hatching of several nematode genera, but the impacts on hatching of *Pratylenchus* spp. are unknown. The objectives of this research were to determine the effects of organic and inorganic compounds on hatching of *P. penetrans* and *P. scribneri*. Nematode cultures were maintained on root explants of corn growing in Pluronic gel containing Gamborg’s-B5 with vitamins for 2 months in a dark incubator at 25°C. The growth medium containing nematodes was liquified by cooling to 4°C and then poured through stacked sieves that trapped different stages of the nematode. Eggs and second-stage juveniles (J2s) were recovered on the bottom, 20-μm-pore sieve. The J2s were separated from the eggs by placing the nematode suspension on a 22-μm-pore sieve in a glass beaker with deionized water and rotating at 50 rpm for 2 hours on a platform shaker. Approximately 300 μl of egg suspension were placed into individual wells of 6-well tissue culture plates containing test solutions and incubated at 25°C in darkness for 14 days. Every 2 days the sieves were transferred to a new well with 3 ml of new test solution and the hatched juveniles present in the previous well were counted. Due to the unknown concentration of organic acids in corn root exudates, concentrations of 1, 2.5, and 5 M were arbitrarily chosen for the experiments. Solutions of Na^2^SO^4^, NH^4^NO^3^, KNO^3^, MgCl^2^, CaCl^2^, and KCl were tested at 5.5 mM, and 5.6 mM ZnSO^4^, a hatch stimulant of soybean cyst nematode (*Heterodera glycines*), also was studied. Control solutions included 100 ppm of the nematicide abamectin and sterile deionized water. Malic, citric, and succinic acids had no significant effect on hatching of either species. The greatest amount of hatch for *P. scribneri* was in water and MgCl^2^; hatching of *P. penetrans* was greatest in NH^4^NO^3^ and CaCl^2^. There were no significant differences in hatching among any of the other inorganic compounds. Abamectin did not consistently reduce hatch across all experiments. Future work will assess the effects of corn root exudates from plants of varying ages on *Pratylenchus* hatching.

**Assessment of Breeding Lines for Resistance to Soybean Cyst Nematode (*Heterodera Glycines*) and their Copy Number Variation at *Rhg1* Locus**

Poudel, Dinesh and G. Yan

North Dakota State University, Department of Plant Pathology, Fargo, ND 58108

## Abstract

Soybean cyst nematode (SCN, *Heterodera glycines*) is a devastating pathogen affecting soybean (*Glycine max* L.) production worldwide. Host resistance is one of the primary practices used to manage SCN. The *Rhg1* locus, a tandem repeat of a 31.2 kb unit on chromosome 18 of the soybean genome, contributes to the strong and effective SCN resistance, and is widely used in most commercial cultivars. Previous studies have shown large variation in the copy number of the repeat and sequence of the individual repeat units among different soybean accessions. Both copy number variation and sequence variation at *Rhg1* locus are important for determining the level of resistance to SCN, the former having a dominant role. To evaluate host resistance to SCN, 24 soybean breeding lines were screened for their resistance reactions to two prevalent SCN populations from North Dakota, HG type 2.5.7 and HG type 7 under controlled growth chamber conditions by inoculating each plant with 2,000 SCN eggs and enumerating the number of white females on the roots and in the soil 30 days after inoculation. Based on the Female Index (FI = average number of females on a tested line/average number of females in Barnes, a susceptible soybean check × 100), the resistance phenotype of each breeding line was classified as resistant (FI: < 10%), moderately resistant (FI: 10-30%), moderately susceptible (FI: 30-60%), or susceptible (FI: > 60%). To investigate the copy number variation among the breeding lines at the *Rhg1* locus and the association with resistance phenotype, a SYBR Green-based quantitative real-time PCR (qPCR) assay was adopted and validated using 12 soybean accessions with known copy numbers ranging from 1 to 10. The qPCR was carried out with the genomic DNA samples extracted from young leaf tissues of the soybean plants grown under controlled conditions for 10 days. A heat-shock protein gene (*hsp*) was used as an internal control. The 2^−ΔΔCT^ method was used to quantify the copy number of the repeat based on the reference check, Williams 82 with a single copy. Among 24 breeding lines, for HG type 2.5.7, we found two moderately resistant lines, ND18-17666 and ND19-18020(GT) with FI 29.9% and 25.9%, respectively. For HG type 7, only one breeding line, ND18-17666 was found to be moderately resistant with FI 22.5%. Results of qPCR revealed that the copy numbers of the repeat among the breeding lines ranged from 1 to 11. Both ND18-17666 and ND19-18020(GT) had 11 copies of the repeat, while the remaining breeding

(*Continued*)

## Abstract (Continued)

lines had a single copy of the *Rhg1* repeat, showing a strong association with the resistance phenotypes. Further validation of the copy number will be performed by next-generation sequencing. The copy number assessment will facilitate screening of soybean germplasm and breeding lines for breeding programs to efficiently develop new cultivars with resistance to SCN.

**Losses of Plasticity Convergently Targeted a Morphology-Influencing Gene During Diplogastrid Stoma Evolution**

Ragsdale, Erik J. and N. A. Levis

Department of Biology, Indiana University, Bloomington, IN 47405

## Abstract

Family Diplogastridae has undergone rapid rates of evolution in its stomatal morphologies, enabling a wide range of feeding habits compared with strictly microbivorous outgroups. Previous work showed this burst of evolutionary rates to correspond with the presence of discrete developmental plasticity, or polyphenism - that is, the ability to produce multiple morphs from a single genotype - in feeding morphologies. Moreover, feeding morphologies were shown to evolve even faster following the loss of polyphenism, an event that happened multiple times in Diplogastridae. To study the molecular underpinnings of this evolutionary history, we integrated molecular evolutionary analysis of polyphenism-associated genes with functional-genetic assays by DNA editing (CRISPR/Cas9). We first inferred an ancestral, “core” set of polyphenism-biased genes for the diplogastrid genus *Pristionchus* (“shark-tooth” nematodes) by comparing the transcriptomes of lines we experimentally fixed for either morph in two disparate species. We then reconstructed the evolution of these genes across Diplogastridae, identifying a limited set of genes with similar evolutionary signatures across lineages that lost the polyphenism. To validate the functional relevance of one of these genes, we created knockout mutants for the gene in *P. pacificus*. Mutations resulted in aberrations in stomatal morphology, indicating the gene’s influence on the polyphenic trait. Thus, genes highlighted by macroevolutionary comparisons of a polyphenism point to a potential mechanism for change in a rapidly evolving nematode morphology.

**The Demonstrated Need for Integrated Management of Sting Nematode In Newly Planted Citrus Trees in Florida**

Regmi, Homan^1^, F. El-Borai^2^, S. Y. Wu^3^, J. Desaeger^2^ and L. Duncan^1^

^1^Entomology and Nematology Department, University of Florida, Citrus Research and Education Center (CREC), 33850

^2^Entomology and Nematology Department, University of Florida, Gulf Coast Research and Education Center (GCREC), 33598

^3^Fujian Agriculture and Forestry University, Fujian, China

## Abstract

Huanglongbing (HLB) is a devastating, phloem-limited bacterial disease of citrus that is a major cause of citrus decline in many parts of the world. Annual citrus production in Florida today is less than a quarter of that in 2005 when HLB was introduced in the state. Subsequent large-scale replanting of unproductive orchards exacerbated damage by *Belonolaimus longicaudatus*, which is especially virulent to young trees. The efficacy of six nematicides against the sting nematode was evaluated in a commercial orchard of 15-month-old Valencia orange trees on Kuharske rootstock. All trees were symptomatic for HLB. Trees were treated twelve times during three years, twice in spring with either fluensulfone, fluopyram, fluazaindolizine, or an unregistered experimental compound, and twice in autumn. These four compounds were paired and alternated each season. Two additional treatments were oxamyl (twice in each of spring and autumn) and aldicarb (applied once each spring). The experimental design was randomized complete block with eight replications and 4-tree plots for each of the seven treatments. Nematodes and fibrous roots were measured in summer and winter each year. Compared to untreated trees, the growth of the tree trunks and the mass of fibrous roots during 3 years was greater for trees treated with all nematicides than for untreated trees. Oxamyl was the best-performing nematicide with fibrous root density as much as 2.27-fold that of untreated trees and 36% greater trunk growth. The cumulative nematode densities for each plot during three years were inversely related to trunk girth, fibrous root density and fruit yield. Analysis of the year-three yield as affected both by the trunk girth prior to treatments and the cumulative sting nematode populations during the trial showed that trees produced more fruit in response to nematode management, and the magnitude of the increase depended on the initial size (trunk girth) of the tree. The initially largest trees produced 6.5 times as much fruit as the smallest. High cumulative sting nematode density reduced the yield of large trees 39% and small trees 72%. Despite the improved growth and yield in treated trees, the harvested fruit from the 4.5-year-old HLB-affected trees averaged just 35, 90-lb. boxes per acre in 2022. Accordingly, we are investigating the use of individual protective tree covers (IPCs) to determine if managing root herbivores such as sting nematode is more profitable when newly planted trees are also protected from the insect vectors of HLB. We anticipate that IPCs will confer greater tolerance to nematodes, making additional management tactics more profitable.

**Two-Way Plant-Mediated Interactions Between a Plant Parasitic Nematode and a Foliar Herbivore Arthropod**

Ripa, Lucas and E. Lewis

University of Idaho, Department of Entomology, Plant Pathology and Nematology, Moscow, ID 83844

## Abstract

Interactions between belowground and aboveground heterotrophic communities that have no direct physical contact can be connected through the plant as the mediator of these interactions. Herbivores use different types of cues to find suitable host plants, and plants respond to the attack of herbivores by producing a suite of defensive compounds which can affect the choice and performance of herbivores. The aim of this study was to explore two-way plant-mediated interactions between two herbivores; the belowground plant-parasitic root knot nematode (RKN) *Meloidogyne incognita*, and the two-spotted spider mite, *Tetranychus urticae* Koch (TSSM) which is an aboveground folivore. Two different host species were used, Lima bean (LB) (*Phaseolus lunatus* cv. Henderson) which is an optimal host for TSSM, and tomato (*Solanum lycopersicum* cv. Rutgers) which is a sub-optimal host, for TSSM. To test RKN preference, we used two-way glass olfactometers to determine the response of RKN to Lima Bean plants that were exposed to TSSM versus a non-exposed LB plant. RKN consistently infected the non-exposed plants at a significantly greater rate than the TSSM exposed plants. For the aboveground experiments, we used leaf discs and two-way olfactometers choice to conduct tests to observe the response of TSSM to RKN infected plant against a non-exposed plant, at different days post-inoculation (DPI) with RKN. Significantly more TSSM were found on LB plants that were infected with RKN compared to non-infected plants only at 25 DPI. On tomato plants, TSSM significantly preferred RKN infected plants at 1 DPI. We also tested the effect of the inoculation (1 DPI) of soil-dwelling entomopathogenic nematodes (EPNs), *Steinernema carpocapsae* on tomato plants, and found that TSSM preferred the EPN inoculated plants compared to non-inoculated tomato. We carried out a non-choice performance test for TSSM in both LB and tomato plants, to determine the performance of the TSSM on plants inoculated with RKN versus clean plants, and observed no effect of RKN exposure on TSSM performance.

**Optimizing Survey and Detection Methods for Plant Parasitic Nematodes, Including *Anguina* Spp. in Grasses Grown for Seed in Oregon**

Rivedal, Hannah M.^1^, E. Braithwaite^2^, A. Kowalewski^2^, R. Starchvick^1^, T. Temple^1^, N. Wade^3^, A. Peetz^4^ and I. Zasada^4^

^1^USDA-ARS FSCRU, Corvallis, OR 97331

^2^Oregon State University, Dept. of Horticulture, Corvallis, OR 97331

^3^Oregon State University, Dept. of Botany and Plant Pathology, Corvallis, OR 97331

^4^USDA-ARS HCRU, Corvallis, OR 97331

## Abstract

Oregon’s grass for seed industry is valued at $500 million and produces 70% of the world’s grass seed. Oregon specializes in production of grasses for forage, including annual ryegrass (*Lolium multiflorum*) and orchard grass (*Dactylis glomerata*) seed. These grass species are hosts of *Anguina funesta* and *Anguina* spp., respectively, which cause yield-limiting seed galls and can vector *Rathayibacter* spp., toxic bacteria to grazing livestock. Export countries have strict phytosanitary regulations causing rejection of seed lots infested with the vector nematodes, a costly expense for Oregon growers. Current best practices for determining *Anguina* infestations in grass seed fields focus on post-harvest seed evaluation for nematode presence. These methods do not allow a grower to determine a field’s risk of *Anguina* spp. infection before harvest. Methods to evaluate fields before harvest, could reduce the amount of contaminated seed prepared for shipment overseas and lead to fewer seed lot rejections. In addition, a greater understanding of the biology of these and other plant-parasitic nematodes (PPN) in the grass seed system would allow for research on management of PPN. In this study, we have initiated a 2-year survey to evaluate timing, collections, and detection methods, to generate new recommendations for *Anguina* spp. and PPN survey best practices. In spring of 2022, 21 annual ryegrass and orchard grass fields in Oregon’s Willamette Valley were selected for survey. Fields were sampled four times per year: tillering (soil and tiller collections, March), initial flowering (tiller collection, May), harvest (seed collection, June-July), and germination (soil and tiller collection, October). At each survey timepoint, six 100m transects were walked, collecting 10 tiller, seed, or soil cores every 10m. Nematodes were extracted from soil using Baermann funnels for 5 days, from tillers under intermittent mist for 5 days, and from seeds by soaking in water for 2 days before evaluation under a dissecting scope for the presence of PPN. In March, *Anguina* spp. were not readily detected from soil, though five other genera of PPN were detected including significant occurrences of *Pratylenchus* (avg. 68 nematodes/50g soil) and *Meloidoigyne* (avg. 78 second-stage juveniles/50g soil). Extraction of *Anguina* spp. from tillers in March led to the recovery of *Anguina* spp. in 4 fields, with transects ranging from 1 to 40 *Anguina* spp./g of tiller. In May, *Anguina* spp. were recovered from 3 fields, with less than 1 *Anguina* spp/g of tiller on average per transect. One tiller per transect was saved for molecular evaluation with *Anguina* species-specific PCR primers. Field surveys and evaluations will continue into 2023, leading to a greater understanding of *Anguina* spp. and PPN distribution in Oregon grass for seed fields.

**Soybean Cyst Nematode Control and Plant Systemic Resistance Response to Seed-Applied Sdhi Fungicides**

Rocha, Leonardo F., J. P. Bond and A. M. Fakhoury

School of Agricultural Sciences, Southern Illinois University, Carbondale, IL 62901

## Abstract

The soybean cyst nematode (SCN) (*Heterodera glycines*) is a significant yield-limiting factor in soybean production in the Midwestern US. Several management practices are implemented to mitigate yield losses caused by SCN, including using SDHI (succinate dehydrogenase inhibitors) fungicides delivered as seed treatments. The primary seed treatment chemistries currently used to manage SCN are abamectin, fluopyram, pydiflumetofen, and tioxazafen. Fluopyram and pydiflumetofen, introduced in the last decade, are systemic succinate dehydrogenase inhibitors (SDHI) fungicides, protecting soybean seedlings against soil-borne diseases, including SCN and other plant-parasitic nematodes. Like with fungi, succinate dehydrogenase inhibition (SDHI) is the likely target for fluopyram and pydiflumetofen in plant-parasitic nematodes. In the mitochondrial matrix, SDH catalyzes the oxidation of succinate to fumarate, transferring electrons to ubiquinone without pumping protons across the mitochondrial inner membrane. These SDHI chemistries, classified as group 7 by the fungicide resistance action committee (FRAC), act by strongly binding to ubiquinone-binding sites (Qp) (competitive inhibition) in the succinate dehydrogenase complex, which is composed of four subunits (SdhA, SdhB, SdhC, and SdhD). Fluopyram and pydiflumetofen are reported to have activity against major soybean diseases, including sudden death syndrome (*Fusarium virguliforme*) and SCN. Fluopyram and pydiflumetofen are SDHI fungicides with similar chemical structures, but fluopyram appears to have a broader range of activity against several fungal pathogens in field studies and a label recommendation for early-season Septoria brown spot (*Septoria glycines*). The fact that fluopyram, a seed-applied fungicide, has activity against a broad range of nematodes and fungal pathogens, including a soybean foliar pathogen (*S. glycines*), signals a potential connection to systemic resistance induction in plant hosts. The objectives of this study were to *i*) determine the effect of seed-applied fluopyram and pydiflumetofen on soybean growth and development; *ii*) explore how fluopyram and pydiflumetofen affect SCN penetration, reproduction, and fecundity; *iii*) use next-generation sequencing technologies (RNA-seq) to identify transcriptomic shifts in soybean gene expression profiles in response to fluopyram and pydiflumetofen. Cyst counts in untreated control and pydiflumetofen treated plants were 3.44 and 3.59 times higher than fluopyram, respectively, while egg counts were 8.25 and 7.06 times higher in the control and pydiflumetofen treatment. A set of bioinformatics tools were used to identify transcriptomic shifts and

(*Continued*)

## Abstract (Continued)

metabolic pathways in soybean after seed treatment with SDHI fungicides. Samples were collected from treatments (control, fluopyram, and pydiflumetofen) at two different time points (5 and 10 days after planting (DAP)). Each sampling included four biological replicates per treatment, sequenced to a depth of 35 million reads per sample. Differentially expressed genes (DEG) were normalized and identified using DESeq2. For each time point and treatment, pairwise comparisons were executed comparing treatments to the mock control, across all four replicated libraries sequenced for each treatment. The effect of fluopyram and pydiflumetofen on a list of genes linked to soybean systemic resistance was further analyzed using a series of bioinformatics tools.

**Selection 0f a Pepper (*Capsicum Annuum*) Line with Resistance to *Meloidogyne Enterolobii***

Rutter, William B.^1^, P. A. Wadl^1^ and P. Agudelo^2^

^1^United States Vegetable Laboratory, Charleston, SC 29414

^2^Department of Plant and Environmental Sciences, Clemson University, Clemson, SC 29634

## Abstract

*Meloidogyne enterolobii* is a highly virulent species of root-knot nematode (RKN) that has been spreading across the southeastern US and has shown the ability to overcome RKN resistance in a variety of crops including pepper (*Capsicum annuum*). None of the pepper resistance genes that are known to control other tropical RKN species (i.e. *M. incognita*, *M. javanica*, and *M. arenaria*) are effective against *M. enterolobii*. New sources of pepper resistance would help farmers manage this nematode in their fields. To identify pepper germplasm with resistance to *M. enterolobii*, we screened 192 *C. annuum* plant introductions (PIs) from the USDA, GRIN germplasm collection. We included the RKN resistant line CM334 as a susceptible control, which consistently averaged 50% root system galling and 308,887 *M. enterolobii* eggs/gram of dry root across replicate screens. The majority of PIs screened had galling and reproduction levels equal to or greater than the control, with no PI showing consistent resistance across multiple experimental replicates. Through propagation of individual plants with enhanced resistance we selected a single germplasm line, PMER-2, with higher levels of resistance. In replicated greenhouse trials, PMER-2 produced 70% more root biomass by weight than controls and had significantly less galling, significantly fewer total *M. enterolobii* eggs per plant, as well as eggs per gram of root. Incorporation of the resistance identified in PMER-2 into commercial pepper varieties could provide US farmers with a useful tool to help manage *M. enterolobii*.

**Winter Cropping Systems and their Effect on the Nematode Community**

Sandoval-Ruiz, Rebeca^1^, R. Seepaul^2^, I., Small^2^ and Z. J. Grabau^1^

^1^University of Florida, Dept. of Entomology and Nematology, Gainesville, FL 32611

^2^University of Florida, North Florida Research and Education Center, Quincy, FL 32351

## Abstract

Soil health is a foundational requirement for sustainable agriculture. In recent times, there has been an increase in the search for nematode management with environmentally sustainable approaches. This implies the analysis of the nematode community, not only plant-parasitic (PPN) but also the free-living (FL) nematodes. Nematode management strategies that decrease PPN without affecting FL are desired. During winter in Florida, the soil could be left fallow, or some winter crops such as brassicas and cereals can be planted. Winter crops could improve soil health under the right crop selection and the proper management strategy. They have different inherent characteristics that could affect the nematode community. The objective of this research was to determine the effect of winter cropping systems on the nematode community. This 4-year rotation research located in Quincy, FL had a split-split plot design with depth, winter crop, and summer crop as factors. In winter, the two-year rotations were carinata-fallow, oat-carinata, and fallow-fallow. After each winter crop, the cash crops planted during summer were corn, cotton, peanut, and soybean. Soil samples were taken in spring from 2018 to 2021 to 120 cm deep. PPN genus found by morphological identification include *Rotylenchulus, Helicotylenchus*, *Meloidogyne, Mesocriconema*, *Pratylenchus*, and *Paratrichodorus*. The abundance of PPN, dominated by *Rotylenchulus*, was significantly affected by crop only during the first two sampling dates. Nematode vertical distribution was assessed from 0-30 cm and 30-120 cm. In most cases, the winter cropping system did not affect the vertical distribution. On some sampling dates, peanut shifted RN distribution deeper in the soil profile. The abundance of FL was greater in carinata than in fallow or oat, except for 2021 when no differences were seen. Bacterivores were the most abundant group of nematodes in the three cropping systems and were greater in carinata than in fallow or oat. Fungivores were significantly greater in carinata in 2019 and 2020, but no differences in the abundance of fungivores among winter crops were observed between 2018 and 2021. Rotations did not affect omnivores, but the populations tended to increase throughout the study. Predators were present in low abundances and did not have differences except in 2021 when oat had greater abundances. Nematode community indices were calculated. Hill’s N1 was consistently higher in the carinata-oat rotation system indicating a more diverse nematode community. However, the ecosystem function indices (Enrichment, Structure, Basal, and Channel indexes) and the Maturity Index showed an interaction with the summer crop planted in 2019 and 2020, and no differences were seen in 2018 and 2021. Future research on the nematode metabarcoding analysis for the cropping system will be conducted.

**Response of Root-Knot Nematode Resistant and Susceptible Soybean Genotypes to *Meloidogyne Enterolobii***

Santos Rezende, Josielle and T. T. Watson

Department of Plant Pathology & Crop Physiology, Louisiana State University Agricultural Center, Baton Rouge, LA 70803

## Abstract

*Meloidogyne enterolobii* is one of the most damaging Meloidogyne species because of its high reproduction rate and ability to overcome the resistance of important crops carrying root-knot nematode resistance genes. Soybean can be drastically damaged by plant-parasitic nematodes and species of the genus Meloidogyne are considered one of the most economically damaging nematodes of soybean worldwide. Fortunately, several soybean varieties with resistance to root-knot nematodes have been developed and utilized by farmers in root-knot nematode infested fields. However, limited data is available about the response of root-knot resistant and susceptible soybean varieties to *M. enterolobii* infection. We therefore conducted three experiments to evaluate *M. enterolobii* reproduction and gall formation on roots of three resistant and eight susceptible soybean genotypes. Different levels of *M. enterolobii* reproduction and root galling were observed in all soybean genotypes with none being resistant to this nematode. Interestingly, root-knot resistant soybean varieties were among the most susceptible genotypes to *Meloidogyne enterolobii* in all three experiments. In our study, a resistant variety had the highest egg count and gall index across all soybean genotypes inoculated with *M. enterolobii*. Our data show that root-knot resistant and susceptible soybean varieties may not hold the same root-knot host status when challenged with *M. enterolobii* infection.

**Cotton Cultivar, Nematicide, and *Rotylenchulus Reniformis* Effects on Seed Cotton Yield**

Schloemer, Claire, S. H. Graham, B. Meyer and K. S. Lawrence

Department of Entomology & Plant Pathology, Auburn University, Auburn, AL

## Abstract

The objective was to determine seed cotton yield loss due to *Rotylenchulus reniformis* and document the effect of the addition of a nematicide on seed cotton yield. The field trial was established near Belle Mina, Alabama in a field infested with *R. reniformis*. Ten cotton cultivars, including two nematode resistant cultivars, were planted with and without the nematicide aldicarb. Aldicarb application reduced the nematode eggs per gram of root by 80% across all cotton cultivars tested. The addition of aldicarb also increased seed cotton yield by 1107 kg/ha (988 lb/A), which would be estimated at 443 kilograms of lint per hectare (395 lb/A). Cotton cultivar selection did not significantly affect *R. reniformis* population density, but the highest yields in this test were recorded on the nematode resistant cultivars PHY 332 W3FE and PHY 443W3FE, which showed statistically higher yields than the other cultivars. Overall, these two reniform nematode resistant cultivars produced 1894 kg/ha (1690 lb/A) more seed cotton than the average of all the remaining cotton cultivars tested.

**Toxic Effects of the Trap Crop, *Solanum Sisymbriifolium*, on the Potato Cyst Nematode, *Globodera Pallida***

Schulz, Lindsay^1^, L. M. Dandurand^1^ and I. Popova^2^

^1^Department of Entomology, Plant Pathology, and Nematology, University of Idaho, Moscow, ID 83844

^2^Soil and Water Resources Department, University of Idaho, Moscow ID

## Abstract

The quarantined pest of potato, *Globodera pallida*, a potato cyst nematode (PCN), was first found in Idaho in 2006. Since its’ discovery, the focus has been to contain and eradicate this economically devastating pest of potato. *Globodera pallida* can cause up to 80% potato yield loss on susceptible potato varieties, readily spreads in infested soil, and survives for 30+ years. Eradication efforts have relied on soil fumigation. Since many nematicides have been banned, development of new methods for controlling this nematode are essential for success of the eradication program. One alternative control measure is the use of *Solanum sisymbriifolium*, commonly called litchi tomato or sticky nightshade which induces hatch but limits reproduction of PCN. However, because *S. sisymbriifolium* has little economic value as a crop and seeds are largely unavailable, it has not been widely adopted for use by producers in Idaho. Although poorly understood, there is evidence that this plant kills the nematode through production of toxins. Previous research indicates that pure solanaceous glycoalkaloids may be toxic to PCN with glycoalkaloids reducing hatch of PCN by up to 87%, reduce infection by 94%, and reduce reproduction by 99%. Currently, our research indicates that glycoalkaloids are found in higher concentrations in the leaf tissue rather than stems or roots. We are now identifying and purifying these compounds by liquid-liquid extraction and fractionation of infected and uninfected *S. sisymbriifolium* plants. Once fractionated, their impacts on PCN hatch and reproduction will be evaluated. Potential discovery of novel chemistries that may be either volatile or nonvolatile for nematicide development would be a valuable achievement for Idaho producers, or anyone dealing with PCN infestations.

**Reaction of Usda Soybean Germplasm Accessions to *Heterodera Glycines* Hg Type 2.5.7**.

Schumacher, Lesley

USDA, Agricultural Research Service, Crop Genetics Research Unit, Jackson, TN 38301

## Abstract

Soybean cyst nematode (*Heterodera glycines*, SCN) remains a devastating nematode to worldwide soybean (*Glycine max*) production. A major concern in the breeding effort for SCN resistance is the trend of reduced effectiveness of the primary source of resistance (PI 88788) in commercial soybean cultivars. For this reason, efforts at the USDA-ARS Crop Genetics Research Unit in Jackson, TN are focused on screening germplasm for new sources of SCN resistance. The goal of this work was to phenotype soybean accessions available in the USDA soybean germplasm collection for reaction to soybean cyst nematode populations. Ninety-nine germplasm accessions of various maturity groups were obtained from the USDA soybean germplasm collection and screened for reaction to HG Type 2.5.7. Five seedlings were included for each of the 99 accessions, susceptible controls, and indicator lines. Each seedling within a genotype represented a single replication, and the test was completely randomized and repeated twice using 2,500 SCN eggs/pot. Thirty days after SCN inoculation, soybean roots were blasted with water to dislodge females which were enumerated using a stereoscope. A female index (FI) from the indicator was calculated for the number of SCN females developed on each line from each replication and used for germplasm evaluation. Ratings of susceptible (FI>60), moderately susceptible (FI=31-60), moderately resistant (FI=10-30), and susceptible (FI<10) were assigned to the accessions. None of the accessions were resistant, 5 accessions were rated as moderately resistant, 27 accessions were rated as moderately susceptible, and 67 accessions were rated as susceptible. Data from both tests were combined for ANOVA of FI and means separated using Tukey’s HSD (*P* < 0.05). Accession PI 606409 had the greatest FI (164) while PI 612745 had the least (16). Germplasm screening will continue with these same 99 accessions for their reaction to HG Types 0 and 1.2.5.7 in 2022. It is important to continue the search for new sources of SCN resistance, which may provide more durable resistance. This will ultimately provide growers with options to achieve desirable yields in SCN-infested production areas.

**Developing *Steinernema Hermaphroditum* as an Experimental Genetic System for the Study of Entomopathogenic Nematodes**

Schwartz, Hillel^1^, C.-H. Tan^1^, M. Cao^1^, J. Peraza^1^, K. Raymundo^1^, J. K. Heppert^2^, H. Goodrich-Blair^2^ and P. W. Sternberg^1^

^1^Division of Biology and Biological Engineering, California Institute of Technology, Pasadena, CA, 91125

^2^Department of Microbiology, University of Tennessee, Knoxville, Knoxville, TN, 37996

## Abstract

Since its adoption as an experimental system roughly fifty years ago, the free-living soil nematode *Caenorhabditis elegans* has proved a powerful tool for discovery. The tractability of *C. elegans* in the laboratory and in particular its reproduction as self-fertilizing hermaphrodites have caused this species to be well suited to experimental genetics and a wide variety of molecular studies. With the success of *C. elegans* as inspiration, researchers are looking into the biology of other nematodes distantly related to *C. elegans*. By exploring the biology of these distant relatives of *C. elegans* it will be possible to better understand their shared and divergent biological processes and to uncover conserved and derived molecular pathways. By investigating nematodes with diverse lifestyles and habitats we will be able to study biological questions to which existing models such as *C. elegans* are poorly suited. Such opportunities can be found in the entomopathogenic nematodes, which offer the potential to investigate the mechanisms underlying interactions between insect hosts and pathogens, and between nematode hosts and their bacterial symbionts. Infective juvenile entomopathogenic nematodes carry within their gut a population of bacteria, with which they are mutualistically symbiotic. Upon finding a suitable insect host, the nematode and the bacteria act together to kill the insect, to consume it, and to reproduce, before eventually sending forth a new generation of infective juveniles to repeat the process. We will report on our progress developing the recently rediscovered entomopathogenic nematode species *Steinernema hermaphroditum* as a highly promising system for experimental genetics. We have found that *S. hermaphroditum* consistently reproduces as self-fertilizing hermaphrodites, with rare males that allow crosses. We have described the XX/XO chromosomal sex determination of this species, and have developed a highly inbred line of *S. hermaphroditum* that retains its ability to kill and consume insects in partnership with pathogenic bacteria. Using this line as the wild type we have demonstrated that mutants of *S. hermaphroditum* can be efficiently generated, tested for linkage and for complementation, and cryopreserved. We are continuing to develop a complete set of molecular-genetic techniques for the manipulation of this species in the laboratory. We will report on our first attempts to perform genetic screens that target the unique biology of these entomopathogenic nematodes, including efforts to investigate their interactions with their bacterial symbionts.

**Reklemel™ Active (Fluazaindolizine, Salibro™) Key Insights From a Global Nematicide Development Project**

Shirley, Andrew, S. Tewari, T. Thoden, J. Temple, A. Agi and D. Dyer

Corteva Agriscience™, Indianapolis, IN

## Abstract

Plant-parasitic nematodes remain a constant threat to quality and yield in food and fiber production across the globe. Nematode management has traditionally relied on fumigants and other broad-spectrum products but there has been a gradual shift to more selective and environmentally friendly options. This has been the result of an asserted effort by industry to direct considerable time and resources for the discovery and development of chemistries with nematicidal activity, as well as beneficial soil and environmental characteristics. Reklemel™ active is a proprietary nematicide with a novel mode of action and demonstrable ability to control economically important plant-parasitic nematode species globally as well as an environmentally safe and sustainable option. The discovery, development, and characterization of this chemistry, by Corteva Agriscience, comprises over 10 years of extensive research at the global level encompassing thousands of laboratory studies, greenhouse, and field trials. This work has defined key attributes of this novel chemistry including, nematode activity, soil behavior, and soil health compatibility. A US-focused summary of the key insights gained from the development of Reklemel™ as well as label and product registration updates will be presented.

**Molecular and Morphological Analysis of *Meloidogyne Naasi* from Golf Course Turfgrass in Idaho**

Skantar, Andrea M., Z. A. Handoo, M. R. Kantor, and M. N. Hult

USDA-ARS Mycology and Nematology Genetic Diversity and Biology Laboratory, 103000 Baltimore Ave., Bldg. 010A BARC West, Rm. 113, Beltsville, MD 20705

## Abstract

The barley root-knot nematode, *Meloidogyne naasi*, is one of the most important nematodes of monocots, and one of the most prevalent species associated with grasses. Sampling of declining bentgrass putting greens from a golf course in Ada County, Idaho, yielded two six-inch core soil samples that were received for identification, and a high number of *Meloidogyne* sp. juveniles were recovered by sugar centrifugal flotation. No females were recovered from the samples. Morphological observations and measurements were consistent with a diagnosis of *Meloidogyne naasi*. Comparison of multiple DNA markers from the population, including the ribosomal intergenic spacer region (IGS-2), 28S rDNA, mitochondrial DNA intergenic region, mitochondrial cytochrome oxidase I (COI), and nuclear heat shock protein 90 (Hsp90) showed the greatest similarity to those from previously reported for *M. naasi*. Phylogenetic analyses by Bayesian inference showed that the Idaho population clustered with other populations of *M. naasi* and separated from other *Meloidogyne* species. Morphological and molecular data confirming this population as *M. naasi* are presented.

**Crispr-Mediated Reverse Genetic Approaches in *C. Elegans* and *P. Pacificus* and Potential Modifications for Plant Parasitic and Entomopathogenic Nematodes**

Sommer, Ralf J. and H. Witte

Max-Planck Institute for Biology Tübingen, Dept. for Integrative Evolutionary Biology, Max-Planck-Ring 9, 72076 Tübingen, Germany

## Abstract

The CRISPR technology has revolutionized reverse genetics and has a major impact on biomedical research. In *C. elegans*, various CRISPR-associated approaches have been established that have further increased the methodological toolkit of this organism. In the applied sciences, plant and animal parasitic nematodes and also entomopathogenic nematodes might profit substantially from the development of related tools. We study the free-living nematode *Pristionchus pacificus* and have intensive experience with transferring methodologies originally developed in *C. elegans* to *P. pacificus*. *P. pacificus* is a model system for integrative evolutionary biology with a sophisticated toolkit for functional analysis. It has a 4-day generation time (20° C) and is a self-fertilizing hermaphrodite similar to *C. elegans*. Recent studies in this species focus on the regulation of predatory vs bacterial feeing as a result of mouth-form developmental plasticity, the mechanisms of predation and a sophisticated self-recognition system. In this talk, we will provide a brief overview on CRISPR applications, but also DNA-mediated transformation in *C. elegans* and *P. pacificus*. From there, we will discuss successful troubleshooting and potential modifications of standard protocols that might make them amenable to parasitic nematodes and EPNs.

**Management of *Rotylenchulus Reniformis*, Reniform Nematode, in Cotton Using Crop and Variety Rotation**

Soto-Ramos, Casiani M.^1,2^, T. A. Wheeler^2^, K. Lege^3^ and C. Monclova-Santana^1,2^

^1^Texas Tech University, Department of Plant & Soil Science, Lubbock, TX 79409

^2^Texas A&M AgriLife Research and Extension Center, Lubbock, TX 79403

^3^Corteva Agriscience, Lubbock, TX 79403

## Abstract

*Gossypium hirsutum* (upland cotton) belongs to the Malvaceae family and is valuable due to its fiber production. Reniform nematode (RN) can reduce cotton yield up to 90% when highly susceptible varieties are planted, and RN populations are high. Cotton production in Texas, the largest producer of cotton in the USA, is challenged by RN. One common agronomic practice used to control RN is crop rotation with sorghum, corn, or weed-free fallow. The objective of this research was to evaluate the effect of rotation of cotton varieties (resistant and susceptible to RN), sorghum, and weed-free fallow to control RN populations in West Texas. Two trials were established to evaluate the effectiveness of each rotational treatment. The first trial was established at the AgriLife Research and Extension Center in Lubbock, Texas. Rotation treatments consisted of a resistant cotton variety (R= DP 2143NR B3XF), a susceptible variety (S= DP 2044 B3XF) and weed-free fallow (F) in combinations RR, SS, RS, FS, FF, FR for two years. The second trial was established in a commercial farm in Lynn County, Texas. The rotation treatments included a resistant variety (R= PHY 332 W3FE), susceptible (S= PHY 300 W3FE) and sorghum (G) in combinations RR, RS and RG for two years. Both experimental designs were randomized complete block design (RCBD) with four and three repetitions, respectively. For both trials, the results obtained for the year 2021 (second year of rotation) are presented here. In the first trial, a decrease of 65% in the RN population was observed between the RR treatment (two consecutive years with the resistant variety) compared to the SS. The treatment with the highest yield in 2021 included the resistant variety- RR (917 kg lint/ha) and FR (815 kg lint/ha). The SS treatment had the highest RN density (835 RN/100 cm^3^ soil) and the lowest yield (516 kg lint/ha) compared to all other treatments. On the other hand, in the second trial the lowest RN density was observed in RG treatment (95 RN/100 cm^3^ soil). This represents a significant decrease compared to RS treatment (1,769 RN/100 cm^3^ soil). No significant differences were observed between RR (1,266 RN/100 cm^3^ soil) and RS. The highest yield was associated with RR treatment (2,233 kg lint /ha) compared to the RS (1,510 kg lint /ha). Implementing crop or variety rotation for nematode control remains an effective practice. Although rotation with sorghum or weed-free fallow decreases RN populations, resistant varieties such as DP 2143NR B3XF represent an alternative to control reniform nematode while also represents an increase in crop yield, resulting in an economic advantage for producers.

**Plant-Parasitic Nematode Regulatory Programs Implemented by Florida, USA**

Stanley, Jason, J. Brito, S. Vau and L. Whilby

Division of Plant Industry, 1911 SW 34th St, Gainesville, FL 32608

## Abstract

The Florida Department of Agriculture and Consumer Services, Division of Plant Industry (FDACS-DPI) is the state organization that conducts all regulatory actions for plant pests and diseases. Prior to 1955, plant-parasitic nematodes species present in Florida were not regulated. Nematodes affecting citrus were the first to be regulated in Florida. Currently there are two major certification Programs: Citrus Nurseries Certification and Ornamental Plants Certification (Rule Chapter 5B-44.003, Florida Administrative Code). Additionally, the state of Florida prohibits the introduction of exotic nematodes that may pose a threat to both its agriculture and natural resources (Rule Chapter 5B-3.038, Florida Administrative Code). Many of the plant-parasitic nematodes occurring in Florida are prohibited by other states and countries. The nematology section is responsible for the certification of plant-parasitic nematode-free Ornamental and Citrus nursery products, inter and intra-state as well as by other countries. A major benefit of the Ornamental Nursery Certification Program is enabling ornamental growers to ship plants to both national and international markets, supporting Florida’s multi-billion-dollar agricultural industry. The nematology section collects, maintains, and updates all relevant information on plant pest regulatory nematodes for states’ and countries’ regulation. Regulatory activities, permits, and agreements facilitating shipment of plant material to those states and countries which impose quarantine restrictions will be discussed.

**Group Movement and Joining Behavior in Steinernematid Nematodes**

Stevens, Glen^1^, F. Kaplan^2^, D. Shapiro-Ilan^3^, H. Alborn^4^, P. Schliekelman^5^ and E. Lewis^1^

^1^University of Idaho, Department of Entomology, Plant Pathology and Nematology, Moscow, ID 83844

^2^Pheronym, Inc. Woodland, CA 95695

^3^USDA-ARS, Byron, GA 31008 ^4^USDA-ARS, Gainesville, FL 32608

^5^University of Georgia, Department of Statistics, Athens, GA 30609

## Abstract

Entomopathogenic nematodes (EPN) are obligate parasites of insects and are often used for biological control. We have both fundamental and applied interest in understanding social behaviors in these worms and have conducted a series of experiments to assess group movement of EPN in the genus Steinernema. Through a combination of sand arena and olfactometer assays we have observed differences in dispersal in single-vs. mixed-species environments, and context-dependent group joining behavior. These behaviors vary significantly across the three species assayed (*Steinernema carpocapsae*, *S. feltiae* and *S. glaseri*) and can be significantly altered by factors associated with contact with the insect host. Even in the absence of insect hosts, host cues, or environmental cues (or when these cues were uniform in the experimental arena), EPN dispersed in a non-random manner. The combination of these assays illustrates the complexities of individual and group movement in these important species, and provides insight into nematode distributions, spatial and temporal population structures, and community dynamics.

**Molecular Diagnostics of *Meloidogyne Enterolobii* and Phytosanitary Implications of this Pest in California**

Subbotin, Sergei A. and J. Burbridge

Plant Pest Diagnostic Center, California Department of Food and Agriculture, 3294 Meadowview Road, Sacramento, CA 95832

## Abstract

*Meloidogyne enterolobii* is considered one of the most important root-knot nematode species because of its ability to overcome resistance in important crops carrying genes of resistance to the main *Meloidogyne* spp. Presently, *M. enterolobii* is under the highest A rating in California as a pest that has not been known to occur or is under official control in the State of California. There are several records of the detection of *M. enterolobii* in incoming shipments of plants from Florida, Texas, Puerto Rico to California. Rapid diagnosis tools for detection of root-knot nematodes play an important role in disease control and eradication programs. Recombinase polymerase amplification (RPA) assays have been developed that target the different genes of several root-knot nematode species: *Meloidogyne arenaria*, *M. hapla*, *M. incognita*, *M. javanica* and *M. enterolobii*. RPA assays using TwistAmp® Basic, TwistAmp® exo kits and TwistAmp® nfo kits (TwistDx, UK) allow for the detection of nematode species directly from plant tissues and crude nematode extracts of all life stages without a DNA extraction step. The study included three steps: i) testing and selection of RPA specific primer combinations; ii) validation of sensitivity and specificity for RPA assays using real-time fluorescence detection (real-time RPA) and lateral flow dipsticks (LF-RPA); and iii) practical evaluation of RPA assays with field samples. The results of the -RPA assays with a series of crude nematode extracts show reliable detection of 1/10 of a second-stage juvenile (J2) of root-knot nematodes and 1/100 of a female in reaction tubes within 15 min for real-time RPA and 30 min for LF-RPA. RPA assays provide affordable, simple, fast, and sensitive detection of nematodes. Application of the LF-RPA assay has great potential for application and implementation of nematode diagnostics in the lab, field or in areas with a minimal laboratory infrastructure.

**Diversity, Distrubution and Biocontrol Potential of Entomopathogenic and Entomophilic Nematodes from Selected Agricultural areas of Bukidnon Province, Philippines**

Sumaya, Nanette Hope^1,2^, M. Suan^1,2^, J. K. Lluisma^1,2^, M. F. Maureal^1,2^ and P. Nimkingrat^3^

^1^Department of Biological Sciences, College of Science and Mathematics, Mindanao State University-Iligan Institute of Technology, A. Bonifacio Ave., Tibanga, Iligan City 9200, Philippines

^2^FBL-Nematology Research Group, Premier Research Institute of Science and Mathematics, Mindanao State University-Iligan Institute of Technology, A. Bonifacio Ave., Tibanga, Iligan City 9200, Philippines

^3^Department of Entomology and Plant Pathology, Faculty of Agriculture, Khon Kaen University, Mittraphap Rd., Nai-Muang, Muang District, Khon Kaen, 40002, Thailand

## Abstract

The increasing need to reduce chemical pesticide use has driven the interest in exploiting entomopathogenic and entomophilic nematodes (EPNs) as biological control agents (BCA) against several economically important agricultural pests. This study aimed at investigating the diversity, distribution and biocontrol potential of locally isolated nematodes from selected agricultural areas of Bukidnon province, Philippines. From randomly 55 selected farms as sampling sites in Bukidnon, 89 soil samples (32.84%) harbored nematodes out of 271 collected soil samples. Through morphology and analyses of the D2-D3 expansion segment of the 28S rRNA, 18S rRNA, ITS and mitochondrial COI regions, we have identified a total of 8 EPN species from 4 genera. For genus *Oscheius*, we have *O. carolinensis* (24 isolates), *Oscheius* spp. (5 isolates), *O. myriophila* (4 isolates), *O. colombiana* (2 isolates) and 2 isolates were identified to be *Heterorhabditis* sp. For *Metarhabditis* and *Rhabditis*, we have *M. blumi* (5 isolates), *M. rainai* (6 isolates) and *Rhabditis* spp. (2 isolates). Moreover, the biocontrol potential of these 8 nematode species was investigated against superworm, *Zophobas morio* and 6 EPNs for cotton cutworm, *Spodoptera litura* under laboratory conditions. Last instar larvae of *Z.morio* were exposed to 200IJs/ml whereas 4 developmental stages of *S. litura* (L1/L2, L4/L5), 2 and 5 days old pupae were exposed to 100, 300 and 500 IJs/ml. All tested isolates were found to be pathogenic against larvae of *Z. morio* and in all developmental stages of *S. litura*, particularly, *O. colombiana* which induced a 100% mortality rate for both L1/L2 and L4/L5 stages in the 4^th^ day post nematode inoculation. For comparison, we used the commercially available *Steinernema saimkayai* from Thailand which recorded a 100% mortality after 4 and 5 days post nematode inoculation for both L1-L2 and L4-L5, respectively. After 8 days, *O. colombiana* recorded more than 70% and 96% mortality rate in both the 2 and 5 days old pupae whereas *S. saimkayai* had more than 72% and 97% mortality rate, respectively. Therefore, locally isolated EPN species revealed an insecticidal potential against *Z.morio* and *S. litura*. Screenhouse and field trials are currently being conducted to further investigate the efficacy of the nematode isolates.

**Postembryonic Proliferation of Intestine Cells in the Entomopathogenic Nematode *Steinernema Hermaphroditum***

Tan, Chieh-Hsiang and P. W. Sternberg

Division of Biology and Biological Engineering, California Institute of Technology, Pasadena, CA 91125

## Abstract

The development of multicellular organisms is a complex process that is repeated with high precision in each generation. An especially striking example of precise programmed development can be found in nematodes, some of which have invariant somatic cell lineages. In the free-living soil nematode *Caenorhabditis elegans*, about 1090 somatic nuclei are generated, and their fate is well understood. The invariant cell lineages proved crucial in identifying and characterizing signaling pathways, including those of cell death and microRNAs. However, it is understood that not all nematodes have fixed cell lineages, and we think that given the extreme range in body sizes of different nematode species, supporting the growth of larger species may require additional or even continuous cell proliferation. Taking advantage of the newly established genetically tractable clade IV entomopathogenic nematode *Steinernema hermaphroditum*, which is significantly larger than, and evolutionarily distant from, *C. elegans*, we are conducting comparative analyses of developmental processes. We chose to focus first on postembryonic intestinal development. The nematode intestine is the primary organ for nutrient uptake and makes up a significant portion of the volume of the worm. In the clade V nematode *C. elegans*, just 20 cells constitute the entire organ, accounting for about 1/3 of the worm volume. In *C. elegans*, all 20 intestinal cells are formed during embryonic development. Some of these cells undergo one round of nuclear (but not cellular) division at the first molt, but none undergo cell division postembryonically. This small and fixed number of cells stands in contrast to the exponential size increase seen during the animal’s postembryonic development. A similar observation has also been made in the clade IV nematode *Panagrellus redivivus*, in which all 18 intestinal cells of the fully formed adult are present at hatching. It was, therefore, surprising when we found that in *S. hermaphroditum*, most, if not all, of the >100 intestinal cells in a mature adult are generated by postembryonic cell proliferation. Animals were observed at hatching to possess 28 intestinal cells. These duplicated around the time of the first molt to about 56, and subsequently increased in number at each larval stage. Preliminary evidence suggests that this increase in the intestinal cell number of *S. hermaphroditum* may depend on nutrient conditions. We think this work could provide insight into how nematodes that are larger in size expand cell lineages to fulfill their needs.

**Using Lidar (Agerpoint™) to Characterize Novel Nematicide Reklemel™ Active (Fluazaindolizine, Salibro™) for the Management of Key Plant-Parasitic Nematodes in tree Nuts**

Tewari, Sunil^1^, S. Colbert^1^, K. Steddom^2^ and T. Thoden^3^

^1^Corteva Agriscience, 9330 Zionsville Rd., Indianapolis, IN 46268

^2^5 Laboratory Drive, Suite 3250, Research Triangle Park, NC 27709

^3^Corteva Agriscience, 81677 Munich, Germany

## Abstract

LiDAR (Light Detection and Ranging) is a novel tool that can be leveraged to capture precise agricultural data on multiple growth parameters of tree crops such as plant height, canopy density, canopy diameter, canopy volume, and trunk diameter. These data can be analyzed to deliver precise analytics and actionable insights. Agerpoint is a pioneer organization that has developed a spatial data platform for agriculture and climate insights powered by scalable terrestrial data capture and precise automated plant-level analytics, specifically leveraging the power of LiDAR. Reklemel™ active (fluazaindolizine, Salibro™) is a novel, non-fumigant, chemical nematicide discovered and being developed by Corteva Agriscience for the control of key plant-parasitic nematodes infesting a wide range of annual and perennial crop groups in North America including tree nuts. It has demonstrated selective and effective control of key nematode pests including a wide range of root-knot species (*Meloidogyne* spp.) as well as some root-lesion nematodes (*Pratylenchus* spp.), among others. Plant-parasitic nematodes pose serious challenge to tree nut growers, especially during early establishment as tree growth is inhibited. Results from comparative trials where LIDAR was used to characterize the efficacy of Reklemel™ against key plant-parasitic nematode species in tree nuts will be presented.

**Evaluating Entomopathogenic Nematodes as a Control for Spotted Wing Drosophila**

Thapa, Rambika, E. Cole, J. Perkins, R. Isaacs and M. Quintanilla

Michigan State University, Dept. of Entomology, East Lansing, MI 48823

## Abstract

Spotted wing drosophila (SWD) is a globally invasive pest whose preference for soft-skinned fruits has caused significant yield loss to small fruit cropping systems including blueberries and raspberries. Female SWD have a serrated ovipositor allowing it to lay eggs on unripe berries making the fruits unmarketable. SWD has an enormous reproduction rate and short generation time of only 2-3 weeks which makes management difficult. To find alternatives for repeated pesticide application and decrease insect resistance to pesticides, we completed a study investigating the potential for entomopathogenic nematodes (EPNs) to control SWD. The objectives of this study were to 1) evaluate three EPN species (*Heterorabditis bacteriophora, Steinernema carpocapsae, and Steinernema feltiae*) in their ability to reduce SWD survivorship when applied during 3rd instar, pupal, and infested fruit life stages under laboratory conditions and 2) determine EPN efficacy in reducing SWD populations under field conditions in blueberries. Laboratory assays were completed by creating arenas in round test vials filled with 11g of sand. For each EPN species, 10 arenas were created that included either 5, 3rd instar SWD larvae, 5 SWD pupae, or 1-2 SWD infested berries with an average of 10 eggs per arena. Within each arena, approximately 250 individuals of the appropriate EPN species were inoculated. Distilled water served as a control. After 15 days, the number of emerged adults was counted. Last year's laboratory assay showed that *Steinernema feltiae* and *Heterorhabditis bacteriophora* reduced adult emergence compared to *Steinernema carpocapsae*. Based on these results, *S. feltiae* and *H. bacteriophora* were selected for further testing against an untreated control in the field. Plots consisted of 7 'Bluecrop' bushes and were organized in a CRBD. EPNs were applied every two weeks starting in July at labeled rates and over the course of the fruiting season, the SWD population was monitored via traps and salt testing of collected fruits. The results are forthcoming so, for this year we are repeating the same field trial as well as we are planning a new field trial where we are evaluating the effect of single versus multiple EPNS applications. Though EPNs may not be able to provide 100% control of SWD populations, they may be a valuable addition to the IPM toolbox, however, another season of fieldwork is needed to determine the impact of EPNs on SWD population dynamics.

**Surveys on Plant Parasitic Nematode Distribution in Michigan Corn and Vegetable**

Thapa, Sita^1^, E. Darling^1^, E. Cole^2^, K. Poley^3^ and M. Quintanilla-Tornel^1^

^1^Michigan State University, Department of Entomology, East Lansing, MI 48824

^2^Agronomist, J.R. Simplot Company, Boise, ID 83706

^3^Research Manager, Corn Marketing Program of Michigan, Lansing MI 48906

## Abstract

Michigan has the second most diverse agriculture industry in the United States after California, producing more than 300 commodities. Plant-parasitic nematodes (PPNs) cause economical yield loss on many commodities. Knowing nematode species, abundance, and distribution in each crop is key to recommending management strategies and reducing yield loss due to its damage. We carried out two regional-scale soil surveys, the first one in 2017 in vegetable fields and the second in 2018 in corn fields in Michigan. In both surveys, more than 300 soil samples were collected and analyzed. Nine different PPNs genera were identified in Michigan vegetables: *Pratylenchus* (lesion), *Meloidogyne* (root-knot), *Heterodera* (cyst), *Tylenchorohynchus* (stunt), *Helicotylenchus* (spiral), *Paratylenchus* (pin), *Criconema* (ring), *Xiphinema* (dagger), and *Trichodorus* (stubby root). The average number of PPNs was lowest in the initial samples, increased in mid-season, and then declined again in the harvest samples. The highest number of PPNs were found in muck soil (258 ± 84.2), followed by sand (50.7 ± 9.4) and sandy loam (49.2 ± 12.7), and the least number of PPNs were found in clay soil (1 ± 0.5). Lesion nematodes were significantly higher in root vegetables (carrot, parsnip, potato, and turnip), compared to leafy greens (cabbage, lettuce, and parsley) (P=0.078) and fruit-bearing vegetables (cucumber, pepper, tomato, and zucchini) (P = 0.0043). Pin nematodes were highest in root vegetables (P=0.0003). Similarly, in a corn survey, ten different major genera of PPNs were identified in Michigan corn fields: *Longidorus* (needle), *Helicotylenchus* (spiral), *Paratylenchus* (root-lesion), *Meloidogyne* (root-knot), *Heterodera* (cyst), *Hoplolaimus* (lance), *Tylenchorhynchus* or *Merlinius* (stunt), *Paratylenchus* (pin), *Criconemella* (ring), and *Xiphinema* (dagger). Lesion nematodes were most prevalent in muck soil, while stunt nematode prevalence was significantly affected by the soil type. Needle nematodes were least abundant in irrigated soils, and, by contrast, stunt nematodes were higher in non-irrigated soils. Spiral nematodes were the most common PPNs in Michigan corn in all cropping systems. Our results also indicated that the population of most of the PPNs in Michigan vegetables in 2017 and corn in 2018 was lower than the thresholds. These findings will be helpful to plan future nematode studies in Michigan and in further developing and evaluating corn and vegetable nematode management strategies, if necessary.

**Effect of Biochar and Compost Top-Dress Applications on *Belonolaimus Longicaudatus* Populations in Berumudagrass**

Thomason, Malone^1^, C. Khanal^2^, J. Kerns^3^ and J. Roberts^1^

^1^Pee Dee Research and Education Center, Florence, SC 29506

^2^210 Biosystems Research Complex, Clemson, SC 29634

^3^Varsity Research Building Room 1108, Raleigh, NC 27606

## Abstract

Sting nematode (*Belonolaimus longicaudatus*) can impact a wide range of hosts and is particularly devastating to turfgrass areas constructed on high sand content rootzones. Biochar and compost have arisen as potential control options for nematodes in different cropping systems. Previous research has shown varying results with indications that the type of biochar or compost plays a significant role. Research on biochar and composts for turfgrass nematodes is needed, as turfgrass managers are constantly searching for new methods to enhance turf quality. A 2-year study was initiated in 2020 to examine how biochar and compost topdressings impact bermudagrass maintained as a golf course fairway. Five treatments (i.e., non-treated control, 50/50 biochar/compost blend applied at 489.3 kg ha^−1^, 50/50 biochar/compost blend applied at 978.5 kg ha^−1^, biochar alone applied at 489.3 kg ha^−1^, and biochar alone applied at 244.6 kg ha^−1^) were arranged in a randomized complete block design with 6 replications. Treatments were applied evenly to the surface of 1.8 m x 3 m plots of turfgrass twice in the spring and fall of each year. Plots were assessed regularly for turfgrass visual quality (i.e. 1-9 scale; 9=best), turfgrass color (i.e., NDVI), and monthly soil samples for monitoring nematode populations. Visual quality and NDVI measurements were used to calculate area under progress curve values using the trapezoidal method. All data was subject to analysis of variance using the mixed procedure in SAS 9.4. Treatment means were separated using Tukey’s honest significant difference at 0.05 probability. A moderate but significant correlation was found between turf quality and sting counts (Pearson coefficient of r = -0.2 *p* < .0001). The high rate of the biochar/compost blend and the biochar significantly increased turf quality when compared with the non-treated control (*p* < .001). Differences in sting nematode populations were not observed in response to any topdressing treatment, but results show that topdressings can improve turfgrass quality even when high sting populations exist. Future research to incorporate a larger amount of material into the root zone may prove beneficial in reducing sting nematode populations.

**Characterization of a Lima Bean Diversity Panel to Infection by Southern Root-Knot Nematode**

Traverso, Eboni, E. Ernest and A. M. Koehler

University of Delaware, Carvel and Research Education Center, 16483 County Seat Hwy, Georgetown, DE 19947

## Abstract

Lima beans (*Phaseolus lunatus*) have a rich history as one of Delaware’s cornerstone crops in the vegetable processing industry. Due to sandy soils in the region, southern root-knot nematode (RKN, *Meloidogyne incognita*), is a pathogen that accumulates in the soil and causes devastating yield losses and economic injury. RKN feeding can cause extreme galling of the lima bean root system, resulting in disrupted water and nutrient uptake, decreased plant vigor, and reduced lima bean yield potential. There has not been a recent survey of RKN resistance in current commercially available lima bean cultivars or diverse germplasm that could possess resistance useful for breeding. It is necessary to establish which display resistance, if any, for use in resistance breeding programs and to recommend to producers experiencing RKN disease pressure in their fields. 256 lima bean inbred lines from the USDA collection, the UD Lima Bean Breeding Program, various commercial seed distributors, and individual accessions from around the world were selected to evaluate RKN resistance. This trial was established using conetainers in a greenhouse with a sandy soil medium. Individual seedling-stage plants were inoculated with 500 freshly extracted RKN second-stage juveniles. Plants were grown for 6-8 weeks, then root systems were evaluated for root galling by visual examination, and for reproduction by counting the number of egg masses in the root tissues after staining with erioglaucine. Genotypes were separated into seven groups based on gall rating. Three percent of genotypes grouped in the lowest gall rating. Genotypes were separated into five groups based on egg counts, with seventy-one percent in the lowest egg count group. RKN resistance is evaluated on the basis of gall rating and reproduction, and while they may be independent of one another, ideal genotypes to use as parents in the breeding program have a reduction in both. Fifteen genotypes were identified with lowest gall and egg count ratings to be advanced for further screening and possible incorporation into the UD Lima Bean Breeding Program.

**Conspecific and Heterospecific Dispersal Effect of Exometobolomes on Some Entomopathogenic Nematode Species**

Ulu, Tufan Can^1^, H. Erdogan^2^, K. R. Cruzado^3^, B. Sadic^3^ and E. E. Lewis^3^

^1^Bilecik Şeyh Edebali University, Faculty of Agriculture and Natural Sciences, Department of Plant Protection, Bilecik, Turkey

^2^Faculty of Agriculture, Department of Biosystem Engineering, Bursa Uludag University, Bursa, 16059, Turkey

^3^University of Idaho, Department of Entomology, Plant Pathology and Nematology, Moscow, ID 83844

## Abstract

Ascarosides are known as nematode pheromones and have a significant effect on the behavior and development of nematodes. In addition, ascarosides elicits plant defenses including salicylic acid and jasmonic acid-mediated responses. Entomopathogenic nematodes communicate among individuals using ascarosides and secrete ascarosides and other compounds into the environment. In this study, we investigated the effects of entomopathogenic nematode exometabolome (which contains ascarosides and other compounds) on conspecific and heterospecific interactions of four entomopathogenic nematode species from two genera (*Steinernema carpocapsae*, *S. feltiae*, *S. diaprepesi* and *Heterorhabditis bacteriophora*). Nematode suspensions were prepared in flasks at a ratio of 1:5 (distilled water / infective juvenile) to obtain exometabolome. The flasks were maintained at 15 °C for 1,7, 10 or 14 days and thus different concentrations of exometabolome were collected on different days. Trials were performed in two-arm olfactometers with exometabolome solution in the center and distilled water in the arms, and IJs were injected to the center. Mostly, there was no conspecific dispersal effect, except for *S. carpocapsae*. In *S. carpocapsae*, higher dispersal effect was found as the incubation time of nematode suspension increased. When the heterospecific effect was examined, a dispersal effect was observed in 14-day exometabolome, while variability was observed in the other days. In the heterospecific experiments, it was determined that exposure to exometabolome increased the dispersal of infective juveniles in general.

**Florida Department 0f Agriculture and Consumer Services-Division 0f Plant Industry Laboratory Identification Sample Tracking Application - List**

Vau, Silvia, L. Whilbe, J. Stanley and J. Brito

Division of Plant Industry, 1911 SW 34th St, Gainesville, FL 32608

## Abstract

The Florida Department of Agriculture and Consumer Services, Division of Plant Industry (FDACS-DPI) Nematology Section is tasked with certifying Florida ornamental and citrus nurseries to be free of plant-parasitic nematodes regulated by other states and countries. This process guarantees that these nurseries are in compliance with the regulations applied upon them, greatly facilitating commerce and supporting Florida’s multibillion dollar agricultural industry. The FDACS-DPI Plant Inspection Bureau includes field trained nematode inspectors that collect samples consisting of soil and roots, as well as other plant parts from all over the state. The Nematology Section has technicians that process samples, and nematologists that perform nematode identifications. For the efficacy of this chain, an application (“LIST”) was created to capture sample information collected in the field through the entire process leading to the final nematode report. This new application maintains the integrity of the information without data losses that would negatively impact regulatory decisions and applied research. Each nematology sample in LIST is composed of five main sections: (i) sample submission, (ii) sample processing, (iii) sample reporting, (iv) sample search, and (v) taxonomy encyclopedia; connected to a unique number and link generated during sample submission, an ecofriendly alternative to the original paper format. The main characteristics of LIST that are essential for the FDACS-DPI Nematology Section include addition of: photos; GPS, TRS, USNG coordinates; internal codes provided by FDACS-DPI to citrus and ornamental nurseries during their registration and certification process (firm number); detailed comments about sampling conditions and techniques, environmental conditions, and host status; notifications and comments for all participants; and various types of search mechanisms and taxonomic elements. With LIST, FDACS-DPI ensures that the most modern technologies like GIS applications, ESRI are linked for efficiency in the field and in the laboratories.

**Advancing our Understanding of the Beech Leaf Disease Nematode *Litylenchus Crenatae Mccannii***

Vieira, Paulo^1^, M. Kantor^1^, J. Mowery^2^, J. Shao^3^, Z. A. Handoo^1^, L. K. Carta^1^ and J. D. Eisenback^4^

^1^USDA-ARS Mycology & Nematology Genetic Diversity & Biology Laboratory, Beltsville, MD

^2^USDA-ARS Electron and Confocal Microscopy Unit, Beltsville, MD

^3^USDA-ARS Molecular Plant Pathology Laboratory, Beltsville, MD

^4^School of Plant and Environmental Sciences, Virginia Tech, Blacksburg, VA

## Abstract

The North American beech leaf disease nematode, *Litylenchus crenatae mccannii*, is recognized as a newly emergent nematode species that causes beech leaf disease (BLD) in beech trees (*Fagus* spp.) in North America. Since the first report of BLD on *Fagus grandifolia* in Ohio in 2012, the disease has rapidly spread to other states and Canada. This nematode has been so far reported in Pennsylvania, New York, Connecticut, Massachusetts, Maine, Rhode Island, New Jersey, West Virginia and Virginia, as well as Ontario (Canada). Leaf symptoms include swelling and darkening of interveinal tissues as well as chlorosis, while tissue necrosis and leaf curling occur at later stages of the disease. As a result, the mortality of nematode infected understory beech trees has been reported after several years of infection in the United States. The fast dissemination of this nematode can impose a dramatic effect on beech forest ecosystems and natural diversity in North America. Little information on the molecular and cellular interaction between this nematode and its hosts is available. To advance our understanding into the host/ nematode interaction, we investigated the cytological aspects of this plant-nematode interaction using bright-field and transmission electron microscopy. To obtain insight into the transcriptome of this nematode, we used Illumina mRNA sequencing analysis of a mixed stage population originally collected from Virginia. Over 140 million reads were obtained, and a *de novo* transcriptome assembly resulted in ~34,000 transcripts for the first time for this nematode. Using different gene comparison analyses, a list of candidate effector genes was identified. We confirmed spatial expression of transcripts within the esophageal glands of *L. crenatae mccannii* by *in situ* hybridization, revealing novel pioneer effectors to this species and across the Nematoda phylum. These analyses provide additional data for understanding the mode of parasitism of this new emergent plant-parasitic nematode.

**Quantitative Resistance in *Citrullus Amarus* Reduces *Meloidogyne Enterolobii* Galling and Reproduction**

Waldo, Benjamin^1^, S. Branham^2^, A. Levi^3^, P. Wechter^3^ and W. Rutter^3^

^1^USDA-ARS, Beltsville, MD 20705

^2^Clemson University, Coastal Research and Education Center, Charleston, SC 294144

^3^USDA-ARS, Charleston, SC 29414

## Abstract

*Meloidogyne enterolobii* is a virulent species of root-knot nematode that threatens watermelon (*Citrullus lanatus*) production in the southeast United States. There are no known sources of root-knot nematode resistance in cultivated *C.s lanatus* against. Genotypes of a wild watermelon relative, *Citrullus amarus*, have variable levels of resistance against *M. incognita*. We examined 109 inbred *C. amarus* lines for resistance against *M. enterolobii* in greenhouse experiments. Phenotypic trait ranges of *C. amarus* inbred lines were broad, but consistent. The mean percent root system galled ranged 10 - 73%, mean egg mass counts ranged 0.3 - 64.5, and mean eggs per gram of root ranged 326 - 146,160. We used each of these resistance phenotypes to conduct a genome wide association and found significant marker-phenotype associations that we used to identify candidate genes involved with *M. enterolobii* resistance. Eleven single nucleotide polymorphisms (SNPs) associated with resistance were distributed across chromosomes Ca03, Ca04, and Ca08. SNPs associated with reduced galling and egg masses were located on Ca03 and SNPs associated with reduced eggs per gram of root were located on Ca04 and Ca08. SNPs associated with Candidate host resistance genes located within 100kb of significant SNPs encode for calmodulin binding proteins, histone-lysine N-methyltransferase, and rRNA N-glycosidase. The results of this study suggest multiple genes are involved with *M. enterolobii* resistance in *C. amarus* and the SNPs identified may be informative for future *M. enterolobii* resistance breeding in watermelon.

**Screening for Reniform Nematode Resistance in Commercially Available Soybean Varieties Planted in Louisiana**

Watson, Tristan T.^1^ and P. Price^2^

^1^Department of Plant Pathology and Crop Physiology, LSU AgCenter, Baton Rouge, LA 70803

^2^Macon Ridge Research Station, LSU AgCenter, Winnsboro, LA 71295

## Abstract

The reniform nematode (*Rotylenchulus reniformis*) is an economically important plant-parasitic nematode on several crops grown in Louisiana, including soybean. Feeding by this semi-endoparasitic nematode results in poor root system development and stunted aboveground growth, which ultimately results in reduced yields. Soybean varieties with resistance to other damaging nematode species, including southern root-knot nematode (*Meloidogyne incognita*) and soybean cyst nematode (*Heterodera glycines*), are readily available; however, susceptibility to reniform nematode in commercially available soybean varieties is often unknown. Using a series of greenhouse and field trials, the aim of this study was screen soybean varieties for their susceptibility to the reniform nematode. In 2021, twenty of the top selling soybean varieties in Louisiana were planted into two reniform nematode-infested fields (Winnsboro and St. Joseph, Louisiana). A complementary greenhouse variety screening experiment was conducted using the same twenty soybean varieties planted into pasteurized potted field soil inoculated with each reniform nematode population. All soybean varieties examined supported reproduction of both reniform nematode populations, indicating no resistance to the reniform nematode in commonly planted soybean varieties in Louisiana. Despite all soybean varieties being susceptible to reniform nematode feeding, differences in mid-season nematode soil population densities, root parasitism, and yield were observed. Progeny P4444RXS had low reniform nematode soil and root population densities in both field locations relative to many of the other soybean varieties examined during the mid-season sampling date. Similarly, Armor 48-D25 supported low reproduction of both populations of reniform nematode in the greenhouse experiments and had low reniform nematode soil population densities in both field locations during mid-season sampling. In 2022, six of the least susceptible commercial soybean varieties from our 2021 screening trials were planted alongside six resistant soybean varieties from the University of Missouri breeding program into both field locations in Winnsboro and St. Joseph. Preliminary results from the 2022 field trials will be discussed.

**Field Evaluation of Easter Lilies Transformed with a Rice Cystatin Gene for Root Lesion Nematode**

Westerdahl, Becky^1^, L. Riddle^2^, D. Giraud^3^ and K. Kamo^4^

^1^Dept. of Entomology and Nematology, University of California (UC), Davis, CA 95616

^2^Easter Lily Research Foundation, Brookings, OR 97415

^3^UC Cooperative Extension, Eureka, CA 95503

^4^Floral & Nursery Plants Research Unit, USDA, Beltsville, MD 20705

## Abstract

Easter lilies, *Lilium longiflorum* cv. Nellie White are a staple of the floral industry. In the U.S. most of the Easter lilies are grown in Oregon and California along the coast where there is a micro climate that is favorable to growth of lilies. The main pest when growing lilies in the field is *Pratylenchus penetrans*, the root lesion nematode. Easter lilies are one of the most expensive crops to produce because of the cost of chemicals used to control *P. penetrans* and other pathogens that infect the lilies. A previous study had shown that transgenic Easter lilies containing a rice cystatin gene (Oc-IΔD86 that has a deleted Asp86) were resistant to *P. penetrans in vitro*. This study examined growth characteristics of four independently transformed lines of the cystatin Easter lilies for three seasons in the field in Brookings, Oregon, compared to a non-transformed control. Lilies were planted into four different soil treatments: 1) Untreated, 2) Untreated plus organophosphate, 3) Fumigant, and 4) Fumigant plus organophosphate. Each season, the foliage, basal and stem roots were visually evaluated for the transgenic and non-transformed Easter lilies. After harvesting the plants, the bulbs and bulblets were either weighed or their circumference measured. Nematodes were counted following their extraction from the roots. The transgenic Easter lilies were not entirely resistant to lesion nematode, but significant reductions in nematode levels compared to the non-transformed lilies were evident.

**Using Anaerobic Soil Disinfestation as Preplant Soil Treatment for Perennial Crop Nurseries**

Westphal, Andreas^1^, Z. T. Z. Maung^1^, T. R. Buzo^1^, D. A Kluepfel^2^, and M. Stanghellini^3^

^1^Department of Nematology, University of California Riverside, Parlier, CA

^2^USDA-ARS, Crops Pathology and Genetics Research Unit Davis, CA

^3^TriCal Inc., Hollister, CA

## Abstract

In an open-field nursery, bareroot plants of almond, walnut, and grape are tested for the presence of plant-parasitic nematodes at harvest unless the field has been certified nematode-free before planting. Treatment protocols to permit certification include the use of methyl bromide, 1,3-dichloropropene, chloropicrin and metam sodium. *Meloidogyne* spp. and *Pratylenchus vulnus* are the most notorious nursery nematode pests because of the risk for spread. Preplant soil treatment in perennial crop nurseries needs to be effective to a 5-ft depth to avert the risk of root infections of up to 27 months (walnut). In a nursery field infested with *P. vulnus*, the following treatments were applied: (i) non-treated; (ii) Telone II at 340 L/ha; (iii) methyl bromide at 450 kg/ha; and (iv) anaerobic soil disinfestation (ASD) at 20 ton/ ha rice bran. Telone and methyl bromide were applied with tractor-mounted shanks. In ASD, the rice bran was spread on the soil surface and incorporated to 10-cm depth using a rototiller. Drip irrigation lines were placed on top of the soil, and plots were covered with a 25-μm thick, clear tarp. The control and ASD received an initial water drench equivalent to 150 L/m2 via drip irrigation that was followed by intermittent watering in ASD. One experiment was initiated at the beginning and a second one at the end of August. In early fall, all plots were seeded to one row of peach rootstock ‘Nemaguard’ and one row of walnut rootstock ‘Paradox’. In June of the second vegetation year, plots were split, and one subplot received a postplant soil drench application of Salibro. At planting of the rootstock seeds, treatments ii, iii and iv had reduced the numbers of *P. vulnus* close to the detection level throughout 1.5 m soil depth. At stock harvest of the peach rootstock (one year after planting), ASD plots had significantly more plants than the control. Negligible numbers of *P. vulnus* were detected in peach roots. At walnut removal (two years after planting), in subplots that had received the Salibro postplant application, *P. vulnus* levels in the walnut roots were close to the detection limit in ii, iii and iv, significantly different from the control. The data suggested the potential merit of ASD as a soil preplant treatment for the production of nematode-free nursery stock.

**Efficacy of a Novel, Dual Mode of Action Nematicidal Seed Treatment**

Whalen, Adam, J. Huggins and J. Mullock

BASF, 2 TW Alexander Drive, Research Triangle Park, NC 27709

## Abstract

Soybean cyst nematode, *Heterodera glycines*, is the most damaging soybean pest in the United States. BASF has developed a new seed treatment nematicide containing the active ingredients, fluopyram and *Bacillus firmus* I-1582. Data will be shared demonstrating how the new seed treatment performs in various pest environments.

**Emergence and Chemotaxis of Unmated Soybean Cyst Nematode Males 0f Specific Ages**

Wlezien, Elizabeth and G. L. Tylka

Department of Plant Pathology, Entomology, and Microbiology, Iowa State University, Ames, IA 50011

## Abstract

Mating of males and females is required for sexual reproduction of the soybean cyst nematode (SCN), *Heterodera glycines*. Adult SCN males emerge from soybean roots and enter the soil to mate with mature SCN females that have ruptured through and are partially exposed on the root surface. Vanillic acid was reported to be a sex pheromone produced by SCN females that attracts males and elicits mating behavior. Experiments were designed to study the timing of emergence of SCN males from roots and the effects of various chemicals, including vanillic acid, and unmated SCN females of different ages on chemotaxis of SCN males of specific ages. Susceptible soybeans growing in sand were inoculated on day 7 with 3,000 to 6,000 3-day-old second-stage juveniles (J2s). After 24 hours, the roots were washed to remove sand and unpenetrated J2s. The plants were transferred to glass flasks filled with water and wrapped in aluminum foil, aerated, and grown at 27°C with 16 hours of light and 8 hours of dark. Males began emerging from roots 12 days after inoculation (DAI) and peak emergence occurred 15, 16, and 17 DAI. These males were used in chemotaxis experiments because they were most abundant and active. Emergence of males decreased until 20 DAI when collections stopped. Males were collected every 8 hours and counted to determine if photoperiod affected emergence, but all males that emerged within a 24-hour period were combined to comprise specific ages by day. Chemotaxis of males was tested using 0.001 M and 0.01 M HCl, KOH, and NaOH, 1% and 10% glycerol, serial dilutions of vanillic acid from 10^−4^ to 10^−12^ M, and unmated females collected 15, 16, and 17 DAI. Chemical treatments and individual females were placed into randomly selected reservoirs of microfluidic chemotaxis chips and deionized water was placed in the opposite reservoir of the same lane. Males were placed in the center of each lane, and the chips were incubated at 25°C in the dark for 24 hours then observed microscopically. The number of males that moved towards or away from the treatments during incubation were counted. Preliminary results indicate more SCN males emerged during 8 hours of darkness compared to the light periods. Males 16 DAI were attracted to 1% glycerol and 0.01 M NaOH. Males of all ages were not attracted or repelled by any concentration of vanillic acid, and HCl killed the nematodes. Although males were not attracted to individual SCN females, further experiments will be conducted with more females and with 0.2% agarose added to the lanes of the microfluidic chips to provide substrate to promote nematode movement. Understanding age-related interactions between SCN males and females may reveal if there is a peak mating period, and this knowledge may be useful in developing management strategies aimed at disrupting reproduction of SCN.

**Infection and Mortality of *Cylas Formicarius* by Hawaiian Isolates of *Steinernema Feltiae* and *Oscheius* SP**.

Wong, Landon, K.-H. Wang and B. S. Sipes

Department of Plant and Environmental Protection Sciences, University of Hawaii, Honolulu, HI 96822

## Abstract

Sweet potato (*Ipomea batatas*) is a New World vegetable crop planted across the world and attacked by the sweet potato weevil, *Cylas formicarius* (Coleoptera: Brentidae). The weevil larvae burrow through the tuber of the sweet potato and deposit fecal pellets resulting in an unpalatable tuber to humans and livestock. Sweet potato weevil feeding can cause yield losses up to 100% if left unmanaged. Entomopathogenic nematodes (EPN) have potential use as an organic alternative to synthetic pesticides for the control of sweet potato weevil. In Hawaii, commercial EPNs require a permit to be imported, consequently, effective local EPN isolates are desirable. Two local EPN isolates (*Steinernema feltiae* MG-14 and *Oscheius* sp. Oa-12) were evaluated for efficacy against *C. formicarius*. The objective of this experiment was to determine the mortality of *C. formicarius* larvae exposed to S. feltiae MG-14 and *Oscheius* Oa-12 in laboratory conditions. Five larvae were placed in a petri dish lined with Whatman filter paper #1 (inoculation courts) and inoculated with either 100 Infective Juveniles (IJ) of either *S. feltiae* MG-14 or *Oscheius* Oa-12 per larvae and exposed for 72 hours. Water served as a negative control. Infection courts were checked every 24 hours for larval mortality and dead larvae were transferred to White traps. EPN emergence was evaluated 21 days later. The experiment was replicated 6 times and repeated once. Larvae exposed to *S. feltiae* MG-14 had a mortality of 93% after 72 hours. Larvae exposed to *Oscheius* OA-12 had an average mortality of 43%. The negative control had a mortality of 10%. Emergence of IJ was observed from most larval cadavers from both treatments. *Steinernema feltiae* MG-14 inoculation resulted in greater mortality on the weevil larvae than *Oscheius* OA-12. The lack of emergence observed in some cadavers from inoculated plates may be due to natural death as reflected by the 10% mortality seen in the controls. Future research should examine the effectiveness the strains in field trials.

**Management of Sugar Beet Cyst Nematode in Michigan**

Yaghoubi, Ali^1^, S. Thapa^1^, B. Levene^2^ and M. Quintanilla^1^

^1^Department of Entomology, Michigan State University, East Lansing, MI, 48823

^2^Bayer Crop Science, Dewitt, MI, 48820

## Abstract

*Heterodera schachtii*, is one of the most important plant parasitic nematodes limiting sugar beet growth and production. Three separate trials were conducted in commercial fields in Michigan State. The objective of this study was to compare different pesticide effects on Sugar Beet Cyst Nematode (BCN) abundance, emergence, plant vigor, crop safety, sugar analysis and yield. As the beets were planted, as well as untreated check, several treatments were applied: in the first trial: 1- Movento 4L 2.5 fl oz/a 14 and 30 days after emergence (DAE) + Destiny HC 1.0 qt/a 14 and 30 DAE and 2- Propulse 10 fl oz/a in furrow + Movento 4L 2.5 floz/a 14 and 30 DAE + Destiny HC 1.0 qt/a 14 and 30 DAE; in second trial: 1- Abamectin 0.15 EC 3.47 fl oz/a in furrow (low rate) and 2- Abamectin 0.15 EC 6.94 fl oz/a in furrow (high rate); in third trial: 1- Velum prime 6.5 oz/a in furrow, 2- Propulse 13.6 oz/a in furrow, 3- Velum prime 6.5 oz/a in furrow + Movento HL 2.5 fl oz/a 30 DAE + Destiny HC, 4- Propulse 13.6 oz/a in furrow + Movento HL 2.5 fl oz/a 30 DAE + Destiny HC and 5- Movento HL 2.5 fl oz/a 30 DAE + Destiny HC. The initial stand counts showed significantly more sugar beets emerged for the Movento + Destiny foliars in the first trial; on the second date, three weeks after planting, the high rate of Abamectin and Velum prime had the highest number of plants emerged in the second and third trials, respectively. All plant populations were statistically similar for the last two observation dates in each trial. Plant vigor was evaluated as it related to plant development, size and overall color of the plants. From all the data collected at the harvest, there would be added value associated with using Propulse, Movento and Destiny HC on sugar beets in the first trial; there were statistical differences that favored the low-rate Abamectin over the untreated control in the second trial; and in the third trial while the treatments begun with Propulse at planting had the highest yield values, the sugar concentration for these treatments was among the lowest, also the Velum treatments had lower yields and higher sugar concentrations. Soil analyses in the first trial showed that untreated control had the highest number of juvenile (J2). Second treatment had the lowest J2 populations on average. Soil analysis for BCN is in other trials not yet completed. From these experiments, we expect to determine which pesticide provides the best control of *H. schachtii*. From this research, we can provide management suggestions to growers and potentially reduce fumigant and pesticide use.

**Update of the Guava Root-Knot Nematode *Meloidogyne Enterolobii* from Sweet Potato in North Carolina, USA**

Ye, Weimin

Nematode Assay Section, Agronomic Division, North Carolina Department of Agriculture & Consumer Services, Raleigh, NC 27607

## Abstract

*Meloidogyne enterolobii* is a recently detected and emerging root-knot nematode (RKN) species in the southeastern United States, causing severe damage to sweetpotato, soybean, and cotton. In November 2014, sweetpotato (cv. Covington) samples were collected from Johnston County, North Carolina. Fibrous and storage roots displayed significant galling damage, suggestive of RKN. Nematode extraction and quantification from accompanying soil samples indicated 1,000 to 4,200 second-stage juveniles per 500 cc soil. DNA was isolated by plucking and crushing individual female nematodes (n = 5) on a microscope slide. Sequencing of the 18S rDNA-internal transcribed spacer region 1 (KP901058), 28S rDNA D2-D3 (KP901079), and COII-16S rRNA (MN809527) was performed for species identification. BLAST searches in the GenBank database indicated sequencing results were identical to published DNA sequences of *M. enterolobii*. Koch’s postulates were completed in the greenhouse. A NC field population of *M. enterolobii* was reared on tomato (*Solanum lycopersicum* var. Rutgers) in the greenhouse. Eggs were extracted and inoculated (n = 10,000 per plant) to sweetpotato plants cvs. Covington and Beauregard. Sterile water was delivered to non-inoculated controls. Five replications of each cultivar were included in each of two separate trials. Test plants were maintained at 25-28°C for 60 days. Following this growth period, plants were uprooted and assessed for galling damage. All inoculated plants displayed galling damage (Covington = 4.7%, SD ± 1.26; Beauregard= 5%, SD ± 1.26) and no galling was observed on control plants. Eggs of *M. enterolobii* were extracted from each root system, and the average number of eggs per gram of root for Covington was 3957.17 (SD ± 815.03) and Beauregard was 4259.69 (SD ± 757.74). North Carolina is ranked as the No. 1 sweetpotato producing state in the US. From sweetpotato, *M. enterolobii* has been confirmed in limited fields in Columbus, Craven, Duplin, Edgecombe, Greene, Harnett, Jones, Johnston, Lenoir, Nash, New Hanover, Pitt, Sampson, Wake, Wayne, and Wilson counties as of September 2022. *Meloidogyne enterolobii* is a tropical species (Yang and Eisenback, 1983) that has been recorded in Florida, North Carolina, South Carolina, and Louisiana in the US (Brito et al. 2004; Ye et al. 2013; Overstreet et al. 2018; Rutter et al. 2019) and is believed to be an introduced species in North Carolina. This species is a major concern from sweetpotato growers in North Carolina and the southeastern US because it affects both yield and quality of sweetpotato. To date, there are no commercially acceptable resistant cultivars of sweetpotato available against this species, and thus *M. enterolobii* poses a significant threat to sweetpotato production in the region.

**Nematodes Associated with Mountain Pine Beetle (*Dendroctonus Ponderosae*) and Spruce Beetle (*D. Rufipennis*) in Western Canada**

Yu^1^, Qing, K. Bleiker^2^ and J. B. Tanney^2^

^1^Agriculture and Agri-Food Canada, Ottawa Research and Development Centre, Ottawa, ON, Canada

^2^Pacific Forestry Centre, Canadian Forest Service, Natural Resources Canada, Victoria, BC, Canada

## Abstract

Some species of bark beetles are serious pests in trees during outbreaks. A recent outbreak of mountain pine beetle (*Dendroctonus ponderosae*) in western North America impacted over 18 *M hectares* of forests in British Columbia, Canada, killing over half of the mature lodgepole pine in the province. Mature spruce forests in western North America are also being impacted by on-going spruce beetle (*D. rufipennis*) outbreaks. These native bark beetles are associated with numerous species, including fungi, bacteria, mites, and nematodes. The studies of nematodes and their relationships to bark beetles became an important subject hoping a biological control agent of nematode parasite can be found. This current study involved a pilot sampling of mountain pine beetle and spruce beetle to assess their respective nematode and fungal diversity, with the ultimate goal of investigating these tripartite interactions. *Dendroctonus ponderosae* and *D. rufipennis* beetles were collected from forests in one location in BC in 2021 and 2022. Nematodes were dissected from different parts of the beetles of both species and extracted from the galleries of *D. ponderosae*. Nematode species found associated with *D. ponderosae*, including those from the galleries, were *Bursaphelenchus* sp., *Ektaphelenchus* sp., *Neoditylenchus* sp., and *Pseudodiplogasteroides* sp. Interestingly clusters of dauers of *Bursaphelenchus* sp. were found on junga of the hind wings of the beetles. Nematodes dissected from *D. rufipennis* were *Bursaphelenchus* sp., *Ektaphelenchus* sp, and an unidentified Hexatylina. Nematangia consistently harboured only *Ektaphelenchus* sp. Taxonomic studies of these nematodes to species level are ongoing and future studies to extract nematodes from the galleries of *D. rufipennis* are planned.

**Identification and Characterization of Putative Effectors from *Meloidogyne Chitwoodi***

Zhang, Lei^1,2^, S. Bali^1^, J. Franco^1^, P. Vieira^3^ and C. Gleason^1^

^1^Washington State University, Department of Plant Pathology, Pullman, WA 99164

^2^ current address: Purdue University, Dept. of Botany and Plant Pathology and Dept. of Entomology, West Lafayette, IN 47907

^3^USDA-ARS, Beltsville, MD 20705

## Abstract

*Meloidogyne chitwoodi* is a root-knot nematode often found in potato growing regions. It is a nematode that can infect both potato roots and tubers, and in the case of tuber infections, it causes small pimple-like blemishes on the skin, giving the potato a rough, bumpy appearance. Unfortunately, there are no commercially available potato cultivars that are resistant to *M. chitwoodi*. To develop resistant potatoes, we must understand general plant immunity and the nematode’s ability to overcome this. One of the first lines of plant defense involves the recognition of conserved pathogen molecules, referred to as pathogen-associated molecular patterns (PAMPs). Recognition of PAMPs by surface receptors triggers complex signaling cascades leading to basal plant immunity, which is commonly called PAMP-triggered immunity (PTI). During the infection of a susceptible plant, the nematodes do not appear to activate PTI. The current theory is that nematodes secrete effectors that suppress this host defense response. We previously identified an effector in *M. hapla* called Mh265, which we showed could suppress callose deposition, a hallmark of PTI. A homologous gene called Mc265 is present in *M. chitwoodi*. In addition to Mc265, there are hundreds of predicted secreted proteins in *M. chitwoodi* whose roles in the nematode are unknown. Our goal has been to identify and characterize *M. chitwoodi* effectors. Here we show our transcriptome analysis that compares gene expression between parasitic nematodes and the non-parasitic juvenile stage. Because parasitism proteins are typically secreted by the nematodes to facilitate infection of host roots, we focused on the up-regulated genes that encoded predicted secreted proteins. We found that approximately 34% (43/127) of the genes in the predicted secretome encoded proteins with no significant homology in the public genome databases. Interestingly, 12% (15/127) of the genes that were both up regulated in expression and contained predicted secretion signal peptide sequence encoded either a known effector, putative effector or putative esophageal gland cell protein. To focus on gland-specific nematode effectors, we investigated 26 genes with relatively high expression in the juvenile stage in our transcriptome data. Using in-situ hybridization assays, we found that 7 of the 26 localized to the esophageal glands, indicating that they are secreted by the nematode. We are currently further characterizing these seven putative effectors. Once we functionally validate the putative effectors, we will use them to pull out their plant interaction partners. Targeting the plant genes that are critical for pathogen susceptibility in the plants can lead to durable resistance.

